# Network Meta-Analysis on the Effects of Traditional Chinese Exercise on Stroke Patients

**DOI:** 10.31083/RCM27104

**Published:** 2025-03-24

**Authors:** Fengwei Gao, Panpan Yan, Fengjie Qiao, Chunshun Wang, Guochun Liu, Ningning Liu, Jidong Zhang, Yongzhi Ma

**Affiliations:** ^1^Division of Sports Science and Physical Education, Tsinghua University, 100084 Beijing, China; ^2^College of Exercise Medicine, Chongqing Medical University, 400016 Chongqing, China; ^3^China Wushu School, Beijing Sport University, 100084 Beijing, China

**Keywords:** stroke, exercise training, traditional Chinese exercise, stroke recovery

## Abstract

**Background:**

Stroke is a common cerebrovascular disease characterized by a high incidence rate, significant disability, frequent recurrence, and elevated mortality. Exercise plays a crucial role in stroke rehabilitation, yet the relationship between traditional Chinese exercise and stroke recovery remains unclear. This study aims to evaluate the effectiveness of various conventional Chinese exercises through a systematic network meta-analysis and identify the most effective interventions for improving the rehabilitation outcomes of stroke patients.

**Methods:**

A systematic search was conducted in PubMed, Cochrane Library, Embase, Web of Science, China National Knowledge Infrastructure, Wanfang Data, and the China Science and Technology Journal Database (up to July 2024) to identify randomized controlled trials (RCTs) evaluating traditional Chinese exercises for stroke patients. Trials were included if they utilized at least one form of traditional Chinese exercise. The methodological quality of the included studies was assessed using the Cochrane Risk of Bias tool (ROB 2.0). Data analysis was performed using Stata 17.0 and the Mvmeta package, employing a random-effects model.

**Results:**

A total of 43 studies involving 2083 stroke patients were included. These studies assessed outcomes including upper limb motor function, lower limb motor function, overall motor ability, walking ability, balance ability, self-care ability, cognitive function, depression, quality of life, and sleep quality. Baduanjin, originating in the Song Dynasty and consisting of eight movements based on traditional Chinese medicine theories,was the most effective in improving upper limb motor function, overall motor ability, walking ability, self-care ability, cognitive function, quality of life, and sleep quality. Taiji, a practice integrating Chinese philosophy, martial arts, and wellness concepts, was the most effective in enhancing lower limb motor function. Wuqinxi, inspired by the dynamic movements of animals such as the tiger, deer, bear, apes, and birds, showed the best results for balance improvement. Liuzijue, a traditional exercise combining specific sound production, breathing, and movement, was most effective in alleviating depressive symptoms.

**Conclusions:**

These findings suggest that Baduanjin may be the most effective intervention for stroke rehabilitation. However, further high-quality RCTs are required to confirm these results.

**The PROSPERO registration:**

CRD42024566780, https://www.crd.york.ac.uk/PROSPERO/view/CRD42024566780.

## 1. Introduction

Stroke is the second leading cause of death globally and the third of combined death and disability, with global stroke-related costs exceeding USD 721 billion. From 1990 to 2019, global stroke deaths increased by 43.0%, prevalence rose by 102.0%, and disability-adjusted life years (DALYs) increased by 143.0% [1]. Major risk factors for stroke include hypertension, dyslipidemia, diabetes, sedentary lifestyle, obesity, excessive alcohol consumption, and tobacco use. Stroke is typically caused by neurological dysfunction due to ischemia or hemorrhage [2], often leading to sequelae such as hemiplegia, aphasia, cognitive dysfunction, weakness, and motor impairments that significantly reduce quality of life [3]. Additionally, stroke patients are prone to complications such as infections and depression, further increasing their physical and psychological burden [4]. As the global population ages, the medical and economic burden of stroke will become a significant challenge for society and families [5, 6, 7, 8, 9, 10, 11]. Therefore, improving the prognosis and quality of life of stroke patients is essential.

One of the primary difficulties in post-stroke rehabilitation lies in the restoration of motor function, especially in the upper and lower limbs, which are frequently impaired due to neural damage. Methods to promote stroke rehabilitation include pharmacological treatments, surgical interventions, neurostimulation therapies, and exercise and physiotherapy [12]. Pharmacological treatments are commonly used in the acute phase, primarily through anticoagulants, thrombolytic agents, and neuroprotective drugs to mitigate brain damage; however, long-term use may be associated with bleeding risks and side effects [13, 14]. Surgical interventions are used in certain cases, such as severe ischemia or cerebral hemorrhage, where procedures such as thrombectomy or hematoma removal can improve outcomes [15, 16]. Neurostimulation therapies, such as transcranial magnetic stimulation (TMS) and deep brain stimulation (DBS), promote functional recovery by regulating neural activity [17, 18]; however, treatment outcomes vary among individuals, and the high cost of equipment makes widespread application difficult. Furthermore, long-term efficacy requires verification [19]. Cognitive rehabilitation, which addresses attention, memory, and executive function deficits, faces similar obstacles, as current approaches often yield inconsistent results. Additionally, mental health issues such as depression, anxiety, and post-stroke emotional changes are common and frequently exacerbated by the physical limitations imposed by the stroke, further complicating rehabilitation efforts. Exercise has been used extensively in post-stroke rehabilitation and has positively affected cognitive function, balance, gait, and quality of life in stroke patients [20, 21, 22, 23]. Research suggests that appropriate exercise can accelerate neurological recovery after stroke, contributing to rehabilitation [19]. The effectiveness of different exercise interventions varies depending on their type, mode, and dosage, directly impacting stroke prognosis [24].

Traditional Chinese exercises, with a history spanning over 5000 years, mainly include Taiji, Baduanjin, Yijin jing, Wuqinxi, and Liuzijue. These practices combine posture control, breathing techniques, and mindfulness, aiming to enhance physical and mental health by improving meridian flow and blood circulation, and have been widely applied in stroke rehabilitation [25, 26]. Traditional Chinese exercises improve balance and gait in stroke patients and enhance quality of life and mental health [23, 27, 28]. Therefore, these interventions offer safe and effective non-pharmacological options, particularly suitable for long-term rehabilitation.

Despite their widespread use in Asia, evidence of their comparative effectiveness remains fragmented and inconsistent. While some studies report significant improvements with specific exercises, others yield inconclusive or contradictory findings. Indeed, some studies have indicated that Taiji significantly improves balance, walking ability, and daily activities in stroke patients [29, 30, 31], whereas Li [32] and Liu [33] suggested that Taiji had no significant impact on walking ability or sleep quality in stroke patients.

Furthermore, existing reviews primarily focus on isolated forms of exercise, neglecting comprehensive comparisons across modalities and their relative rankings for various rehabilitation outcomes. This lack of clarity hinders clinical decision-making and the integration of these interventions into evidence-based stroke rehabilitation protocols [31]. To address these gaps, this research utilized a network meta-analysis to systematically evaluate and compare the efficacy of various traditional Chinese exercise methods in promoting stroke rehabilitation to offer relative rankings across multiple functional domains and provide actionable insights for clinicians and policymakers.

## 2. Materials and Methods

This study was designed and reported in accordance with the Network Meta-Analysis extension of the Preferred Reporting Items for Systematic Review and Meta-Analysis 2015 statement (PRISMA-NMA). The research has been formally registered in PROSPERO and assigned the identifier CRD42024566780.

### 2.1 Search Strategy

A comprehensive search was conducted across seven databases: PubMed, Cochrane Library, Embase, Web of Science, China National Knowledge Infrastructure (CNKI), Wanfang Data, and the China Science and Technology Journal Database, covering literature up to July 8, 2024. The search employed a combination of Medical Subject Headings (MeSH) terms and free-text terms tailored to each database to maximize sensitivity and specificity. The primary search terms included: Stroke-related terms: “Stroke”, “Cerebrovascular Accident”, “Cerebrovascular Disease”, “CVA”, “Cerebral Apoplexy”, “Cerebral Infarction”, “Cerebral Disease”, “Cerebral Ischemia”; exercise-related terms: “Traditional Exercise”, “Health-Cultivation Exercise”, “Taiji”, “Baduanjin”, “Liuzijue”, “Yijinjing”, “Wuqinxi”, “Daoyin”, “Qigong”.

To enhance the comprehensiveness of the search, database-specific adjustments were made to account for unique indexing and features. For example, in PubMed, Boolean operators combined MeSH terms and free-text terms as follows: (“Stroke” [MeSH] OR “Cerebrovascular Accident” OR “Cerebral Infarction”) AND (“Traditional Chinese Exercise” [MeSH] OR “Taiji” OR “Baduanjin”). In the CNKI and Wanfang Data, Chinese keywords and phrases were used to reflect local terminologies for traditional Chinese exercises. Additionally, wildcard symbols and truncation (e.g., “exercise*”) were applied in Embase to capture variant spellings and derivatives. We manually screened references of relevant systematic reviews and meta-analyses to ensure comprehensive coverage. The full search strategy, including specific syntax for each database, is available in **Supplementary Table 1**.

### 2.2 Inclusion Criteria and Literature Screening

We conducted the literature screening based on the PICOS guidelines. The detailed inclusion criteria are as follows: (1) Population: stroke patients aged ≥18 years, diagnosed according to national or international stroke diagnostic criteria. (2) Interventions and comparisons: The experimental group must involve at least one form of traditional Chinese exercise, such as Baduanjin, Wuqinxi, Taiji, Liuzijue, Daoyin, or Qigong. The control group includes routine care, placebo, or conventional rehabilitation training. Routine care refers to health education, daily care, and living assistance, while conventional rehabilitation includes balance training, strength training, or flexibility training techniques. (3) Outcomes: the studies must assess outcomes related to physical abilities, mental health, self-care ability, and quality of life. (4) Study design: only randomized controlled trials (RCTs) were included. Exclusion criteria included duplicate publications, conference abstracts, studies without full-text availability, or studies with unextractable data. The identified studies were initially imported into the Endnote X9 software (Clarivate Analytics, Philadelphia, PA, USA) to identify and remove duplicates. Two independent reviewers then conducted an initial screening based on titles and abstracts, followed by a full-text screening. Any disagreements between the reviewers were resolved by consulting a third researcher for arbitration.

### 2.3 Data Selection and Extraction

Two independent researchers, Wang Chunshun and Liu Guochun, independently extracted data using a predefined structured data extraction form. The extracted information included basic study details, characteristics of the study population, risk of bias assessment, outcome measures, and data used for analysis. The most recent data were incorporated in cases where multiple publications stemmed from the same trial. Any discrepancies arising during data extraction were resolved through a discussion between the independent reviewers. If necessary, a third researcher, FWG, was consulted to facilitate consensus and ensure data accuracy. The extracted data included the means and standard deviations or the standard error of the means from the included studies. If there was uncertainty regarding critical information or data from the included studies, we emailed the original study authors to obtain the necessary data for this research.

### 2.4 Risk of Bias Assessment

The included studies were analyzed for potential bias using the Cochrane Risk of Bias 2.0 (ROB 2) instrument [34]. The Cochrane ROB 2 framework examines five primary domains: Bias stemming from the randomization process, deviations from planned interventions, missing outcome data, outcome measurement, and the selection of reported results. For each domain, bias is categorized into three levels to ensure a structured assessment of potential biases: Low risk, some concerns, or high risk. If all domains were rated as low risk, the overall risk of bias for the study was classified as low. If some domains were rated as some concerns but no domain was rated as high risk, the overall risk of bias was classified as some concerns. If any domain was rated as high risk, the overall risk of bias in the study was classified as high; otherwise, it was deemed unclear. Two researchers independently assessed the risk of bias and the level of evidence. In cases of disagreement, a third researcher was consulted to reach a consensus.

### 2.5 Certainty of Evidence Assessment

The certainty of the evidence was assessed using the Confidence in Network Meta-Analysis (CINeMA) tool [35], which evaluates the quality of evidence in network meta-analyses. This tool is based on the Grades of Recommendation, Assessment, Development, and Evaluation (GRADE) system and was calculated using the Netmeta package in R software (Version 4.4.2, R Foundation for Statistical Computing, Vienna, Austria), following a frequentist approach. CINeMA assesses six domains: Within-study bias, reporting bias, indirectness, imprecision, heterogeneity, and incoherence. Each domain is rated as having no concerns (no downgrade), some concerns (downgraded by one level), or major concerns (downgraded by two levels). Using these evaluations, the overall level of certainty of the evidence is categorized as high certainty of evidence, moderate certainty of evidence, low certainty of evidence, or very low certainty of evidence.

### 2.6 Statistical Analysis

The mean and standard deviation differences before and after interventions were calculated to facilitate comparisons. If a study did not report mean and standard deviation values, these were indirectly calculated using baseline and endpoint values using formulas recommended by the Cochrane Handbook. As the included studies involved continuous data, the effect size was expressed as the mean difference (MD) when the measurement tools were consistent and as the standardized mean difference (SMD) when different measurement tools were used, with a 95% confidence interval (CI). Network meta-analysis was conducted using the Network and Mvmeta packages in Stata 17.0 (StataCorp, College Station, TX, USA), using a random-effects frequentist framework. A network evidence diagram was created, with the node size reflecting the sample size for each intervention and the link width representing the number of trials conducted to compare the respective interventions.

Heterogeneity between studies was assessed using both Q-tests and I^2^ values. Based on the magnitude of the I^2^ value, a fixed-effects model or a random-effects model was selected for meta-analysis. A random-effects model was employed when I^2^ was >50%, indicating significant heterogeneity. Conversely, a fixed-effects model was used when I^2^ was ≤50%, suggesting minimal heterogeneity. If the number of studies was ≥10, a funnel plot was drawn to assess publication bias. Symmetry in the funnel plot indicated the absence of publication bias.

Global inconsistency was tested first, and if closed loops were formed, local inconsistency was evaluated through the node-splitting method. A *p*-value > 0.05 indicated no significant inconsistency. The interventions were ranked using the surface under the cumulative ranking (SUCRA) curve, with SUCRA values ranging from 0% to 100%; the higher the SUCRA value, the better the effect of the intervention. Heterogeneity was evaluated by comparing the statistical significance of the actual intervals in pairwise comparisons with the predicted intervals. If the statistical significance was similar, heterogeneity was considered low, and high if different. Box plots were generated, considering seven effect modifiers (sample size, age, disease duration, gender, intervention duration, intervention frequency, and intervention period) to evaluate transitivity across studies. Sensitivity analysis was conducted by excluding studies with a high risk of bias and those with sample sizes below 20. If the results remained unchanged, the analysis was considered stable.

## 3. Results

### 3.1 Literature Screening Process and Results

**Fig. 1.**
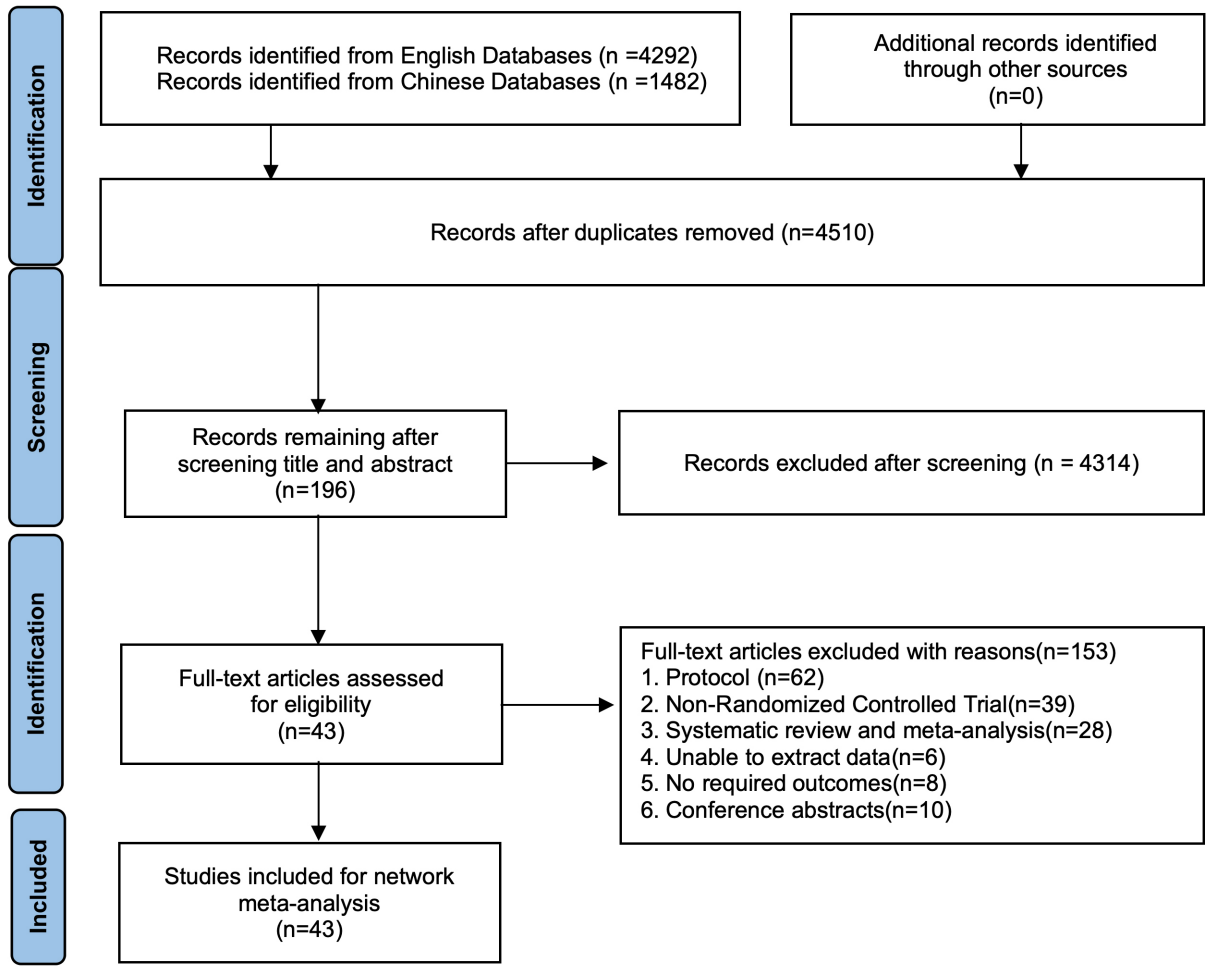
**PRISMA flow diagram for study selection**. PRISMA flow diagram illustrating the study selection process. PRISMA-NMA, Network Meta-Analysis extension of the Preferred Reporting Items for Systematic Review and Meta-Analysis 2015 statement.

The initial search yielded 5774 articles. After removing duplicates, 4510 articles remained. Following a preliminary screening based on titles and abstracts, 196 articles were selected for full-text review. After a thorough assessment, 43 RCTs were ultimately included in the analysis (**Supplementary 10**). Fig. 1 presents the PRISMA flow diagram, which outlines the screening process details.

### 3.2 Literature Screening Process and Results

The included studies were published between January 2009 and May 2024 and encompassed 2083 stroke patients. The average age of the participants was 59.98 ± 6.06 years, with women accounting for 33.79% of the sample. The mean duration of illness was 9.89 ± 14.03 months. The research was conducted in China, the United States, Japan, and South Korea, with most studies originating from China. One study utilized a three-arm trial design, while the others followed a two-arm trial format. The intervention groups involved eight types of traditional Chinese exercises. Specifically, 12 studies used Baduanjin, 19 used Taiji, 7 used Liuzijue, 4 used Yijinjing, 1 used Daoyin, and 1 used Wuqinxi. The control groups employed two types of interventions: 15 studies used routine care, and 28 studies used conventional rehabilitation as the control interventions. The average intervention duration was 9.77 ± 8.90 weeks, with most studies implementing the intervention 5 times per week, for an average of 40 minutes per session, as shown in Table 1 (Ref. [36, 37, 38, 39, 40, 41, 42, 43, 44, 45, 46, 47, 48, 49, 50, 51, 52, 53, 54, 55, 56, 57, 58, 59, 60, 61, 62, 63, 64, 65, 66, 67, 68, 69, 70, 71, 72, 73, 74, 75, 76, 77, 78]).

**Table 1.** **The general information of the included studies**.

Study	Location	Type of intervention	Sample	Age (years)	Time post-stroke (months)	Female	Intervention duration (weeks)	Intensity/period of intervention	Outcomes	Risk of bias
Au *et al*, 2009 [36]	China	Taiji conventional care	59	61.7 ± 10.5	54.1 ± 79.2	44.1%	12	2 times/week, 120 min/time	Walking ability: TUG	High
			55	65.9 ± 10.7	64.2 ± 106.4	40.0%				
Taylor and Coull, 2012 [37]	United States						12	3 times/week, 60 min/time	Walking ability: SPPB	Low
			13	72.8 ± 10.1	58.3 ± 46.7	38.0%			Balance ability: SPPB	
			12	64.5 ± 10.9	47.9 ± 42.5	42.0%			Depression: CES-D	
									Quality of life: SF-36	
									Sleep quality: PSQI	
Taylor *et al*, 2014 [38]	United States				>3		12	3 times/week, 60 min/time	Walking ability: SPPB	Low
			53	71.5 ± 10.3		35.8%			Balance ability: SPPB	
			48	68.2 ± 10.3		52.1%			Depression: CES-D	
									Quality of life: SF-36	
									Sleep quality: PSQI	
Chen *et al*, 2019 [39]	China		36	67.0 ± 15.1	NA	41.7%	1	7 times/week, 15 min/time	Quality of life: SF-12	Low
			36	67.8 ± 12.6	NA	36.1%				
Song *et al*, 2021 [40]	South Korea						26	2 times/week, 50 min/time	Walking ability: FAC	Some concerns
			18	58.7 ± 17.1	NA	44.4%			Balance ability: BBS	
			16	57.2 ± 10.7	NA	31.3%			Self-care ability: Barthel	
									Cognitive function: MoCA	
									Quality of life: SS-QOL	
Wang *et al*, 2010 [41]	Japan	Taiji conventional rehabilitation	16	76.5 ± 9.7	NA	69.2%	12	1 times/week, 50 min/time	Sleep quality: PSQI	Some concerns
			13	77.6 ± 12.3	NA	76.4%				
Kim *et al*, 2015 [42]	South Korea		11	53.5 ± 11.5	NA	36.4%	6	2 times/week, 60 min/time	Walking ability: TUG	Some concerns
			11	55.2 ± 10.2	NA	45.5%			Balance ability: DGI	
									Quality of life: SF-36	
Fu and Zhang, 2016 [43]	China		30	59.7 ± 7.6	NA	36.7%	8	7 times/week, 40 min/time	Walking ability: FAC	Some concerns
			30	60.3 ± 8.4	NA	40.0%			Balance ability: BBS	
Wang *et al*, 2016 [44]	China		14	60.7 ± 7.3	15.1 ± 8.5	35.7%	12	5 times/week, 60 min/time	Balance ability: BBS	Some concerns
			16	58.6 ± 8.5	25.3 ± 21.4	12.5%				
Zhao* et al*, 2017 [45]	China		30	53.9 ± 11.7	1.3 ± 0.8	36.7%	8	5 times/week, 30 min/time	Motor ability: overall FMA	Some concerns
			30	51.4 ± 14.8	1.4 ± 0.7	33.3%			Self-care ability: Barthel	
									Depression: HAMD	
Xie *et al,* 2018 [46]	China						12	5 times/week, 60 min/time	Motor ability: overall FMA	Low
			120	60.9 ± 8.7	14.5 ± 18.1	30.8%			Walking ability: TUG	
			124	60.1 ± 8.6	14.3 ± 22.1	20.2%			Balance ability: BBS	
									Self-care ability: Barthel	
									Depression: BDI; quality of life: SF-36	
Jiang* et al*, 2018 [47]	China	Taiji conventional rehabilitation	30	58.8 ± 11.7	3.6 ± 2.0	23.3%	9	5 times/week, 30 min/time	Motor ability: upper extremity FMA	Some concerns
			30	56.5 ± 12.8	3.3 ± 1.8	26.7%				
Huang* et al*, 2019 [48]	China		14	62.2 ± 9.7	11.4 ± 4.9	28.6%	12	3 times/week, 40 min/time	Motor ability: lower extremity FMA	Low
			14	59.9 ± 10.0	10.5 ± 4.2	14.3%			Balance ability: m-CTSIB	
Ku *et al*, 2020 [49]	China		10	55.0 ± 7.3	9.5 ± 11.0	30.0%	6	3 times/week, 60 min/time		Low
			10	52.5 ± 6.3	22.0 ± 19.8	30.0%			Motor ability: lower extremity FMA	
Yu *et al*, 2020 [50]	China		35	63.0 ± 8.9	11.4 ± 5.2	40.0%	12	3 times/week, 40 min/time	Balance ability: BBS	Low
			36	58.7 ± 9.7	9.4 ± 4.8	38.9%				
He *et al*, 2022 [51]	China		26	62.5 ± 10.7	NA	23.1%	4	4 times/week, 40 min/time	Motor ability: upper extremity FMA	Some concerns
			29	63.0 ± 9.0	NA	20.7%			Walking ability: FAC	
									Balance ability: BBS	
Tang *et al*, 2022 [52]	China						8	5 times/week, 30 min/time	Motor ability: upper extremity FMA	Some concerns
			33	54.9 ± 13.1	1.2 ± 0.4	36.4%			Walking ability: TUG	
			34	56.5 ± 11.2	1.2 ± 0.4	41.2%			Balance ability: BBS	
									Self-care ability: Barthel	
Wang *et al*, 2023 [53]	China		10	49.1 ± 11.9	2.2 ± 1.1	20.0%	4	7 times/week, 30 min/time	Motor ability: upper extremity FMA	Some concerns
			10	52.9 ± 11.8	1.8 ± 1.2	20.0%			Self-care ability: Barthel	
Yang and Liu, 2019 [54]	China	Baduanjin and Taiji conventional rehabilitation	23	52.9 ± 32.9	1.8 ± 1.9	42.9%	4	5 times/week, 40 min/time	Motor ability: lower extremity FMA	Some concerns
			28	51.4 ± 47.6	1.9 ± 2.0	39.3%				
			21	54.0 ± 38.4	1.9 ± 2.0	38.1%				
CAI, 2010 [55]	China	Baduanjin conventional care	30	60.3 ± 10.5	NA	33.3%	12	4–5 times/week, 30 min/time	Quality of life: WHOQOL-BREF	Some concerns
			30	61.3 ± 7.4	NA	23.3%				
Lv* et al*, 2019 [56]	China		85	60.5 ± 5.3	>3	63.5%	12	5 times/week, 60 min/time	Sleep quality: PSQI	Some concerns
			85	59.8 ± 4.3		64.7%				
Zheng *et al*, 2020 [57]	China		24	61.6 ± 9.2	6.5 ± 2.1	20.8%	28	3 times/week, 40 min/time	Self-care ability: Barthel	Low
			24	62.8 ± 6.4	6.7 ± 2.3	8.3%			Cognitive function: MoCA	
Ye *et al*, 2022 [58]	China						24	3 times/week, 40 min/time	Motor ability: upper extremity FMA	Some concerns
			24	61.6 ± 9.2	6.5 ± 2.1	20.8%			Lower extremity FMA	
			23	61.6 ± 9.2	6.7 ± 2.3	8.3%			Overall FMA	
									Balance ability: BBS	
Yang* et al,* 2023 [59]	China		15	52.9 ± 32.9	1.8 ± 1.9	26.7%	8	3 times/week, 40 min/time	Quality of life: WHOQOL-BREF	Some concerns
			15	54.0 ± 38.4	1.91 ± 2.01	33.3%				
Liu* et al*, 2024 [60]	China		50	58.9 ± 10.8	2.8 ± 1.5	38.0%	8	10 times/week, 30 min/time	Self-care ability: Barthel	Low
			50	56.2 ± 11.5	2.6 ± 1.3	42.0%			Depression: HAMD	
Zhang and Huang, 2021 [61]	China	Baduanjin conventional rehabilitation					8	5 times/week	Motor ability: upper extremity FMA	Some concerns
									Lower extremity FMA	
			41	71.3 ± 4.5	7.2 ± 1.8	43.9%			Overall FMA	
			41	70.5 ± 4.3	7.3 ± 1.9	41.5%			Walking ability: 6MWT	
									Self-care ability: Barthel	
									Quality of life: SF-36	
Yuen* et al*, 2021 [62]	China		29	63.1 ± 10.6	23.1 ± 21.5	48.3%	16	3 times/week, 50 min/time	Walking ability: TUG	Low
			29	62.0 ± 13.1	25.3 ± 21.6	51.7%			Self-care ability: Barthel	
									Quality of life: SS-QOL	
Liu *et al*, 2022 [63]	China		23	59.2 ± 4.7	1.9 ± 0.7	27.7%	4	6 times/week, 30 min/time	Walking ability: TUG	Low
			23	57.7 ± 5.6	2.1 ± 0.8	39.1%			Balance ability: BBS	
Ding *et al*, 2020 [64]	China		68	46.3 ± 6.6	NA	27.9%	52	7 times/week, 50 min/time	Motor ability: overall FMA	Some concerns
			48	47.2 ± 7.5	NA	31.3%			Self-care ability: Barthel	
									Cognitive function: MMSE	
Chen* et al*, 2024 [65]	China		21	52.9 ± 14.8	7.0 ± 13.3	23.5%	4	5 times/week, 40 min/time	Motor ability: upper extremity FMA	Some concerns
			21	54.1 ± 12.3	6.12 ± 7.81	23.5%			Balance ability: BBS	
									Self-care ability: Barthel	
Zheng *et al*, 2021 [66]	China	Liuzijue conventional rehabilitation	30	63.5 ± 10.4	0.9 ± 0.5	20.0%	3	5 times/week, 45 min/time	Balance ability: BBS	Low
			30	67.2 ± 9.2	1.0 ± 47.3	36.7%				
Zhang *et al*, 2022 [67]	China		80	65.4 ± 9.2	2.2 ± 1.6	30.0%	2	5 times/week, 45 min/time	Motor ability: overall FMA	Low
			80	62.8 ± 11.2	2.6 ± 2.1	20.0%			Balance ability: BBS	
									Self-care ability: Barthel	
Wang *et al*, 2022 [68]	China		32	65.3 ± 9.2	2.0 ± 1.5	25.0%	4	5 times/week, 20 min/time	Motor ability: overall FMA	Some concerns
			31	60.7 ± 12.2	2.4 ± 1.8	22.6%			Balance ability: BBS	
									Self-care ability: Barthel	
Zheng *et al*, 2023 [69]	China		27	66.5 ± 11.1	NA	26.9%	12	5 times/week, 22 min/time	Depression: HAMD	Some concerns
			25	64.6 ± 11.0	NA	28.0%				
Du *et al*, 2021 [70]	China	Liuzijue conventional care	30	50.6 ± 9.6	3.1 ± 0.2	40.0%	4	14 times/week, 15–20 min/time	Depression: HAMD	Some concerns
			30	48.3 ± 8.2	3.1 ± 0.2	43.3%				
Xia *et al*, 2021 [71]	China		25	57.3 ± 9.4	2.5 ± 0.4	20.0%	4	5 times/week, 20 min/time	Cognitive function: MoCA	Some concerns
			25	59.0 ± 11.4	2.2 ± 0.8	24.0%				
Xia *et al*, 2023 [72]	China		35	57.9 ± 9.4	10.6 ± 1.61	17.1%	4	5 times/week, 40 min/time	Cognitive function: MoCA	Some concerns
			35	58.2 ± 11.8	9.1 ± 3.2	20.0%				
Li *et al*, 2012 [73]	China	Yijinjing conventional rehabilitation	30	NA	NA	47.2%	5	2 times/week, 30 min/time	Depression: HAMD	Some concerns
			30	NA	NA	52.8%				
Zhang* et al*, 2020 [74]	China		25	57.9 ± 13.8	NA	20.0%	4	5 times/week, 40 min/time	Quality of life: SF-36	Some concerns
			25	59.3 ± 11.8	NA	24.0%			Sleep quality: PSQI	
Sun *et al*, 2022 [75]	China		30	62.0 ± 7.4	8.0 ± 5.3	43.3%	3	7 times/week, 60 min/time	Depression: HAMD	Low
			30	65.2 ± 6.3	7.5 ± 4.1	43.3%				
Sun *et al*, 2022 [76]	China		30	62.0 ± 7.4	NA	43.3%	4	7 times/week, 45 min/time		Some concerns
			30	65.2 ± 6.3	NA	43.3%				
Zhang* et al*, 2023 [77]	China	Wuqinxi conventional rehabilitation	28	60.2 ± 9.0	2.6 ± 0.5	40.6%	13	5 times/week, 40 min/time	Balance ability: BBS	Some concerns
			32	60.3 ± 9.0	2.6 ± 0.5	46.4%				
Tian *et al*, 2024 [78]	China	Daoyin conventional care					2	10 times/week, 40 min/time	Motor ability: upper extremity FMA	Low
									Lower extremity FMA	
			25	58.0 ± 9.0	9 ± 5	16.0%			Overall FMA	
			25	58.0 ± 12.0	7 ± 4	12.0%			Balance ability: BBS	
									Self-care ability: Barthel	
									Depression: HAMD	
									Sleep quality: PSQI	

FMA, Fugl-Meyer Assessment scale; FAC, functional ambulation category scale; 6MWT, 6-minute walk test; BBS, Berg Balance Scale; m-CTSIB, Modified Clinical Test of Sensory Interaction on Balance; DGI, Dynamic Gait Index; Barthel, Barthel Index of Activities of Daily Living; MMSE, mini-mental state examination; MoCA, Montreal Cognitive Assessment; HAMD, Hamilton Depression Rating Scale; CES-D, Center for Epidemiologic Studies Depression Scale; BDI, Beck Depression Inventory; SF-12, 12-item short-form health survey; SF-36, 36-item short-form health survey; SS-QOL, Stroke-Specific Quality of Life Scale; WHOQOL-BREF, World Health Organization Quality of Life – BREF; SPPB, Short Physical Performance Battery; TUG, Timed Up and Go Test; PSQI, Pittsburgh Sleep Quality Index; NA, not applicable.

### 3.3 Risk of Bias

**Fig. 2.**
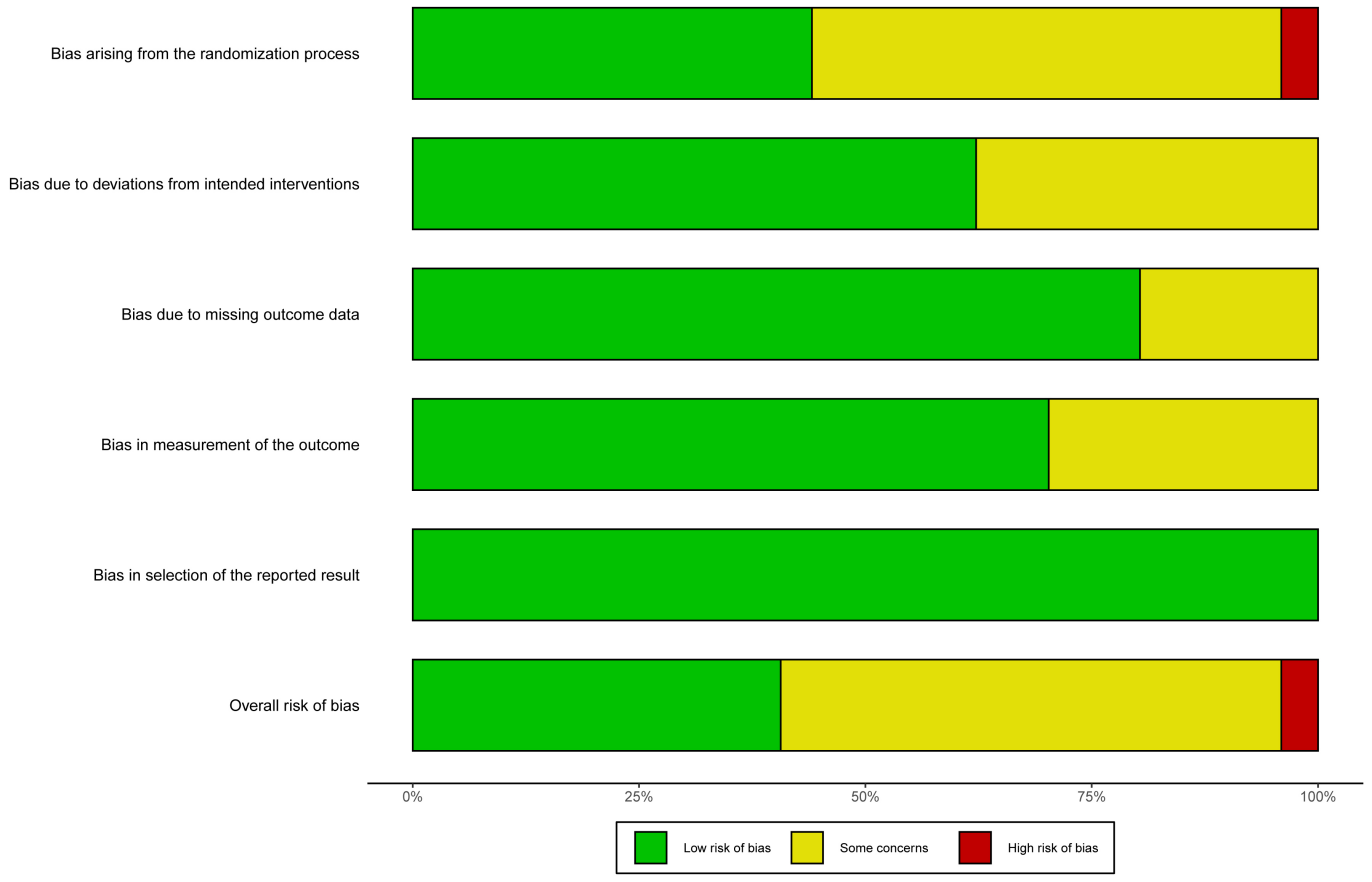
**Overall risk of bias**. The risk of bias assessment across five domains: bias originating from the randomization process, bias resulting from deviations in intended interventions, bias resulting from incomplete outcome data, bias in the assessment of outcomes, and bias in the selection of the reported outcomes. The green, yellow, and red segments represent studies with a low risk of bias, some concerns, and a high risk of bias, respectively. The overall risk of bias shows a combination of studies with low risk and some concerns, with a small proportion having a high risk of bias.

**Fig. 3.**
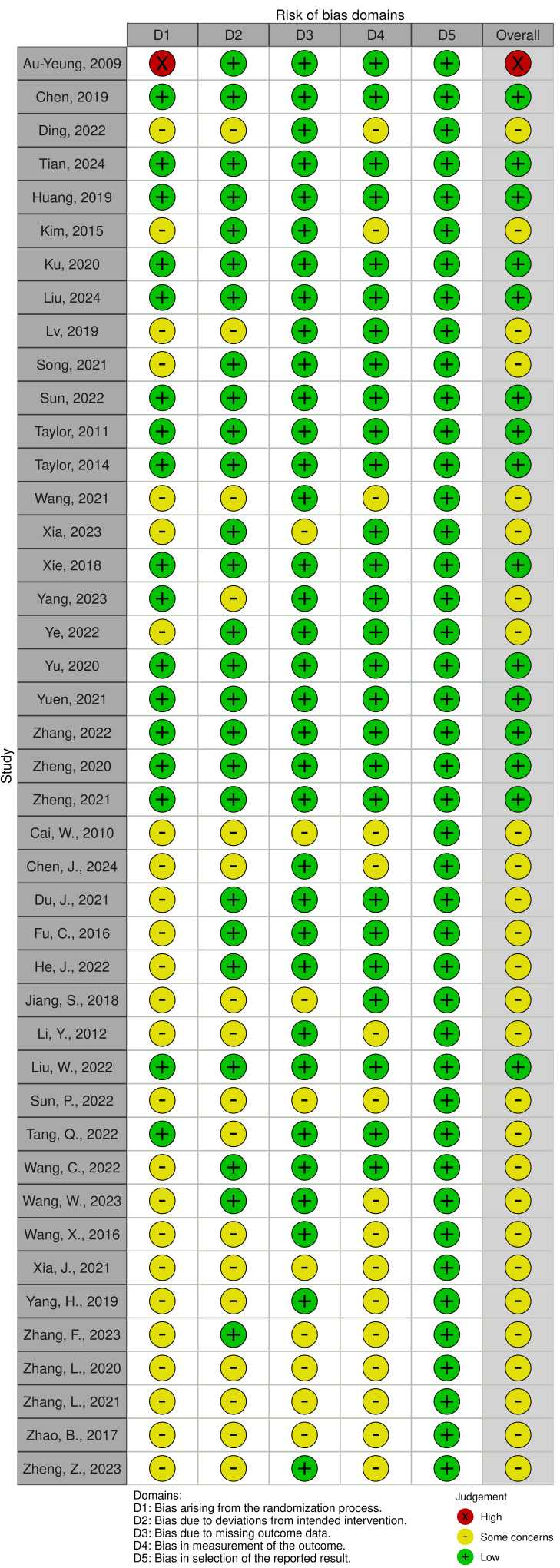
**Risk of bias summary for individual studies**. A detailed breakdown of the risk of bias across individual studies is included in the analysis. Each row represents a specific domain of bias, including bias arising from the randomization process, bias due to deviations from intended interventions, bias due to missing outcome data, bias in the measurement of the outcome, and bias in the selection of the reported result. Each column corresponds to an individual study, with green, yellow, and red circles indicating low bias risk, moderate concerns, and high bias risk, respectively. The overall assessment shows that most studies fall into low risk or some concerns of bias.

One study was rated as having a high risk of bias due to the lack of details regarding allocation concealment and baseline imbalances. Fifteen studies were assessed as having a low risk of bias. The remaining 27 studies were rated as having some concerns due to unclear randomization and allocation concealment methods, a lack of reporting on participant dropout during the study, and unclear reporting on how the blinding of interventionists and outcome assessors was conducted. These risk assessments are illustrated in Figs. 2,3.

### 3.4 Network Evidence Diagram

**Fig. 4.**
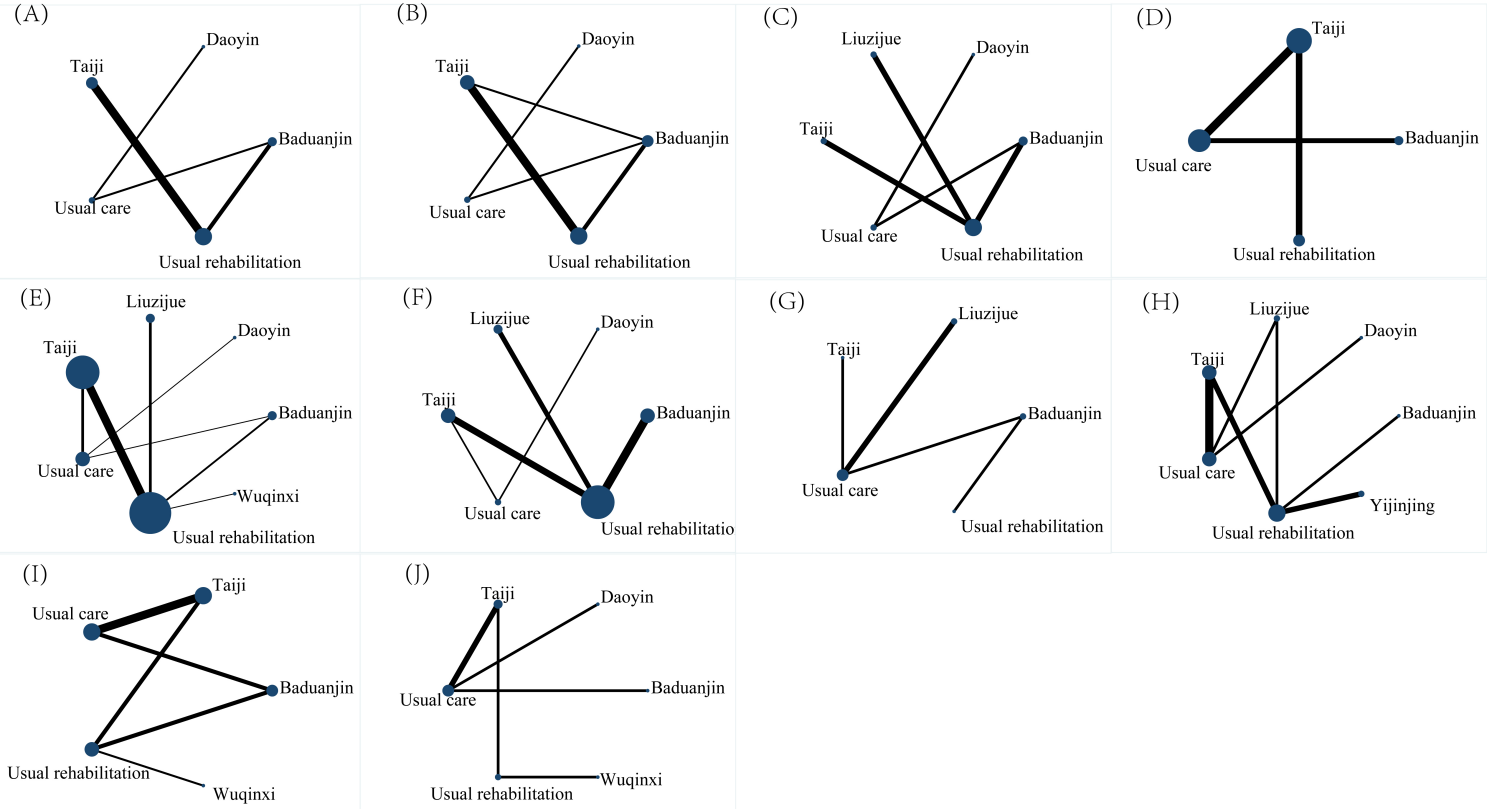
**Network evidence diagrams for various outcomes in stroke rehabilitation**. (A) Upper limb motor ability. (B) Lower limb motor ability. (C) Overall motor ability. (D) Walking ability. (E) Balance ability. (F) Self-care ability. (G) Cognitive function. (H) Depression. (I) Quality of life. (J) Sleep quality. Each network diagram illustrates the comparisons between different interventions. The size of the nodes is proportional to the sample size of the intervention; the thickness of the connecting lines represents the number of studies directly comparing the two interventions.

The 43 studies reported a total of 10 outcome indicators, resulting in the creation of 10 network evidence diagrams (Fig. 4). In these diagrams, the size of each node is proportional to the sample size of the intervention, while the lines connecting the nodes indicate the existence of direct comparisons between interventions. The width of each line is proportional to the number of direct comparisons conducted. Detailed network evidence diagrams for each of the 10 outcome indicators are provided in the supplementary materials (**Supplementary Figs. 1–10**).

### 3.5 Network Meta-Analysis 

#### 3.5.1 Upper Limb Motor Ability

In terms of improving upper limb motor ability, pairwise comparison results showed that Baduanjin (MD = 4.56, 95% CI = 2.78, 6.35, *p* < 0.05) and Daoyin (MD = 2.65, 95% CI = 0.75, 4.54, *p* < 0.05) were more effective than conventional rehabilitation. Baduanjin was also more effective than routine care (MD = 4.79, 95% CI = 0.09, 9.49, *p* < 0.05). While the improvement was statistically significant, clinical significance depends on whether these changes surpass established thresholds, such as a 10-point increase on the Fugl-Meyer Assessment scale, typically associated with meaningful functional recovery. The observed mean difference of 4.56 may represent modest benefits and should be interpreted cautiously. In the other comparisons, no statistically significant differences were observed (*p* > 0.05). The SUCRA ranking results indicated that Baduanjin was the most effective intervention for improving upper limb motor ability (SUCRA = 91.5%), followed by Taiji (SUCRA = 62.7%), Daoyin (SUCRA = 51.1%), routine care (SUCRA = 22.9%), and conventional rehabilitation (SUCRA = 21.7%) (see Table 2).

**Table 2.** **Results of pairwise comparisons**.

**Upper limb motor ability (MD (95% CI))**						
Baduanjin (91.5%)						
2.79 (–5.11, 10.69)	Taiji (62.7%)					
1.92 (–0.75, 4.59)	–0.87 (–9.21, 7.47)	Daoyin (51.1%)				
4.56 (2.78, 6.35)*	1.77 (–6.33, 9.87)	2.65 (0.75, 4.54)*	Usual care (22.9%)			
4.79 (0.09, 9.49)*	2.00 (–4.36, 8.36)	2.87 (–2.53, 8.28)	–0.23 (–5.25, 4.80)	Usual rehabilitation (21.7%)		
**Lower limb motor ability (MD (95% CI))**						
Taiji (91.8%)						
1.38 (–1.20, 3.96)	Baduanjin (70.7%)					
3.00 (–3.10, 9.11)	1.62 (–3.92, 7.16)	Daoyin (50.6%)				
3.94 (1.90, 5.98)*	2.56 (0.33, 4.78)*	0.94 (–5.03, 6.90)	Usual rehabilitation (30.0%)			
6.00 (1.35, 10.66)*	4.62 (0.75, 8.49)*	3.00 (–0.96, 6.96)	2.06 (–2.40, 6.53)	Usual care (6.8%)		
**Overall motor ability (MD (95% CI))**						
Baduanjin (91.9%)						
8.50 (–10.22, 27.22)	Daoyin (69.9%)					
12.68 (–14.71, 40.07)	4.18 (–29.00, 37.36)	Liuzijue (55.5%)				
21.01 (1.63, 40.39)*	12.51 (–6.95, 31.97)	8.33 (–25.23, 41.88)	Taiji (29.8%)			
20.69 (1.62, 39.75)*	12.19 (–14.53, 38.91)	8.01 (–11.67, 27.69)	–0.32 (–27.51, 26.87)	Usual care (29.8%)		
22.31 (9.12, 35.49)*	13.81 (0.51, 27.11)*	9.63 (–20.77, 40.03)	1.30 (–12.91, 15.51)	1.62 (–21.56, 24.80)	Usual rehabilitation (23.1%)	
**Walking ability (SMD (95% CI))**						
Baduanjin (96.2%)						
0.50 (–0.26, 1.26)	Taiji (67.7%)					
1.04 (0.45, 1.63)*	0.54 (0.06, 1.01)*	Usual care (22.9%)				
0.93 (0.02, 1.84)*	0.43 (–0.09, 0.95)	–0.11 (–0.80, 0.59)	Usual rehabilitation (13.2%)			
**Balance ability (SMD (95% CI))**						
Wuqinxi (82.2%)						
0.20 (–1.05, 1.45)	Baduanjin (76.8%)					
0.33 (–0.88, 1.54)	0.13 (–0.76, 1.02)	Liuzijue (68.5%)				
0.45 (–0.67, 1.57)	0.25 (–0.47, 0.96)	0.12 (–0.57, 0.81)	Taiji (60.4%)			
1.02 (–0.62, 2.65)	0.81 (–0.50, 2.13)	0.68 (–0.69, 2.06)	0.57 (–0.65, 1.78)	Daoyin (28.5%)		
1.11 (–0.13, 2.35)	0.91 (0.14, 1.68)*	0.78 (–0.10, 1.65)	0.66 (0.07, 1.25)*	0.09 (–0.97, 1.16)	Usual care (17.4%)	
1.10 (0.04, 2.16)*	0.90 (0.23, 1.57)*	0.77 (0.18, 1.36)*	0.65 (0.29, 1.01)*	0.08 (–1.16, 1.33)	–0.01 (–0.66, 0.64)	Usual rehabilitation (16.2%)
**Self-care ability (SMD (95% CI))**						
Baduanjin (68.9%)						
–0.19 (–25.07, 24.68)	Daoyin (62.2%)					
1.70 (–8.11, 11.51)	1.89 (–20.97, 24.75)	Taiji (58.5%)				
2.08 (–8.69, 12.86)	2.28 (–23.13, 27.69)	0.39 (–10.72, 11.50)	Liuzijue (55.9%)			
4.81 (–13.44, 23.05)	5.00 (–11.91, 21.91)	3.11 (–12.27, 18.49)	2.72 (–16.25, 21.69)	Usual care (39.9%)		
8.08 (1.42, 14.75)*	8.28 (–15.69, 32.24)	6.39 (–0.81, 13.59)	6.00 (–2.46, 14.45)	3.28 (–13.71, 20.26)	Usual rehabilitation (14.7%)	
**Cognitive functioning (MD (95% CI))**						
Baduanjin (97.0%)						
0.62 (–0.10, 1.33)	Liuzijue (65.2%)					
0.68 (–0.24, 1.60)	0.06 (–0.71, 0.84)	Taiji (60.1%)				
1.15 (0.54, 1.77)*	0.53 (0.17, 0.90)*	0.47 (–0.21, 1.16)	Usual care (26.3%)			
1.82 (1.38, 2.26)*	1.21 (0.37, 2.04)*	1.14 (0.13, 2.16)*	0.67 (–0.08, 1.43)	Usual rehabilitation (1.4%)		
**Depression (SMD (95% CI))**						
Liuzijue (82.8%)						
–0.87 (–5.08, 3.34)	Baduanjin (63.6%)					
–1.21 (–4.74, 2.32)	–0.34 (–4.34, 3.67)	Yijinjing (58.1%)				
–1.46 (–4.27, 1.34)	–0.59 (–4.48, 3.29)	–0.25 (–3.39, 2.88)	Taiji (53.5%)			
–1.39 (–5.67, 2.89)	–0.52 (–5.82, 4.77)	–0.18 (–4.96, 4.59)	0.07 (–3.70, 3.84)	Daoyin (53.4%)		
–2.30 (–4.96, 0.36)	–1.43 (–4.69, 1.84)	–1.09 (–3.41, 1.23)	–0.84 (–2.95, 1.28)	–0.91 (–5.08, 3.27)	Usual rehabilitation (30.2%)	
–3.51 (–6.22, –0.80)*	–2.64 (–6.77, 1.50)	–2.30 (–5.74, 1.14)	–2.04 (–3.84, –0.25)*	–2.11 (–5.42, 1.20)	–1.21 (–3.75, 1.33)	Usual care (8.4%)
**Quality of life (SMD (95% CI))**						
Baduanjin (86.5%)						
0.22 (–2.34, 2.79)	Wuqinxi (73.4%)					
1.04 (–0.40, 2.48)	0.82 (–1.74, 3.38)	Taiji (51.7%)				
1.52 (0.20, 2.84)*	1.30 (–1.33, 3.93)	0.48 (–0.54, 1.49)	Usual care (26.2%)			
1.88 (0.57, 3.20)*	1.66 (–0.54, 3.86)	0.84 (–0.47, 2.15)	0.36 (–1.07, 1.80)	Usual rehabilitation (12.2%)		
**Quality of sleep (SMD (95% CI))**						
Baduanjin (96.4%)						
–3.00 (–6.74, 0.74)	Daoyin (56.6%)					
–3.16 (–7.64, 1.31)	–0.16 (–4.28, 3.96)	Wuqinxi (54.9%)				
–3.32 (–6.56, –0.08)*	–0.32 (–3.05, 2.41)	–0.16 (–3.25, 2.93)	Taiji (51.8%)			
–3.90 (–6.81, –0.98)*	–0.90 (–3.24, 1.44)	–0.74 (–4.13, 2.66)	–0.58 (–1.99, 0.83)	Usual care (31.1%)		
–5.16 (–9.46, –0.86)*	–2.16 (–6.09, 1.77)	–2.00 (–3.23, –0.77)*	–1.84 (–4.67, 0.99)	–1.26 (–4.43, 1.90)	Usual rehabilitation (9.2%)	

Each intervention modality is represented by the name of the intervention (SUCRA values); cells show the results of pairwise comparisons between any two intervention modalities; bolded with “*” indicates *p* < 0.05; green indicates high certainty of evidence, blue indicates moderate certainty of evidence, yellow indicates low certainty of evidence, and red shows very low certainty of evidence. MD, mean difference; SMD, standardized mean difference; CI, confidence interval.

#### 3.5.2 Lower Limb Motor Ability

In terms of improving lower limb motor ability, pairwise comparison results indicated that Taiji (MD = 3.94, 95% CI = 1.90, 5.98, *p* < 0.05) and Baduanjin (MD = 2.56, 95% CI = 0.33, 4.78, *p* < 0.05) were more effective than conventional rehabilitation. Additionally, Taiji (MD = 6.00, 95% CI = 1.35, 10.66, *p* < 0.05) and Baduanjin (MD = 4.62, 95% CI = 0.75, 8.49, *p* < 0.05) were more effective than routine care. In the remaining comparisons, statistically significant differences were not observed (*p* > 0.05). The SUCRA values for lower limb motor ability ranked interventions in the following descending order: Taiji (SUCRA = 91.8%), Baduanjin (SUCRA = 70.7%), Daoyin (SUCRA = 50.6%), conventional rehabilitation (SUCRA = 30.0%), and routine care (SUCRA = 6.8%) (see Table 2).

#### 3.5.3 Overall Motor Ability

In terms of improving overall motor ability, pairwise comparison results indicated that Baduanjin was more effective than Taiji (MD = 21.01, 95% CI = 1.63, 40.39, *p* < 0.05), routine care (MD = 20.69, 95% CI = 1.62, 39.75, *p* < 0.05), and conventional rehabilitation (MD = 22.31, 95% CI = 9.12, 35.49, *p* < 0.05). Daoyin demonstrated greater effectiveness than traditional rehabilitation (MD = 13.81, 95% CI: 0.51, 27.11, *p* < 0.05). In the other comparisons, statistically significant differences were not found (*p* > 0.05). The SUCRA values for overall motor ability ranked interventions in the following descending order: Baduanjin (SUCRA = 91.9%), Taiji (SUCRA = 69.9%), Daoyin (SUCRA = 55.5%), Liuzijue (SUCRA = 29.8%), routine care (SUCRA = 29.8%), and conventional rehabilitation (SUCRA = 23.1%) (see Table 2).

#### 3.5.4 Walking Ability

In terms of improving walking ability, pairwise comparison results showed that Baduanjin (SMD = 1.04, 95% CI = 0.45, 1.63, *p* < 0.05) and Taiji (SMD = 0.54, 95% CI = 0.06, 1.01, *p* < 0.05) were more effective than routine care. Additionally, Baduanjin (SMD = 0.93, 95% CI = 0.02, 1.84, *p* < 0.05) was more effective than conventional rehabilitation. No statistically significant differences were observed in the other comparisons (*p* > 0.05). The SUCRA values for walking ability ranked interventions in the following descending order: Baduanjin (SUCRA = 96.2%), Taiji (SUCRA = 67.7%), routine care (SUCRA = 22.9%), and conventional rehabilitation (SUCRA = 13.2%) (see Table 2).

#### 3.5.5 Balance Ability

In terms of improving balance ability, pairwise comparison results showed that Baduanjin (SMD = 0.91, 95% CI = 0.14, 1.68, *p* < 0.05) and Taiji (SMD = 0.66, 95% CI = 0.07, 1.25, *p* < 0.05) were more effective than routine care. Wuqinxi (SMD = 1.10, 95% CI = 0.04, 2.16, *p* < 0.05), Baduanjin (SMD = 0.90, 95% CI = 0.23, 1.57, *p* < 0.05), Liuzijue (SMD = 0.77, 95% CI = 0.18, 1.36, *p* < 0.05), and Taiji (SMD = 0.65, 95% CI = 0.29, 1.01, *p* < 0.05) were all more effective than conventional rehabilitation. However, further validation against specific clinical thresholds, such as a 5- to 8-point increase on the Berg Balance Scale (associated with reduced fall risk), is needed to confirm clinical applicability. In the other comparisons, no significant differences were detected (*p* > 0.05). The SUCRA values for improving balance ability ranked interventions in the following descending order: Wuqinxi (SUCRA = 82.2%), Baduanjin (SUCRA = 76.8%), Liuzijue (SUCRA = 68.5%), Taiji (SUCRA = 60.4%), Daoyin (SUCRA = 28.5%), routine care (SUCRA = 17.4%), and conventional rehabilitation (SUCRA = 16.2%) (see Table 2).

#### 3.5.6 Self-Care Ability 

Regarding improving self-care ability, the pairwise comparison results indicated that Baduanjin was more effective than conventional rehabilitation (MD = 8.08, 95% CI = 1.42, 14.75, *p* < 0.05). Moreover, no significant differences were identified in the other comparisons (*p* > 0.05). The SUCRA values for improving self-care ability ranked interventions in the following descending order: Baduanjin (SUCRA = 68.9%), Daoyin (SUCRA = 62.2%), Taiji (SUCRA = 58.5%), Liuzijue (SUCRA = 55.9%), routine care (SUCRA = 39.9%), and conventional rehabilitation (SUCRA = 14.7%) (see Table 2).

#### 3.5.7 Cognitive Functioning

In terms of cognitive function improvement, Baduanjin (SMD = 1.15, 95% CI = 0.54, 1.77, *p* < 0.05) and Liuzijue (SMD = 0.53, 95% CI = 0.17, 0.90, *p* < 0.05) were more effective than routine care. Additionally, Baduanjin (SMD = 1.82, 95% CI = 1.38, 2.26, *p* < 0.05), Liuzijue (SMD = 1.21, 95% CI = 0.37, 2.04, *p* < 0.05), and Taiji (SMD = 1.14, 95% CI = 0.13, 2.16, *p* < 0.05) were more effective than conventional rehabilitation. The SUCRA ranking results demonstrated that Baduanjin was the most effective intervention for enhancing cognitive function (SUCRA = 97.0%), followed by Liuzijue (SUCRA = 65.2%), Taiji (SUCRA = 60.1%), routine care (SUCRA = 26.3%), and conventional rehabilitation (SUCRA = 1.4%) (see Table 2).

#### 3.5.8 Depression

In terms of improving depression, pairwise comparison results indicated that Liuzijue (SMD = –3.51, 95% CI = –6.22, –0.80, *p* < 0.05) and Taiji (SMD = –2.04, 95% CI = –3.84, –0.25, *p* < 0.05) were more effective than routine care. No significant differences were identified in the other comparisons (*p* > 0.05). The SUCRA ranking results showed that Liuzijue was the most effective intervention for improving depression (SUCRA = 82.8%), followed by Baduanjin (SUCRA = 63.6%), Yijinjing (SUCRA = 58.1%), Taiji (SUCRA = 53.5%), Daoyin (SUCRA = 53.4%), conventional rehabilitation (SUCRA = 30.2%), and routine care (SUCRA = 8.4%) (see Table 2).

#### 3.5.9 Quality of Life

In terms of improving quality of life, Baduanjin was more effective than routine care (SMD = 1.52, 95% CI = 0.20, 2.84, *p* < 0.05) and conventional rehabilitation (SMD = 1.88, 95% CI = 0.57, 3.20, *p* < 0.05). The SUCRA ranking results showed that Baduanjin was the most effective intervention for improving quality of life (SUCRA = 86.5%), followed by Wuqinxi (SUCRA = 73.4%), Taiji (SUCRA = 51.7%), routine care (SUCRA = 26.2%), and conventional rehabilitation (SUCRA = 12.2%) (see Table 2).

#### 3.5.10 Quality of Sleep

In terms of improving sleep quality, Baduanjin was more effective than Taiji (MD = –3.32, 95% CI = –6.56, –0.08, *p* < 0.05), routine care (MD = –3.90, 95% CI = –6.81, –0.98, *p* < 0.05), and conventional rehabilitation (MD = –5.16, 95% CI = –9.46, –0.86, *p* < 0.05). Wuqinxi (MD = –2.00, 95% CI = –3.23, –0.77, *p* < 0.05) was also more effective than conventional rehabilitation. Meanwhile, no significant differences were identified in the other comparisons (*p* > 0.05). The SUCRA values for improving sleep quality ranked interventions in the following descending order: Baduanjin (SUCRA = 96.4%), Daoyin (SUCRA = 56.6%), Wuqinxi (SUCRA = 54.9%), Taiji (SUCRA = 51.8%), routine care (SUCRA = 31.1%), and conventional rehabilitation (SUCRA = 9.2%) (see Table 2).

### 3.6 Comprehensive SUCRA Ranking Results

By summarizing the SUCRA rankings across all indicators, Baduanjin ranked first in seven out of ten and second in three. Overall, this suggests that Baduanjin may be the most comprehensive and effective intervention for improving motor and non-motor symptoms in stroke patients; the detailed results are presented in Table 3.

**Table 3.** **Comprehensive SUCRA ranking results**.

	Baduanjin	Taiji	Daoyin	Liuzijue	Wuqinxi	Yijinjing	Usual care	Usual rehabilitation
Upper limb motor ability	1	2	3	-	-	-	4	5
Lower limb motor ability	2	1	3	-	-	-	5	4
Overall motor ability	1	2	3	4	-	-	5	6
Walking ability	1	2	-	-	-	-	3	4
Balance ability	2	4	5	3	1	-	6	7
Self-care ability	1	3	2	4	-	-	5	6
Cognitive functioning	1	3		2	-	-	4	5
Depression	2	4	5	1	-	3	7	6
Quality of life	1	3	-	-	2	-	4	5
Quality of sleep	1	4	2	-	3	-	5	6

The ranking of each intervention modality in relation to each indicator.

### 3.7 Transitivity, Publication Bias, Heterogeneity, and Inconsistency

A sensitivity analysis was conducted after excluding one study with a high risk of bias, and the results remained unchanged, indicating the stability of the findings (**Supplementary Table 12**). The adjusted comparison funnel plots for all indicators were generally symmetrical, suggesting no significant publication bias in this study (**Supplementary Figs. 11–15**). By comparing the statistical significance of actual intervals with predicted intervals, results showed that most indicators had high heterogeneity (**Supplementary Table 15**). Inconsistency testing revealed minor inconsistency in lower limb motor ability (*p* < 0.05), which was downgraded in the CINeMA evaluation, while no inconsistency was found in the other indicators (*p* > 0.05) (**Supplementary Tables 13,14**). Box plots considering seven effect modifiers (sample size, age, disease duration, gender, intervention duration, intervention frequency, and intervention time) showed that the effect modifiers for each intervention largely overlapped, indicating no significant transitivity issues in this study (**Supplementary Figs. 16–22**).

### 3.8 Certainty of Evidence Assessment

The certainty of the evidence was assessed using CINeMA. Out of the 133 comparisons, 7 (5.3%) were rated as high certainty of evidence, 13 (9.8%) as moderate certainty of evidence, 63 (47.4%) were classified as low certainty of evidence, and 48 (36.1%) as very low certainty of evidence. The primary reasons for downgrading the certainty of evidence were within-study bias, imprecision, and heterogeneity (**Supplementary Tables 2–11**).

## 4. Discussion

Based on a network meta-analysis, this study incorporates 43 studies to assess the effects of traditional Chinese exercises on stroke rehabilitation. The results indicate that different types of exercises exhibit significant variations in effectiveness across specific functional domains. Baduanjin was particularly beneficial in improving upper limb motor ability, overall motor ability, walking ability, self-care ability, cognitive function, quality of life, and sleep quality in stroke patients. Taiji was more effective in enhancing lower limb motor ability, Wuqinxi showed superiority in improving balance, and Liuzijue was more effective in alleviating depression. The SUCRA ranking results across all 10 indicators revealed that Baduanjin ranked first in seven and second in three. Therefore, Baduanjin is suggested to be the most comprehensive and effective traditional Chinese exercise intervention for improving symptoms in stroke patients. The limb motor impairments in stroke patients are primarily caused by damage to the neural networks in the brain, which leads to disrupted neural signal transmission, muscle atrophy, and abnormal muscle tone, affecting bodily control and coordination [12, 79, 80]. These impairments often manifest as hemiplegia, limb weakness, muscle spasms, reduced coordination, and ataxia [81]. In terms of improving motor function, Baduanjin demonstrated the most significant effects on upper limb motor ability, overall motor ability, and walking ability, outperforming other traditional Chinese exercises, conventional rehabilitation, and routine care. Previous studies have shown that Baduanjin positively affects motor function and walking in stroke patients [42, 82], which is consistent with the findings of this study. These health benefits may be attributed to the long-term practice of Baduanjin, promoting blood circulation in the limbs and lower back and enhancing neural transmission in the central nervous system [83]. Additionally, Baduanjin can upregulate neurotrophic factors such as brain-derived neurotrophic factor (BDNF) and vascular endothelial growth factor (VEGF), promoting neurogenesis and migration in the central nervous system [84]. The coordinated movements and breathing techniques in Baduanjin facilitate sensory–motor integration and improve proprioceptive feedback, both of which are fundamental for motor learning.

However, Taiji was the most effective for lower limb motor ability. Compared to other traditional Chinese exercises, Taiji emphasizes flexible transitions in footwork, with smooth shifts between relaxed and controlled movements in the hip, knee, and ankle joints, effectively enhancing lower limb muscle strength, endurance, and stability [85]. Other traditional Chinese exercises tend to involve a smaller range of lower limb movements, which may explain why Taiji is superior to Qigong in improving lower limb function in stroke patients. The potential physiological mechanism may involve Taiji strengthening neurons in motor and attentional control regions, enhancing muscle strength, coordination, and control [54], thereby improving lower limb function [86]. Electromyography studies have confirmed that a year of Taiji exercise therapy can improve neuromuscular responses in the lower limbs of stroke patients [87]. Wuqinxi showed the best results regarding balance improvement, followed by Baduanjin and Liuzijue; meanwhile, Wuqinxi integrates guided movements and breathing techniques, combining external movements with internal calmness [88]. The superior effect of Wuqinxi on balance may be due to the series of alternating single-leg movements required during practice, which positively influence balance control in both directions [77]. Research has confirmed that Wuqinxi can improve BBS (Berg Balance Scale) and TIS (Trunk Impairment Scale) scores, reduce the mean sway path of the center of gravity during double- and single-leg standing, and improve postural stability and trunk control, leading to enhanced balance function [89]. Additionally, after a stroke, patients experience a decline in bilateral trunk muscle strength, proprioception, coordination, and delayed movement responses [90, 91, 92]. Wuqinxi combines spinal movement with whole-body motion, increasing muscle strength by stretching the spine in various directions, stimulating length and tension receptors, enhancing proprioceptive input, and activating trunk muscles. Combining diaphragmatic breathing with limb movements also strengthens the inspiratory muscles, improving spinal stability [93, 94] and enhancing balance post-stroke [95, 96]. Although the ranking results indicate that Wuqinxi is the most effective intervention for improving balance in stroke patients, this finding should be interpreted cautiously since only one study explored the effects of Wuqinxi on balance in stroke patients. Thus, further high-quality, large-scale RCTs are needed to validate this conclusion.

Traditional exercise interventions have demonstrated varying levels of effectiveness in improving the self-care ability and mental health of stroke patients. Baduanjin, in particular, excelled in enhancing self-care ability, with other exercises such as Taiji and Liuzijue also showing positive effects. Stroke patients often experience difficulties with daily activities such as washing, dressing, eating, and walking [97, 98]. Previous studies have demonstrated that Baduanjin enhances physical strength and flexibility and promotes brain neuroplasticity, thereby improving the abilities of patients to perform daily activities [60, 61].

Baduanjin showed the best results regarding cognitive function improvement, significantly outperforming Liuzijue and Taiji. From the perspective of using traditional Chinese medicine (TCM), Baduanjin promotes the smooth flow of meridian systems, regulates the balance of yin and yang, and nourishes the kidneys and brain while also maintaining the normal functioning of the brain and kidneys through the harmonization of heart and kidney functions [99]. Additionally, Baduanjin enhances synaptic plasticity vascular function and reduces risk factors associated with cognitive impairment, such as blood lipids, glucose, and blood pressure [100, 101], making it particularly effective in improving cognitive function in stroke patients. In terms of alleviating depressive symptoms, Liuzijue demonstrated the most significant effects, followed by Baduanjin, Yijinjing, and Taiji. Liuzijue, a form of qigong that combines breathing with vocalization, stands out among other qigong exercises due to its unique features. In the theory associated with TCM, emotions correspond to the five elements, and persistent negative emotions are detrimental to health. The “he” sound in Liuzijue, related to the element of fire, corresponds to the musical note “zhǐ”, which has a bright and uplifting tone, thus creating a sense of euphoria in patients following a practice. Therefore, by regulating breathing rhythms and sound resonance, Liuzijue helps relax the body and mind, alleviating the tension and anxiety caused by physical impairments. Previous studies have suggested that Liuzijue may be the most effective traditional exercise intervention for alleviating depressive symptoms in breast cancer survivors [102], aligning with the findings of the present study.

Baduanjin has been proven to be the most effective intervention in improving quality of life. Several studies have shown that physical and mental health scores significantly improve following Baduanjin intervention [55, 59, 61, 62]. In a six-month Baduanjin exercise program for breast cancer survivors, significant improvements were observed in heart rate variability, range of motion in the affected shoulder joint, depression levels, and quality of life [103]. Wuqinxi and Taiji also showed positive effects on quality of life, with Wuqinxi demonstrating a particularly high level of effectiveness.

Regarding sleep quality improvement, Baduanjin again outperformed the other traditional exercises and proved the most effective, followed by Daoyin, Wuqinxi, and Taiji. A study showed that after 12 weeks of exercise intervention, Baduanjin significantly improved sleep quality, latency, duration, disturbances, and daytime dysfunction in stroke patients compared to conventional exercises [56]. The potential reasons for the effectiveness of Baduanjin in improving sleep quality may be attributed to its emphasis on concentration, mindfulness, and breathing regulation, which promote relaxation, reduce stress, and enhance sleep depth and quality [104].

Our research findings suggest that traditional Chinese exercises such as Taiji, Baduanjin, Wuqinxi, Liuzijue, and Yijinjing are all effective therapeutic exercises that warrant broader promotion and application. However, their impacts may differ based on the specific outcome measures evaluated. Therefore, future studies could explore the underlying therapeutic mechanisms of these exercise methods in greater depth and offer stronger scientific evidence to validate their effectiveness; a cautious interpretation of our findings is essential.

The SUCRA values provide a quantitative metric to rank interventions across multiple outcomes, offering insight into their relative efficacy [105]. However, interpreting these rankings within the context of clinical thresholds is essential to ensure their practical relevance. For instance, a high SUCRA value for an intervention, such as Baduanjin in improving cognitive function (SUCRA = 97.0%), indicates superior efficacy relative to other interventions. However, the practical application depends on whether the improvement surpasses clinically meaningful benchmarks, such as established changes in Fugl-Meyer Assessment or Montreal Cognitive Assessment scores. For clinical translation, improvements should be evaluated against thresholds that signify functional recovery or patient quality-of-life enhancement. For example, an improvement in walking ability measured by the Timed Up and Go (TUG) test should exceed 3–5 seconds to be considered clinically significant [106]. Similarly, a Berg Balance Scale increase of 8–10 points in balance ability is generally regarded clinically meaningful in reducing fall risk [107]. By mapping SUCRA values to these benchmarks, clinicians can prioritize interventions based on statistical rankings and their real-world impact. For example, although Wuqinxi ranked first for balance improvement, its clinical significance requires validation against postural stability and fall prevention benchmarks to establish practical utility. Future studies should aim to integrate SUCRA rankings with established clinical guidelines, ensuring that findings are actionable and aligned with patient-centered outcomes.

The demographic and clinical profiles of the populations included in this study provide important context for interpreting the findings. The average age of participants across the included studies was approximately 59.98 years, ranging from middle-aged to older adults. This distribution reflects a key demographic commonly affected by stroke, aligning with global trends [108]. However, the relatively low representation of older adults (>75 years) and the exclusion of younger stroke patients (<18 years) may limit the applicability of these findings to age groups outside this range. Clinically, the majority of studies involved patients with ischemic strokes, which represent the majority of global stroke cases [1]; however, hemorrhagic strokes were underrepresented. Stroke severity also varied, but the lack of standardized reporting on severity measures (e.g., NIH Stroke Scale) across studies limits the assessment of outcomes across the spectrum of stroke severity. Furthermore, most interventions were conducted on patients in the subacute or chronic phases of recovery, making it difficult to extrapolate these findings to the acute phase of stroke rehabilitation. Geographically, the included studies were predominantly conducted in Asia, with a large majority from China. This regional concentration may influence the generalizability of results to non-Asian populations due to cultural, dietary, and healthcare system differences. For instance, traditional Chinese exercises such as Baduanjin, Taiji, and Wuqinxi are culturally ingrained in Asia, where they are more familiar and widely accepted; thus, their implementation and adherence might differ significantly in Western or other non-Asian contexts, potentially affecting efficacy. Therefore, to enhance generalizability, future studies should include more diverse populations, encompassing broader age ranges, balanced stroke types, and varying levels of stroke severity. Additionally, conducting trials in non-Asian countries could provide insights into the feasibility and cultural adaptability of traditional Chinese exercises in different healthcare and social settings.

The effectiveness and feasibility of traditional Chinese exercises, such as Baduanjin and Taiji, may vary in non-Chinese populations because of limited familiarity with these practices and restricted access to qualified instructors. Cultural differences in attitudes toward mindfulness and body-centered exercises can also influence acceptance and adherence. To address these challenges, simplified versions of the exercises, tailored educational campaigns, and training programs for local healthcare providers could facilitate adoption. Additionally, incorporating these practices into existing rehabilitation programs and using digital platforms for instruction could improve accessibility and engagement, ensuring broader applicability in diverse cultural contexts.

This study provides practical guidance for clinicians in selecting traditional Chinese exercises for stroke rehabilitation based on specific patient conditions. For example, Baduanjin is recommended for comprehensive motor recovery and improvements in quality of life and sleep, while Taiji is particularly effective for enhancing lower limb motor ability and balance. Wuqinxi shows promise for addressing postural instability, and Liuzijue is especially beneficial for alleviating depressive symptoms. These exercises may also be combined with other rehabilitation methods, such as conventional physical therapy or neurostimulation, to optimize patient outcomes. However, several limitations must be acknowledged, including the relatively small sample sizes of some included studies, heterogeneity in intervention protocols, and the geographic concentration of studies in Asia, which may limit the generalizability of the results. Future research should focus on conducting larger, multicenter RCTs, developing standardized protocols, exploring combination therapies, and investigating the underlying mechanisms to provide a stronger evidence base for integrating traditional Chinese exercises into clinical practice. Long-term follow-up studies are also needed to evaluate the long-term sustainability of the benefits of these interventions.

## 5. Conclusions

This study found that Baduanjin demonstrated significant advantages in improving upper limb motor ability, overall motor ability, self-care ability, walking ability, cognitive function, sleep quality, and quality of life, highlighting its potential as a comprehensive rehabilitation intervention. Taiji enhanced lower limb motor ability, while Wuqinxi showed unique advantages in improving balance, and Liuzijue was the most effective in alleviating depressive symptoms.

## Availability of Data and Materials

All additional data are available in the supplementary information files. For further information, please contact the corresponding author.

## Supplementary Material

Supplementary material associated with this article can be found, in the online version, at https://doi.org/10.31083/RCM27104.
