# Supplementary material for: Network Meta-Analysis on the Effects of Traditional Chinese Exercise on Stroke Patients
Source: Rev Cardiovasc Med. 2025 Mar 24;26(3):27104. doi: 10.31083/RCM27104 (PMC11951498; doi:10.31083/RCM27104)
Supplement: Supplementary file 1 [file 2153-8174-26-3-27104-s1.zip › Supplementary Material-V1.docx]

***Supplementary Material***

[1 Search strategy 1](#_Toc1630595319)

[Table S1 Search strategy 1](#_Toc1376707066)

[2 Certainty of evidence 6](#_Toc1326845484)

[Table S2 CINeMA assessment for Upper limb motor ability 6](#_Toc821859140)

[Table S3 CINeMA assessment for Lower limb motor ability 7](#_Toc371748476)

[Table S4 CINeMA assessment for Overall motor ability 8](#_Toc946707009)

[Table S5 CINeMA assessment for Walking ability 9](#_Toc598359640)

[Table S6 CINeMA assessment for Balance ability 10](#_Toc2112034226)

[Table S7 CINeMA assessment for Self-care ability 11](#_Toc1202035119)

[Table S8 CINeMA assessment for Cognitive functioning 12](#_Toc1225577704)

[Table S9 CINeMA assessment for Depression 13](#_Toc1768812751)

[Table S10 CINeMA assessment for Quality of life 14](#_Toc819780636)

[Table S11 CINeMA assessment for Quality of sleep 15](#_Toc1945553747)

[3 Network evidence Plot 16](#_Toc1335816607)

[Figure S1 Network evidence Plot for Upper limb motor ability 16](#_Toc1275668111)

[Figure S2 Network evidence Plot for Lower limb motor ability 16](#_Toc1824693576)

[Figure S3 Network evidence Plot for Overall motor ability 17](#_Toc1558452672)

[Figure S4 Network evidence Plot for Walking ability 17](#_Toc56015845)

[Figure S5 Network evidence Plot for Balance ability 18](#_Toc860469529)

[Figure S6 Network evidence Plot for Self-care ability 18](#_Toc756495005)

[Figure S7 Network evidence Plot for Cognitive functioning 19](#_Toc1308358795)

[Figure S8 Network evidence Plot for Depression 19](#_Toc1308358795)

[Figure S9 Network evidence Plot for Quality of life 20](#_Toc1501205932)

[Figure S10 Network evidence Plot for Quality of sleep 20](#_Toc2130214168)

[4 Corrected Comparison Funnel Plot 21](#_Toc1809642439)

[Figure S11 Corrected Comparison Funnel Plot for Walking Ability 21](#_Toc1997063459)

[Figure S12 Corrected Comparison Funnel Plot for Balance Ability 21](#_Toc1623636450)

[Figure S13 Corrected Comparison Funnel Plot for Self-care ability 22](#_Toc383112721)

[Figure S14 Corrected Comparison Funnel Plot for Depression 22](#_Toc819528141)

[Figure S15 Corrected Comparison Funnel Plot for Quality of life 23](#_Toc1996837576)

[5 Sensitivity analysis 24](#_Toc2122188163)

[Table S12 Results of Sensitivity analysis 24](#_Toc60562518)

[6 Global inconsistency test 26](#_Toc2114474995)

[Table S13 Global inconsistency test 26](#_Toc1421850409)

[7 Local inconsistency test 2](#_Toc1989283894)8

[Table S14 Local inconsistency test 2](#_Toc1868989962)8

[8 Heterogeneity test 32](#_Toc870986665)

[Table S15 heterogeneity test 32](#_Toc1424340703)

[9 Transitivity test 45](#_Toc893982212)

[Figure S16 Transitivity test (Box Plot) for Sample size(n) 45](#_Toc1363442672)

[Figure S17 Transitivity test (Box Plot) for age(year) 45](#_Toc1730474814)

[Figure S18 Transitivity test (Box Plot) for Disease duration(w) 46](#_Toc719167577)

[Figure S19 Transitivity test (Box Plot) for Gender( female%) 46](#_Toc1011501323)

[Figure S20 Transitivity test (Box Plot) for Duration of intervention(w) 47](#_Toc822186009)

[Figure S21 Transitivity test (Box Plot) for Intervention intensity(times/w) 47](#_Toc1570468465)

[Figure S22 Transitivity test (Box Plot) for Intervention period(min/times) 48](#_Toc141985978)

[10 Reference list of included studies 49](#_Toc504000429)

1. Search strategy

| Table S1 Search strategy | | |
| --- | --- | --- |
| **Pubmed** | | |
| #1 | (((((((((((((((((((((((((((((((((((((((("Tai Ji"[Mesh]) OR ("Martial Arts"[Mesh])) OR ("Qigong"[Mesh])) OR (Baduanjin[Title/Abstract])) OR (Chinese traditional exercises[Title/Abstract])) OR (Eight Pieces of Brocade[Title/Abstract])) OR (Eight Silk Weaving[Title/Abstract])) OR (Eight Silken Movements[Title/Abstract])) OR (Eight-Section Brocade[Title/Abstract])) OR (Eight-sectioned exercise[Title/Abstract])) OR (Five animal exercises[Title/Abstract])) OR (Five fowl exercises[Title/Abstract])) OR (Five poultry exercises[Title/Abstract])) OR (Gongfu[Title/Abstract])) OR (Health cultivation exercise[Title/Abstract])) OR (Kungfu[Title/Abstract])) OR (Liuzijue[Title/Abstract])) OR (Martial arts[Title/Abstract])) OR (Mind-body exercise[Title/Abstract])) OR (Qigong[Title/Abstract])) OR (Tai chi[Title/Abstract])) OR (Taichichuan[Title/Abstract])) OR (Taiji[Title/Abstract])) OR (The six letter word[Title/Abstract])) OR (Traditional exercises[Title/Abstract])) OR (Traditional chinese exercises[Title/Abstract])) OR (Traditional exercises[Title/Abstract])) OR (Wuqinxi[Title/Abstract])) OR (Wushu[Title/Abstract])) OR (YiJinjing[Title/Abstract])) OR (Twelve pieces of brocade[Title/Abstract])) OR (Twelve Chapter Brocade[Title/Abstract])) OR (Twelve ectioned Exercise[Title/Abstract])) OR (12-Step Daoyin Health Preservation Exercises[Title/Abstract])) OR (12-Step guided health exercises[Title/Abstract])) OR (Guide health twelve exercises[Title/Abstract])) OR (Daoyin[Title/Abstract])) OR (mingmu gong[Title/Abstract])) OR (Dawu[Title/Abstract])) OR (Daoyin shu[Title/Abstract])) OR (guiding technique[Title/Abstract]) | 345,264 |
| #2 | (((((((((((((((((((("Stroke"[Mesh]) OR ("Brain Ischemia"[Mesh])) OR (Acute ischemic stroke[Title/Abstract])) OR (Apoplexy[Title/Abstract])) OR (Brain Vascular Accident[Title/Abstract])) OR (Cerebral apoplexy[Title/Abstract])) OR (Cerebral arterial thrombosis[Title/Abstract])) OR (Cerebral disease[Title/Abstract])) OR (Cerebral infarction[Title/Abstract])) OR (Cerebral ischemia[Title/Abstract])) OR (Cerebral Stroke[Title/Abstract])) OR (Cerebrovascular Accident[Title/Abstract])) OR (Cerebrovascular Apoplexy[Title/Abstract])) OR (Cerebrovascular disease[Title/Abstract])) OR (Cerebrovascular Stroke[Title/Abstract])) OR (CVA[Title/Abstract])) OR (Encephalorrhagia[Title/Abstract])) OR (Hemorrhagic stroke[Title/Abstract])) OR (Infarction of brain[Title/Abstract])) OR (Ischemic stroke[Title/Abstract])) OR (Stroke[Title/Abstract]) | 447901 |
| #3 | ((((((((((((("Clinical Trial" [Publication Type]) OR ("Clinical Trials as Topic"[Mesh])) OR ("Random Allocation"[MeSH])) OR ("Double-Blind Method"[MeSH])) OR ("single-blind method"[MeSH])) OR ("Control Groups"[MeSH])) OR ("cross-over studies"[MeSH])) OR (random*[Title/Abstract])) OR (trial[Title/Abstract])) OR (crossover[Title/Abstract])) OR (cross-over[Title/Abstract])) OR ("open-label"[Title/Abstract])) OR ("non-masked"[Title/Abstract])) OR (unblinded[Title/Abstract]) | 2,571,403 |
| #4 | #1 AND #2 AND #3 | 3050 |
| **Web of science** | | |
| #1 | TS=(Baduanjin) OR TS=(Chinese traditional exercises) OR TS=(Eight Pieces of Brocade) OR TS=(Eight Silk Weaving) OR TS=(Eight Silken Movements) OR TS=(Eight-Section Brocade) OR TS=(Eight-sectioned exercise) OR TS=(Five animal exercises) OR TS=(Five fowl exercises) OR TS=(Five poultry exercises) OR TS=(Gongfu) OR TS=(Health cultivation exercise) OR TS=(Kungfu) OR TS=(Liuzijue) OR TS=(Martial arts) OR TS=(Mind-body exercise) OR TS=(Qigong) OR TS=(Tai chi) OR TS=(Taichichuan) OR TS=(Taiji) OR TS=(The six letter word) OR TS=(Traditional exercises) OR TS=(Traditional chinese exercises) OR TS=(Traditional exercises) OR TS=(Wuqinxi) OR TS=(Wushu) OR TS=(YiJinjing) OR TS=(Twelve pieces of brocade) OR TS=(Twelve Chapter Brocade) OR TS=(Twelve ectioned Exercise) OR TS=(12-Step Daoyin Health Preservation Exercises) OR TS=(12-Step guided health exercises) OR TS=(Guide health twelve exercises) OR TS=(Daoyin) OR TS=(mingmu gong) OR TS=(Dawu) OR TS=(Daoyin shu) OR TS=(guiding technique) | 100949 |
| #2 | TS=(Acute ischemic stroke) OR TS=(Apoplexy) OR TS=(Brain Vascular Accident) OR TS=(Cerebral apoplexy) OR TS=(Cerebral arterial thrombosis) OR TS=(Cerebral disease) OR TS=(Cerebral infarction) OR TS=(Cerebral ischemia) OR TS=(Cerebral Stroke) OR TS=(Cerebrovascular Accident) OR TS=(Cerebrovascular Apoplexy) OR TS=(Cerebrovascular disease) OR TS=(Cerebrovascular Stroke) OR TS=(CVA) OR TS=(Encephalorrhagia) OR TS=(Hemorrhagic stroke) OR TS=(Infarction of brain) OR TS=(Ischemic stroke) OR TS=(Stroke) | 526339 |
| #3 | TS=(Clinical Trial ) OR TS=(Clinical Trials as Topic) OR TS=(Random Allocation) OR TS=(Double-Blind Method) OR TS=(single-blind method) OR TS=(Control Groups) OR TS=(cross-over studies) OR TS=(random*) OR TS=(trial) OR TS=(crossover) OR TS=(cross-over) OR TS=(open-label) OR TS=(non-masked) OR TS=(unblinded) | 3,915,711 |
| #4 | #1 AND #2 AND #3 | 784 |
| **Embase** | | |
| #1 | tai chi'/exp OR 'martial art'/exp OR 'qigong'/exp | 7679 |
| #2 | baduanjin:ab,ti OR 'chinese traditional exercises':ab,ti OR 'eight pieces of brocade':ab,ti OR 'eight silk weaving':ab,ti OR 'eight silken movements':ab,ti OR 'eight-section brocade':ab,ti OR 'eight-sectioned exercise':ab,ti OR 'five animal exercises':ab,ti OR 'five fowl exercises':ab,ti OR 'five poultry exercises':ab,ti OR gongfu:ab,ti OR 'health cultivation exercise':ab,ti OR 'kung fu':ab,ti OR liuzijue:ab,ti OR 'martial arts':ab,ti OR 'mind-body exercise':ab,ti OR qigong:ab,ti OR taichichuan:ab,ti OR 'tai chi':ab,ti OR 'the six letter word':ab,ti OR 'traditional exercises':ab,ti OR 'traditional chinese exercises':ab,ti OR 'traditional exercises':ab,ti OR wuqinxi:ab,ti OR wushu:ab,ti OR yijinjing:ab,ti OR 'twelve pieces of brocade':ab,ti OR 'twelve chapter brocade':ab,ti OR 'twelve ectioned exercise':ab,ti OR '12-step daoyin health preservation exercises':ab,ti OR '12-step guided health exercises':ab,ti OR 'guide health twelve exercises':ab,ti OR daoyin:ab,ti OR 'mingmu gong':ab,ti OR dawu:ab,ti OR 'daoyin shu':ab,ti OR 'guiding technique':ab,ti | 6235 |
| #3 | #1 AND #2 | 9595 |
| #4 | cerebrovascular accident'/exp OR 'brain ischemia'/exp | 609958 |
| #5 | acute ischemic stroke':ab,ti OR apoplexy:ab,ti OR 'cerebral apoplexy':ab,ti OR 'cerebral artery thrombosis':ab,ti OR 'cerebral disease':ab,ti OR 'cerebral infarction':ab,ti OR 'cerebral ischemia':ab,ti OR 'cerebral stroke':ab,ti OR 'cerebrovascular accident':ab,ti OR 'cerebrovascular apoplexy':ab,ti OR 'cerebrovascular disease':ab,ti OR 'cerebrovascular stroke':ab,ti OR cva:ab,ti OR encephalorrhagia:ab,ti OR 'brain hemorrhage':ab,ti OR 'infarction of brain':ab,ti OR 'ischemic stroke':ab,ti OR stroke:ab,ti | 583916 |
| #6 | #4 AND #5 | 783980 |
| #7 | clinical trial (topic)'/exp OR 'randomization'/exp OR 'double blind procedure'/exp OR 'single blind procedure'/exp OR 'control group'/exp OR 'crossover procedure'/exp | 976256 |
| #8 | 'randomized controlled trial':ab,ti OR trial:ab,ti OR 'cross over':ab,ti OR crossover:ab,ti OR 'open label':ab,ti OR 'non masked':ab,ti OR unblinded:ab,ti OR 'clinical trial':it | 1339812 |
| #9 | #7 OR #8 | 1980810 |
| #10 | #3 AND #6 AND #9 | 132 |
| **Cochrane library** | | |
| #1 | MeSH descriptor: [Tai Ji] explode all trees | 585 |
| #2 | MeSH descriptor: [Martial Arts] explode all trees | 802 |
| #3 | MeSH descriptor: [Qigong] explode all trees | 159 |
| #4 | (Baduanjin):ti,ab,kw OR (Chinese traditional exercises):ti,ab,kw OR (Eight Pieces of Brocade):ti,ab,kw OR (Eight Silk Weaving):ti,ab,kw OR (Eight Silken Movements):ti,ab,kw (Word variations have been searched) | 1047 |
| #5 | (Eight-Section Brocade):ti,ab,kw OR (Eight-sectioned exercise):ti,ab,kw OR (Five animal exercises):ti,ab,kw OR (Five fowl exercises):ti,ab,kw OR (Five poultry exercises):ti,ab,kw | 40 |
| #6 | (Gongfu):ti,ab,kw OR (Health cultivation exercise):ti,ab,kw OR (Kungfu):ti,ab,kw OR (Liuzijue):ti,ab,kw OR (Martial arts):ti,ab,kw | 476 |
| #7 | (Mind-body exercise):ti,ab,kw OR (Qigong):ti,ab,kw OR (Tai chi):ti,ab,kw OR (Taichichuan):ti,ab,kw OR (Taiji):ti,ab,kw | 2861 |
| #8 | (The six letter word):ti,ab,kw OR (Traditional exercises):ti,ab,kw OR (Traditional chinese exercises):ti,ab,kw OR (Traditional exercises):ti,ab,kw OR (Wuqinxi):ti,ab,kw | 1723 |
| #9 | (Wushu):ti,ab,kw OR (YiJinjing):ti,ab,kw OR (Twelve pieces of brocade):ti,ab,kw OR (Twelve Chapter Brocade):ti,ab,kw OR (Twelve ectioned Exercise):ti,ab,kw | 60 |
| #10 | (12 Step Daoyin Health Preservation Exercises):ti,ab,kw OR (12 Step guided health exercises):ti,ab,kw OR (Guide health twelve exercises):ti,ab,kw OR (Daoyin):ti,ab,kw OR (mingmu gong):ti,ab,kw | 76 |
| #11 | (Dawu):ti,ab,kw OR (Daoyin shu):ti,ab,kw OR (guiding technique):ti,ab,kw | 281 |
| #12 | #1 OR #2 OR #3 OR #4 OR #5 OR #6 OR #7 OR #8 OR #9 OR #10 OR #11 | 5907 |
| #13 | MeSH descriptor: [Stroke] explode all trees | 17550 |
| #14 | MeSH descriptor: [Brain Ischemia] explode all trees | 5904 |
| #15 | (Acute ischemic stroke):ti,ab,kw OR (Apoplexy):ti,ab,kw OR (Brain Vascular Accident):ti,ab,kw OR (Cerebral apoplexy):ti,ab,kw OR (Cerebral arterial thrombosis):ti,ab,kw | 10997 |
| #16 | (Cerebral disease):ti,ab,kw OR (Cerebral infarction):ti,ab,kw OR (Cerebral ischemia):ti,ab,kw OR (Cerebral Stroke):ti,ab,kw OR (Cerebrovascular Accident):ti,ab,kw | 32959 |
| #17 | (Cerebrovascular Apoplexy):ti,ab,kw OR (Cerebrovascular disease):ti,ab,kw OR (Cerebrovascular Stroke):ti,ab,kw OR (CVA):ti,ab,kw OR (Encephalorrhagia):ti,ab,kw | 24209 |
| #18 | (Hemorrhagic stroke):ti,ab,kw OR (Infarction of brain):ti,ab,kw OR (Ischemic stroke):ti,ab,kw OR (Stroke):ti,ab,kw | 75662 |
| #19 | #13 OR #14 OR #15 OR #16 OR #17 OR #18 | 88921 |
| #20 | MeSH descriptor: [Clinical Trials as Topic] explode all trees | 96117 |
| #21 | MeSH descriptor: [Random Allocation] explode all trees | 26024 |
| #22 | MeSH descriptor: [Double-Blind Method] explode all trees | 171464 |
| #23 | MeSH descriptor: [Single-Blind Method] explode all trees | 27497 |
| #24 | MeSH descriptor: [Control Groups] explode all trees | 335 |
| #25 | MeSH descriptor: [Cross-Over Studies] explode all trees | 48266 |
| #26 | (random*):ti,ab,kw OR (trial):ti,ab,kw OR (crossover):ti,ab,kw OR (cross-over):ti,ab,kw OR (open-label):ti,ab,kw | 1520750 |
| #27 | (non-masked):ti,ab,kw OR (unblinded):ti,ab,kw OR (Clinical Trial):pt | 5311 |
| #28 | #20 OR #21 OR #22 OR #23 OR #24 OR #25 OR #26 OR #27 | 1552364 |
| #29 | #12 AND #19 AND #28 | 326 |
| **China National Knowledge Infrastructure** | | |
| #1 | SU%=(传统运动 + 传统功法 + 气功 + 太极拳 + 八段锦 + 五禽戏 + 六字诀 + 太极 + 功法 + 养生功法 + 功夫 + 身心运动 + 导引术 + 易筋经 + 导引养生功十二法 + 十二段锦 + 马王堆导引术 + 大舞 + 明目功 + 武术) | 80000 |
| #2 | SU%=(卒中 + 脑卒中 + 脑出血 + 脑血栓 + 中风 + 脑缺血 + 缺血性卒中 + 出血性卒中 + 脑栓塞 + 脑梗塞) | 35.21 |
| #3 | #1 AND #2 | 470 |
| **Weipu database** | | |
| #1 | M=(传统运动 OR 传统功法 OR 气功 OR 太极拳 OR 八段锦 OR 五禽戏 OR 六字诀 OR 太极 OR 功法 OR 养生功法 OR 功夫 OR 身心运动 OR 导引术 OR 易筋经 OR 导引养生功十二法 OR 十二段锦 OR 马王堆导引术 OR 大舞 OR 明目功 OR 武术) | 121394 |
| #2 | M=(卒中 OR 脑卒中 OR 脑出血 OR 脑血栓 OR 中风 OR 脑缺血 OR 缺血性卒中 OR 出血性卒中 OR 脑栓塞 OR 脑梗塞) | 311,188 |
| #3 | #1 AND #2 | 341 |
| **Wanfang database** | | |
| #1 | 题名或关键词:("传统运动" OR "传统功法" OR "气功" OR "太极拳" OR "八段锦" OR "五禽戏" OR "六字诀" OR "太极" OR "功法" OR "养生功法" OR "功夫" OR "身心运动" OR "导引术" OR "易筋经" OR "导引养生功十二法" OR "十二段锦" OR "马王堆导引术" OR "大舞" OR "明目功" OR "武术") | 132,875 |
| #2 | 题名或关键词:("卒中" OR "脑卒中" OR "脑出血" OR "脑血栓" OR "中风" OR "脑缺血" OR "缺血性卒中" OR "出血性卒中" OR "脑栓塞"OR "脑梗塞") | 394,095 |
| #3 | #1 AND #2 | 387 |
| **Chinese biomedical literature database** | | |
| #1 | ("传统运动"[常用字段:智能] OR "传统功法"[常用字段:智能] OR "气功"[常用字段:智能] OR "太极拳"[常用字段:智能] OR "八段锦"[常用字段:智能] OR "五禽戏"[常用字段:智能] OR "六字诀"[常用字段:智能] OR "太极"[常用字段:智能] OR "功法"[常用字段:智能]) AND ( 临床试验[文献类型] OR 随机对照试验[文献类型] OR Meta分析[文献类型] OR 多中心研究[文献类型]) AND ( 人类[特征词]) | 5248 |
| #2 | ("养生功法"[常用字段:智能] OR "功夫"[常用字段:智能] OR "身心运动"[常用字段:智能] OR "导引术"[常用字段:智能] OR "易筋经"[常用字段:智能] OR "导引养生功十二法"[常用字段:智能] OR "十二段锦"[常用字段:智能] OR "马王堆导引术"[常用字段:智能] OR "大舞"[常用字段:智能]) AND ( 临床试验[文献类型] OR 随机对照试验[文献类型] OR 多中心研究[文献类型] OR Meta分析[文献类型]) AND ( 人类[特征词]) | 228 |
| #3 | ("明目功"[常用字段:智能] OR "武术"[常用字段:智能] OR "易筋经"[常用字段:智能] OR "导引养生功十二法"[常用字段:智能] OR "十二段锦"[常用字段:智能] OR "马王堆导引术"[常用字段:智能] OR "大舞"[常用字段:智能]) AND ( 临床试验[文献类型] OR 随机对照试验[文献类型] OR Meta分析[文献类型] OR 多中心研究[文献类型]) AND ( 人类[特征词]) | 731 |
| #4 | #1 AND #2 AND #3 | 5499 |
| #5 | ("卒中"[常用字段:智能] OR "脑卒中"[常用字段:智能] OR "脑出血"[常用字段:智能] OR "脑血栓"[常用字段:智能] OR "中风"[常用字段:智能] OR "脑缺血"[常用字段:智能] OR "缺血性卒中"[常用字段:智能] OR "出血性卒中"[常用字段:智能] OR "脑栓塞"[常用字段:智能] OR "脑梗塞"[常用字段:智能]) AND ( 临床试验[文献类型] OR 随机对照试验[文献类型] OR Meta分析[文献类型] OR 多中心研究[文献类型]) AND ( 人类[特征词]) | 118643 |

1. Certainty of evidence

| Table S2 CINeMA assessment for Upper limb motor ability | | | | | | | |
| --- | --- | --- | --- | --- | --- | --- | --- |
| Comparison | Within-study bias | Reporting bias | Indirectness | Imprecision | Heterogeneity | Incoherence | Confidence rating |
| Baduanjin:Usual care | Some concerns | Low risk | No concerns | No concerns | No concerns | No concerns | Moderate |
| Baduanjin:Usual rehabilitation | Some concerns | Low risk | No concerns | No concerns | No concerns | No concerns | Moderate |
| Daoyin:Usual care | No concerns | Low risk | No concerns | Some concerns | Some concerns | No concerns | Moderate |
| Taiji:Usual rehabilitation | Some concerns | Low risk | No concerns | No concerns | Some concerns | No concerns | Low |
| Baduanjin:Daoyin | No concerns | Low risk | No concerns | No concerns | Some concerns | No concerns | Moderate |
| Baduanjin:Taiji | Some concerns | Low risk | No concerns | No concerns | Some concerns | No concerns | Low |
| Daoyin:Taiji | Some concerns | Low risk | No concerns | Some concerns | Some concerns | No concerns | Low |
| Daoyin:Usual rehabilitation | Some concerns | Low risk | No concerns | Some concerns | Some concerns | No concerns | Low |
| Taiji:Usual care | Some concerns | Low risk | No concerns | No concerns | Some concerns | No concerns | Moderate |
| Usual care:Usual rehabilitation | Some concerns | Low risk | No concerns | Some concerns | Some concerns | No concerns | Low |
| The minimal clinically important difference for the upper limb Fugl-Meyer Assessment (FMA) is 4.25(1) | | | | | | | |

| **Table S3 CINeMA assessment for Lower limb motor ability** | | | | | | | |
| --- | --- | --- | --- | --- | --- | --- | --- |
| Comparison | Within-study bias | Reporting bias | Indirectness | Imprecision | Heterogeneity | Incoherence | Confidence rating |
| Baduanjin:Taiji | Some concerns | Low risk | No concerns | Some concerns | Some concerns | No concerns | Low |
| Baduanjin:Usual care | Some concerns | Low risk | No concerns | Some concerns | Some concerns | No concerns | Low |
| Baduanjin:Usual rehabilitation | Some concerns | Low risk | No concerns | Some concerns | Some concerns | No concerns | Low |
| Daoyin:Usual care | No concerns | Low risk | No concerns | Some concerns | Some concerns | No concerns | Moderate |
| Taiji:Usual rehabilitation | No concerns | Low risk | No concerns | No concerns | Major concerns | Major concerns | Low |
| Baduanjin:Daoyin | No concerns | Low risk | No concerns | Major concerns | No concerns | No concerns | Low |
| Daoyin:Taiji | Some concerns | Low risk | No concerns | Major concerns | No concerns | No concerns | Very low |
| Daoyin:Usual rehabilitation | Some concerns | Low risk | No concerns | Major concerns | No concerns | No concerns | Very low |
| Taiji:Usual care | Some concerns | Low risk | No concerns | No concerns | Major concerns | No concerns | Very low |
| Usual care:Usual rehabilitation | Some concerns | Low risk | No concerns | Major concerns | No concerns | No concerns | Very low |
| The minimal clinically important difference (MCID) for the lower limb Fugl-Meyer Assessment (FMA) is 3.5.(2) | | | | | | | |

| **Table S4 CINeMA assessment for Overall motor ability** | | | | | | | |
| --- | --- | --- | --- | --- | --- | --- | --- |
| Comparison | Within-study bias | Reporting bias | Indirectness | Imprecision | Heterogeneity | Incoherence | Confidence rating |
| Baduanjin:Usual care | Some concerns | Low risk | No concerns | Some concerns | Some concerns | No concerns | Low |
| Baduanjin:Usual rehabilitation | Some concerns | Low risk | No concerns | No concerns | Major concerns | No concerns | Very low |
| Daoyin:Usual care | No concerns | Low risk | No concerns | Major concerns | No concerns | No concerns | Low |
| Liuzijue:Usual rehabilitation | No concerns | Low risk | No concerns | Major concerns | No concerns | No concerns | Low |
| Taiji:Usual rehabilitation | Some concerns | Low risk | No concerns | Some concerns | Some concerns | No concerns | Low |
| Baduanjin:Daoyin | No concerns | Low risk | No concerns | Major concerns | No concerns | No concerns | Low |
| Baduanjin:Liuzijue | Some concerns | Low risk | No concerns | Some concerns | Some concerns | No concerns | Low |
| Baduanjin:Taiji | Some concerns | Low risk | No concerns | Major concerns | No concerns | No concerns | Very low |
| Daoyin:Liuzijue | Some concerns | Low risk | No concerns | Major concerns | No concerns | No concerns | Very low |
| Daoyin:Taiji | Some concerns | Low risk | No concerns | Major concerns | No concerns | No concerns | Very low |
| Daoyin:Usual rehabilitation | Some concerns | Low risk | No concerns | Major concerns | No concerns | No concerns | Very low |
| Liuzijue:Taiji | No concerns | Low risk | No concerns | Major concerns | No concerns | No concerns | Low |
| Liuzijue:Usual care | Some concerns | Low risk | No concerns | Major concerns | No concerns | No concerns | Very low |
| Taiji:Usual care | Some concerns | Low risk | No concerns | Major concerns | No concerns | No concerns | Very low |
| Usual care:Usual rehabilitation | Some concerns | Low risk | No concerns | Major concerns | No concerns | No concerns | Very low |
| The minimal clinically important difference (MCID) for the overall Fugl-Meyer Assessment (FMA) is 4.(3) | | | | | | | |

| **Table S5 CINeMA assessment for Walking ability** | | | | | | | |
| --- | --- | --- | --- | --- | --- | --- | --- |
| Comparison | Within-study bias | Reporting bias | Indirectness | Imprecision | Heterogeneity | Incoherence | Confidence rating |
| Baduanjin:Usual rehabilitation | Some concerns | Low risk | No concerns | No concerns | Some concerns | No concerns | Low |
| Taiji:Usual care | No concerns | Low risk | No concerns | Some concerns | Some concerns | No concerns | Moderate |
| Taiji:Usual rehabilitation | Some concerns | Low risk | No concerns | No concerns | Major concerns | No concerns | Very low |
| Baduanjin:Taiji | Some concerns | Low risk | No concerns | Some concerns | Some concerns | No concerns | Low |
| Baduanjin:Usual care | Some concerns | Low risk | No concerns | No concerns | Major concerns | No concerns | Very low |
| Usual care:Usual rehabilitation | Some concerns | Low risk | No concerns | Major concerns | No concerns | No concerns | Very low |
| According to Cohen's d, an effect size with a standardized mean difference (SMD) greater than 0.2 is considered the minimal clinically important difference (MCID).(4) | | | | | | | |

| **Table S6 CINeMA assessment for Balance ability** | | | | | | | |
| --- | --- | --- | --- | --- | --- | --- | --- |
| Comparison | Within-study bias | Reporting bias | Indirectness | Imprecision | Heterogeneity | Incoherence | Confidence rating |
| Baduanjin:Usual care | Some concerns | Low risk | No concerns | No concerns | Major concerns | No concerns | Very low |
| Baduanjin:Usual rehabilitation | Some concerns | Low risk | No concerns | No concerns | Major concerns | No concerns | Very low |
| Daoyin:Usual care | No concerns | Low risk | No concerns | Major concerns | No concerns | No concerns | Low |
| Liuzijue:Usual rehabilitation | No concerns | Low risk | No concerns | No concerns | Major concerns | No concerns | Low |
| Taiji:Usual care | No concerns | Low risk | No concerns | No concerns | Major concerns | No concerns | Low |
| Taiji:Usual rehabilitation | Some concerns | Low risk | No concerns | No concerns | Major concerns | No concerns | Very low |
| Usual rehabilitation:Wuqinxi | Some concerns | Low risk | No concerns | No concerns | Major concerns | No concerns | Very low |
| Baduanjin:Daoyin | No concerns | Low risk | No concerns | Major concerns | No concerns | No concerns | Low |
| Baduanjin:Liuzijue | No concerns | Low risk | No concerns | Major concerns | No concerns | No concerns | Low |
| Baduanjin:Taiji | Some concerns | Low risk | No concerns | Major concerns | No concerns | No concerns | Very low |
| Baduanjin:Wuqinxi | Some concerns | Low risk | No concerns | Major concerns | No concerns | No concerns | Very low |
| Daoyin:Liuzijue | No concerns | Low risk | No concerns | Major concerns | No concerns | No concerns | Low |
| Daoyin:Taiji | No concerns | Low risk | No concerns | Major concerns | No concerns | No concerns | Low |
| Daoyin:Usual rehabilitation | No concerns | Low risk | No concerns | Major concerns | No concerns | No concerns | Low |
| Daoyin:Wuqinxi | Some concerns | Low risk | No concerns | Major concerns | No concerns | No concerns | Very low |
| Liuzijue:Taiji | No concerns | Low risk | No concerns | Major concerns | No concerns | No concerns | Low |
| Liuzijue:Usual care | No concerns | Low risk | No concerns | Some concerns | Some concerns | No concerns | Moderate |
| Liuzijue:Wuqinxi | Some concerns | Low risk | No concerns | Major concerns | No concerns | No concerns | Very low |
| Taiji:Wuqinxi | Some concerns | Low risk | No concerns | Major concerns | No concerns | No concerns | Very low |
| Usual care:Usual rehabilitation | Some concerns | Low risk | No concerns | Major concerns | No concerns | No concerns | Very low |
| Usual care:Wuqinxi | Some concerns | Low risk | No concerns | Some concerns | Some concerns | No concerns | Low |
| According to Cohen's d, an effect size with a standardized mean difference (SMD) greater than 0.2 is considered the minimal clinically important difference (MCID).(4) | | | | | | | |

| **Table S7 CINeMA assessment for Self-care ability** | | | | | | | |
| --- | --- | --- | --- | --- | --- | --- | --- |
| Comparison | Within-study bias | Reporting bias | Indirectness | Imprecision | Heterogeneity | Incoherence | Confidence rating |
| Baduanjin:Usual rehabilitation | Some concerns | Low risk | No concerns | No concerns | Major concerns | No concerns | Very low |
| Daoyin:Usual care | No concerns | Low risk | No concerns | Major concerns | No concerns | No concerns | Low |
| Liuzijue:Usual rehabilitation | No concerns | Low risk | No concerns | Major concerns | No concerns | No concerns | Low |
| Taiji:Usual care | Some concerns | Low risk | No concerns | Major concerns | No concerns | No concerns | Very low |
| Taiji:Usual rehabilitation | Some concerns | Low risk | No concerns | Some concerns | Some concerns | No concerns | Low |
| Baduanjin:Daoyin | Some concerns | Low risk | No concerns | Major concerns | No concerns | No concerns | Very low |
| Baduanjin:Liuzijue | No concerns | Low risk | No concerns | Major concerns | No concerns | No concerns | Low |
| Baduanjin:Taiji | Some concerns | Low risk | No concerns | Major concerns | No concerns | No concerns | Very low |
| Baduanjin:Usual care | Some concerns | Low risk | No concerns | Major concerns | No concerns | No concerns | Very low |
| Daoyin:Liuzijue | Some concerns | Low risk | No concerns | Major concerns | No concerns | No concerns | Very low |
| Daoyin:Taiji | No concerns | Low risk | No concerns | Major concerns | No concerns | No concerns | Low |
| Daoyin:Usual rehabilitation | Some concerns | Low risk | No concerns | Major concerns | No concerns | No concerns | Very low |
| Liuzijue:Taiji | Some concerns | Low risk | No concerns | Major concerns | No concerns | No concerns | Very low |
| Liuzijue:Usual care | Some concerns | Low risk | No concerns | Major concerns | No concerns | No concerns | Very low |
| Usual care:Usual rehabilitation | Some concerns | Low risk | No concerns | Major concerns | No concerns | No concerns | Very low |
| The minimal clinically important difference (MCID) for the Barthel Index is 1.85.(5) | | | | | | | |

| **Table S8 CINeMA assessment for Cognitive functioning** | | | | | | | |
| --- | --- | --- | --- | --- | --- | --- | --- |
| Comparison | Within-study bias | Reporting bias | Indirectness | Imprecision | Heterogeneity | Incoherence | Confidence rating |
| Baduanjin:Usual care | No concerns | Low risk | No concerns | No concerns | No concerns | No concerns | High |
| Baduanjin:Usual rehabilitation | No concerns | Low risk | No concerns | No concerns | No concerns | No concerns | High |
| Liuzijue:Usual care | No concerns | Low risk | No concerns | No concerns | No concerns | No concerns | High |
| Taiji:Usual care | No concerns | Low risk | No concerns | Major concerns | No concerns | No concerns | Low |
| Baduanjin:Liuzijue | No concerns | Low risk | No concerns | Some concerns | No concerns | No concerns | Moderate |
| Baduanjin:Taiji | No concerns | Low risk | No concerns | Major concerns | No concerns | No concerns | Low |
| Liuzijue:Taiji | No concerns | Low risk | No concerns | Major concerns | No concerns | No concerns | Low |
| Liuzijue:Usual rehabilitation | No concerns | Low risk | No concerns | No concerns | No concerns | No concerns | High |
| Taiji:Usual rehabilitation | No concerns | Low risk | No concerns | Some concerns | No concerns | No concerns | Moderate |
| Usual care:Usual rehabilitation | No concerns | Low risk | No concerns | Some concerns | No concerns | No concerns | Moderate |
| According to Cohen's d, an effect size with a standardized mean difference (SMD) greater than 0.2 is considered the minimal clinically important difference (MCID).(4) | | | | | | | |

| **Table S9 CINeMA assessment for Depression** | | | | | | | |
| --- | --- | --- | --- | --- | --- | --- | --- |
| Comparison | Within-study bias | Reporting bias | Indirectness | Imprecision | Heterogeneity | Incoherence | Confidence rating |
| Baduanjin:Usual rehabilitation | No concerns | Low risk | No concerns | Major concerns | No concerns | No concerns | Low |
| Daoyin:Usual care | No concerns | Low risk | No concerns | Major concerns | No concerns | No concerns | Low |
| Liuzijue:Usual care | Some concerns | Low risk | No concerns | No concerns | Major concerns | No concerns | Very low |
| Liuzijue:Usual rehabilitation | Some concerns | Low risk | No concerns | Some concerns | Some concerns | No concerns | Low |
| Taiji:Usual care | No concerns | Low risk | No concerns | No concerns | Major concerns | No concerns | Low |
| Taiji:Usual rehabilitation | Some concerns | Low risk | No concerns | Major concerns | No concerns | No concerns | Very low |
| Usual rehabilitation:Yijinjing | Some concerns | Low risk | No concerns | Major concerns | No concerns | No concerns | Very low |
| Baduanjin:Daoyin | No concerns | Low risk | No concerns | Major concerns | No concerns | No concerns | Low |
| Baduanjin:Liuzijue | No concerns | Low risk | No concerns | Major concerns | No concerns | No concerns | Low |
| Baduanjin:Taiji | No concerns | Low risk | No concerns | Major concerns | No concerns | No concerns | Low |
| Baduanjin:Usual care | No concerns | Low risk | No concerns | Major concerns | No concerns | No concerns | Low |
| Baduanjin:Yijinjing | No concerns | Low risk | No concerns | Major concerns | No concerns | No concerns | Low |
| Daoyin:Liuzijue | No concerns | Low risk | No concerns | Major concerns | No concerns | No concerns | Low |
| Daoyin:Taiji | No concerns | Low risk | No concerns | Major concerns | No concerns | No concerns | Low |
| Daoyin:Usual rehabilitation | No concerns | Low risk | No concerns | Major concerns | No concerns | No concerns | Low |
| Daoyin:Yijinjing | No concerns | Low risk | No concerns | Major concerns | No concerns | No concerns | Low |
| Liuzijue:Taiji | Some concerns | Low risk | No concerns | Major concerns | No concerns | No concerns | Very low |
| Liuzijue:Yijinjing | Some concerns | Low risk | No concerns | Major concerns | No concerns | No concerns | Very low |
| Taiji:Yijinjing | Some concerns | Low risk | No concerns | Major concerns | No concerns | No concerns | Very low |
| Usual care:Usual rehabilitation | Some concerns | Low risk | No concerns | Major concerns | No concerns | No concerns | Very low |
| Usual care:Yijinjing | Some concerns | Low risk | No concerns | Major concerns | No concerns | No concerns | Very low |
| According to Cohen's d, an effect size with a standardized mean difference (SMD) greater than 0.2 is considered the minimal clinically important difference (MCID).(4) | | | | | | | |

| **Table S10 CINeMA assessment for Quality of life** | | | | | | | |
| --- | --- | --- | --- | --- | --- | --- | --- |
| Comparison | Within-study bias | Reporting bias | Indirectness | Imprecision | Heterogeneity | Incoherence | Confidence rating |
| Baduanjin:Usual care | Some concerns | Low risk | No concerns | No concerns | Major concerns | No concerns | Very low |
| Baduanjin:Usual rehabilitation | Some concerns | Low risk | No concerns | No concerns | Major concerns | No concerns | Very low |
| Taiji:Usual care | No concerns | Low risk | No concerns | Major concerns | No concerns | No concerns | Low |
| Taiji:Usual rehabilitation | No concerns | Low risk | No concerns | Major concerns | No concerns | No concerns | Low |
| Usual rehabilitation:Wuqinxi | Some concerns | Low risk | No concerns | Major concerns | No concerns | No concerns | Low |
| Baduanjin:Taiji | Some concerns | Low risk | No concerns | Major concerns | No concerns | No concerns | Low |
| Baduanjin:Wuqinxi | Some concerns | Low risk | No concerns | Major concerns | No concerns | No concerns | Very low |
| Taiji:Wuqinxi | Some concerns | Low risk | No concerns | Major concerns | No concerns | No concerns | Very low |
| Usual care:Usual rehabilitation | Some concerns | Low risk | No concerns | Major concerns | No concerns | No concerns | Very low |
| Usual care:Wuqinxi | Some concerns | Low risk | No concerns | Major concerns | No concerns | No concerns | Very low |
| According to Cohen's d, an effect size with a standardized mean difference (SMD) greater than 0.2 is considered the minimal clinically important difference (MCID).(4) | | | | | | | |

| **Table S11 CINeMA assessment for Quality of sleep** | | | | | | | |
| --- | --- | --- | --- | --- | --- | --- | --- |
| Comparison | Within-study bias | Reporting bias | Indirectness | Imprecision | Heterogeneity | Incoherence | Confidence rating |
| Baduanjin:Usual care | No concerns | Low risk | No concerns | No concerns | No concerns | No concerns | High |
| Daoyin:Usual care | No concerns | Low risk | No concerns | Major concerns | No concerns | No concerns | Low |
| Taiji:Usual care | No concerns | Low risk | No concerns | Major concerns | No concerns | No concerns | Low |
| Taiji:Usual rehabilitation | No concerns | Low risk | No concerns | Major concerns | No concerns | No concerns | Low |
| Usual rehabilitation:Wuqinxi | Some concerns | Low risk | No concerns | No concerns | No concerns | No concerns | Moderate |
| Baduanjin:Daoyin | No concerns | Low risk | No concerns | Major concerns | No concerns | No concerns | Low |
| Baduanjin:Taiji | No concerns | Low risk | No concerns | Some concerns | No concerns | No concerns | Moderate |
| Baduanjin:Usual rehabilitation | No concerns | Low risk | No concerns | No concerns | No concerns | No concerns | High |
| Baduanjin:Wuqinxi | No concerns | Low risk | No concerns | Major concerns | No concerns | No concerns | Low |
| Daoyin:Taiji | No concerns | Low risk | No concerns | Major concerns | No concerns | No concerns | Low |
| Daoyin:Usual rehabilitation | No concerns | Low risk | No concerns | Major concerns | No concerns | No concerns | Low |
| Daoyin:Wuqinxi | No concerns | Low risk | No concerns | Major concerns | No concerns | No concerns | Low |
| Taiji:Wuqinxi | No concerns | Low risk | No concerns | Major concerns | No concerns | No concerns | Low |
| Usual care:Usual rehabilitation | No concerns | Low risk | No concerns | Major concerns | No concerns | No concerns | Low |
| Usual care:Wuqinxi | No concerns | Low risk | No concerns | No concerns | No concerns | No concerns | High |
| According to Cohen's d, an effect size with a standardized mean difference (SMD) greater than 0.2 is considered the minimal clinically important difference (MCID).(4) | | | | | | | |

1. Network evidence Plot

The network evidence plot represents each intervention as a node, with the size of the node proportional to the corresponding sample size. The lines between nodes indicate direct comparisons between the interventions, and the width of the lines is proportional to the number of direct comparisons. This plot allows us to understand the relationships between the studies included in the analysis.


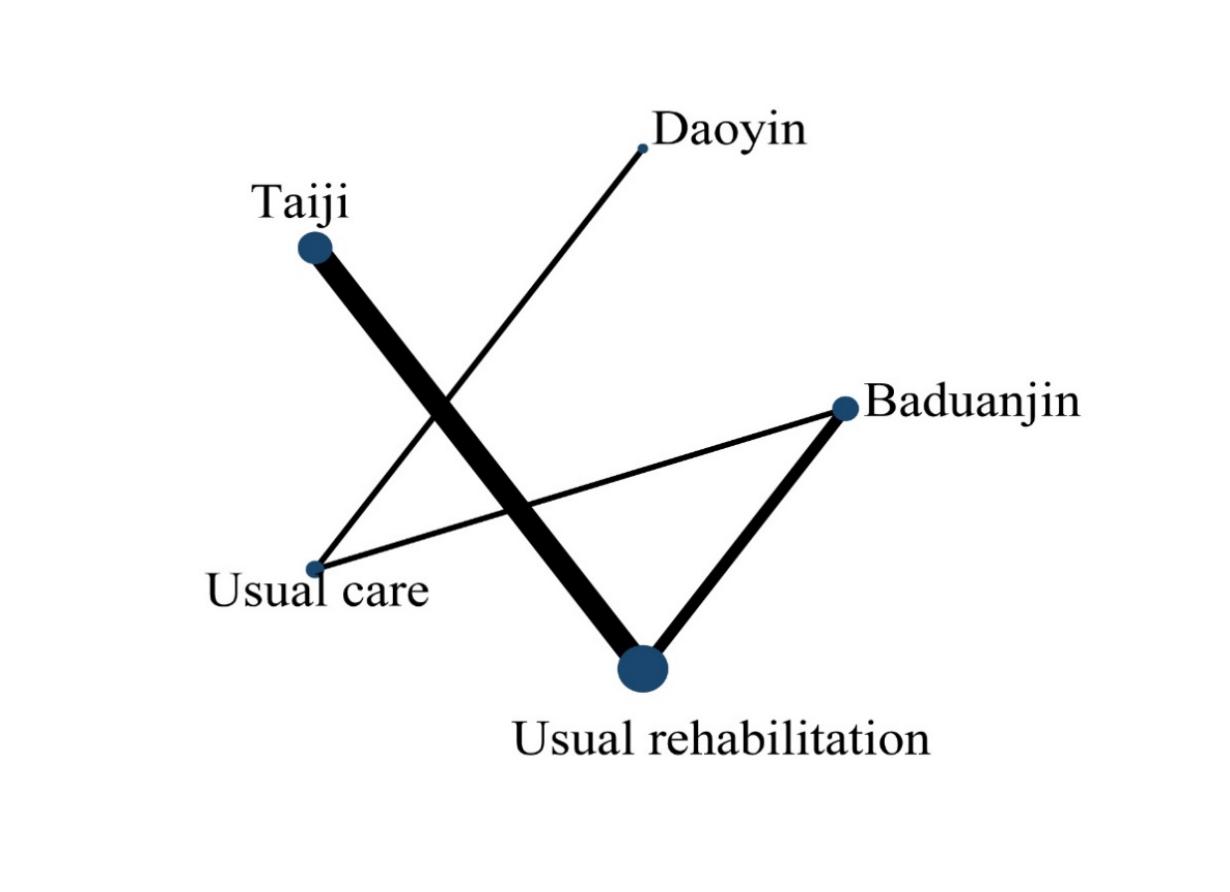


Figure S1 Network evidence Plot for Upper limb motor ability


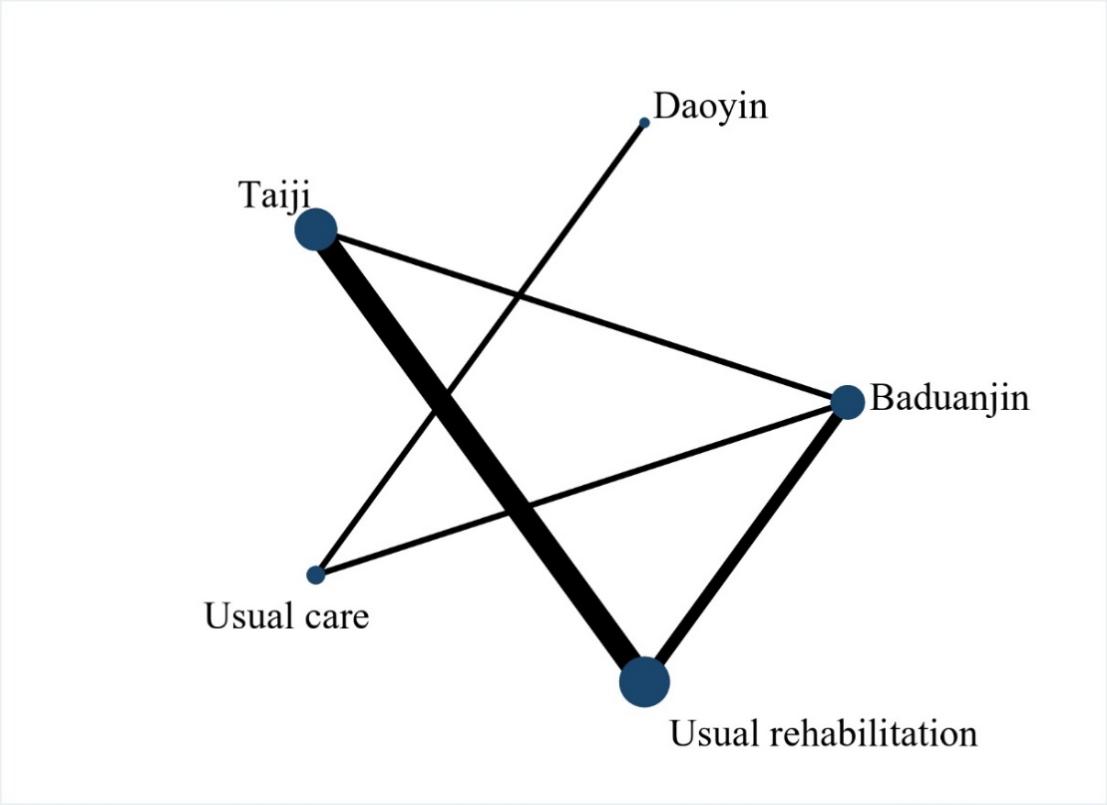


Figure S2 Network evidence Plot for Lower limb motor ability


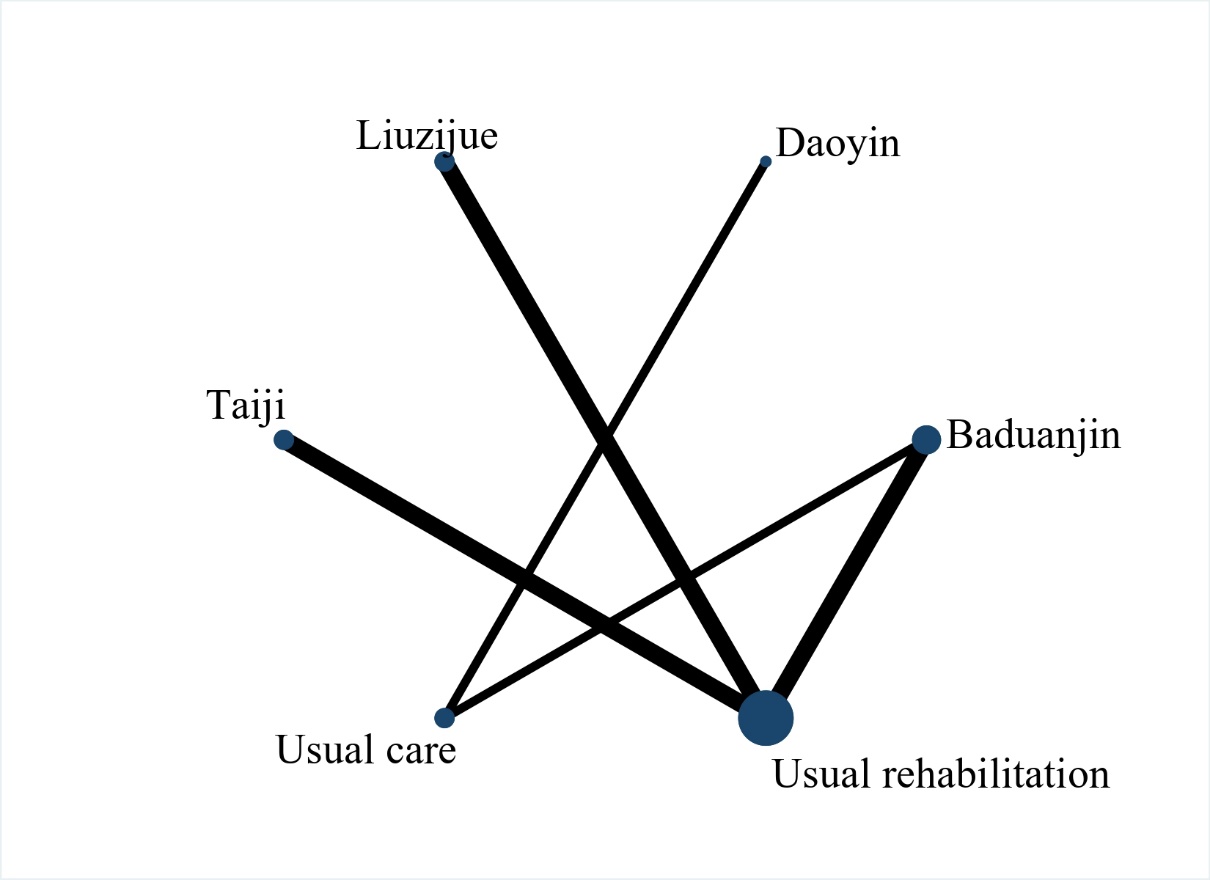


**Figure S3 Network evidence Plot for Overall motor ability**

**
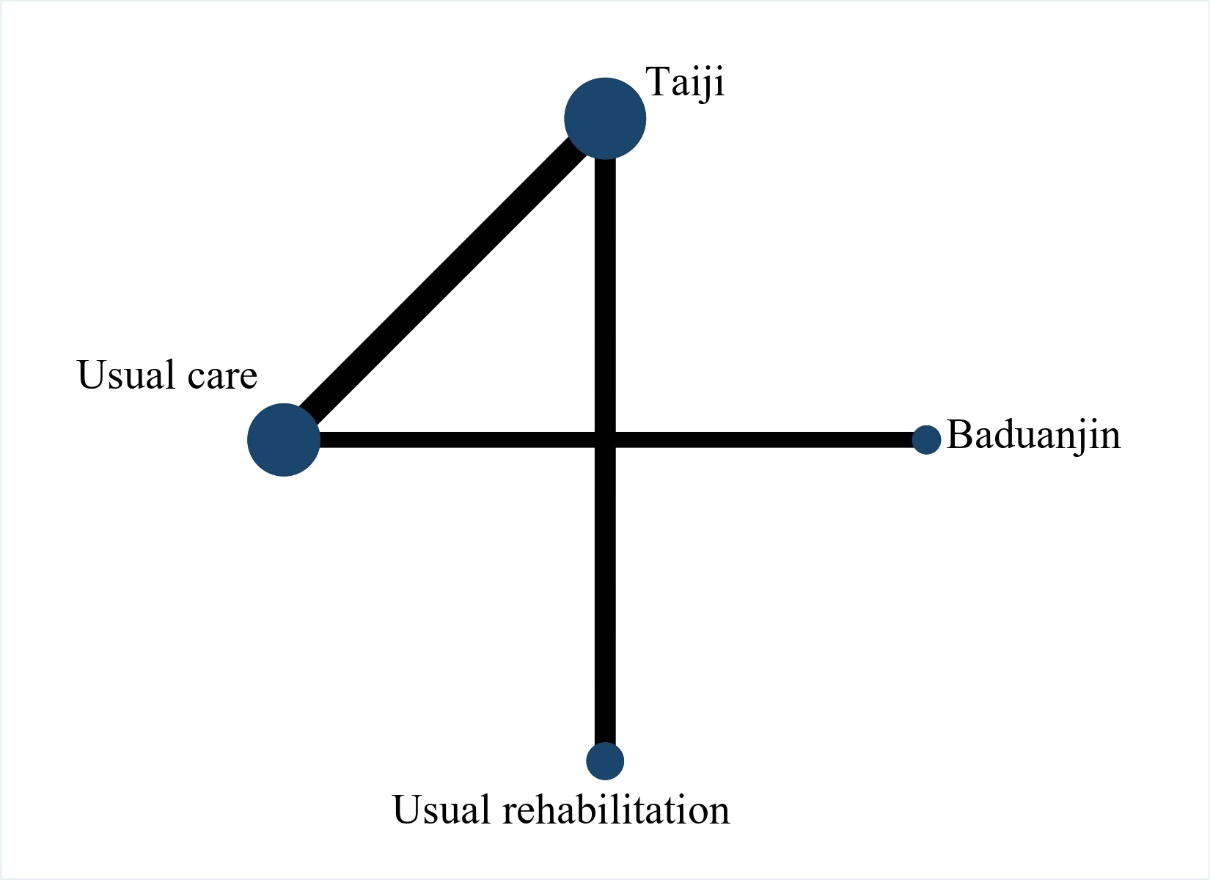
**

**Figure S4 Network evidence Plot for Walking ability**

**
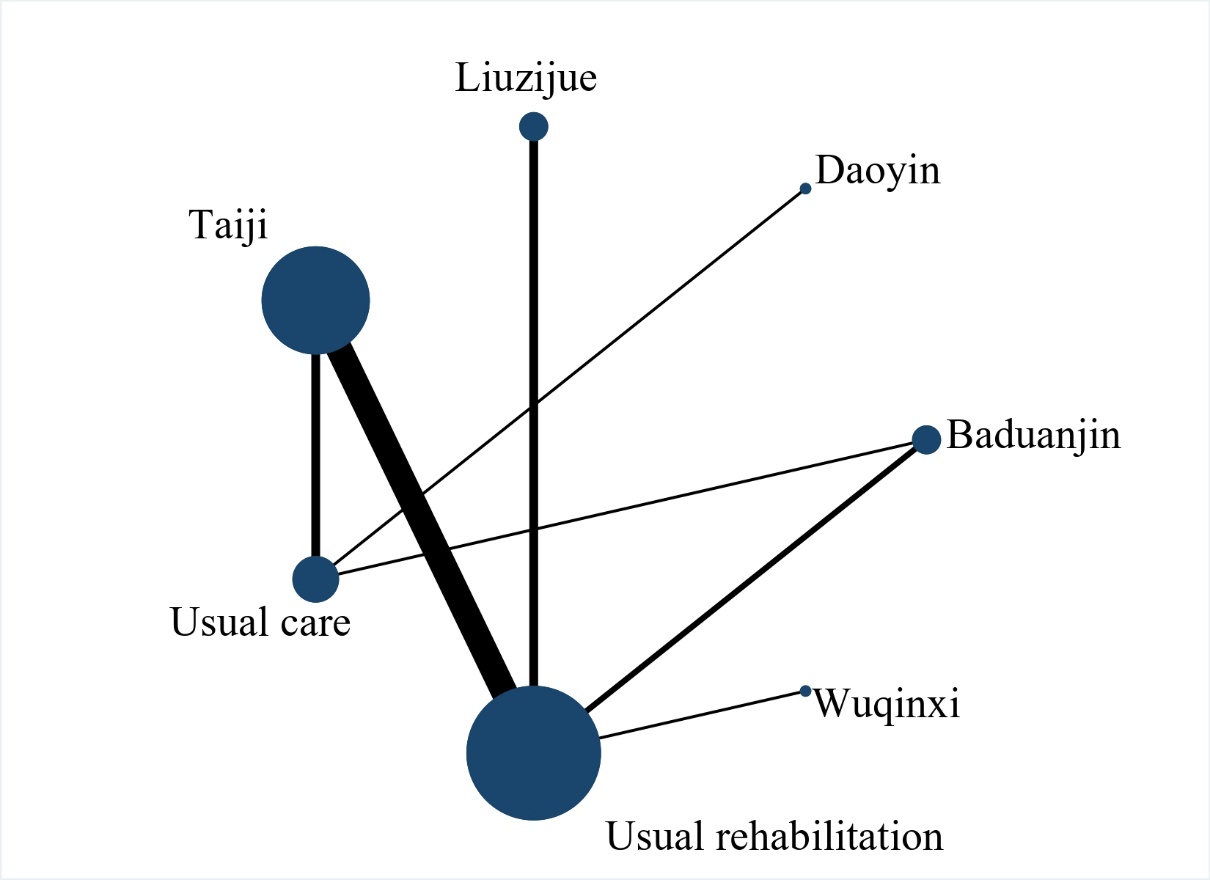
**

**Figure S5 Network evidence Plot for Balance ability**

**
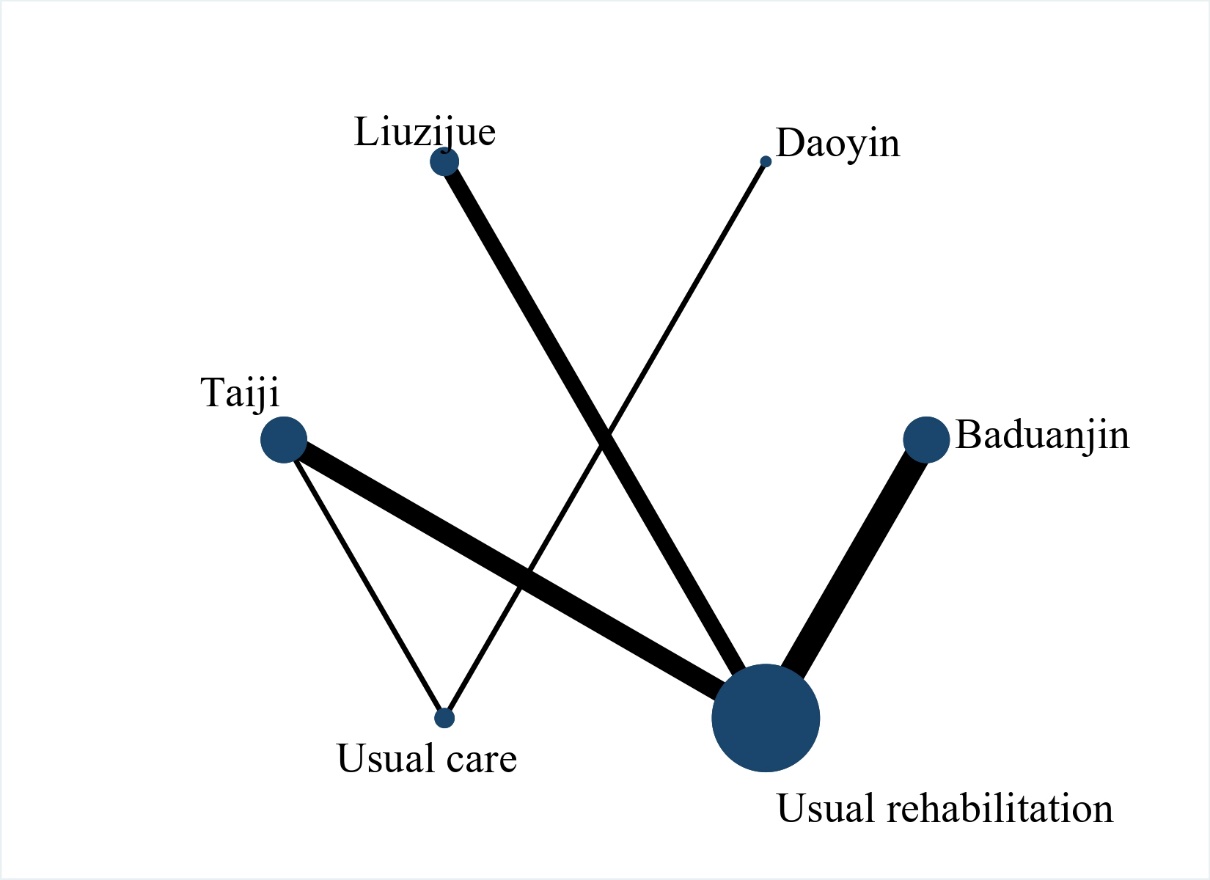
**

**Figure S6 Network evidence Plot for Self-care ability**

**
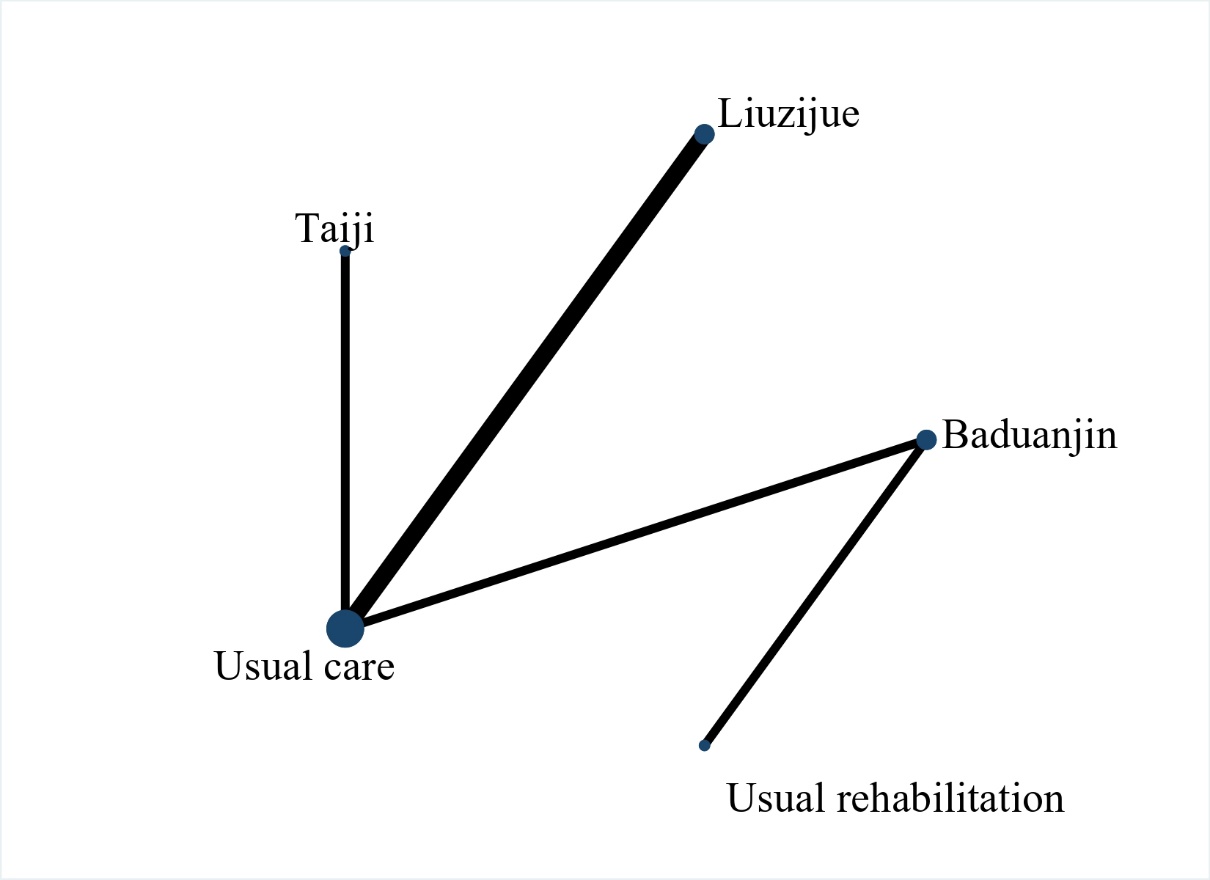
**

**Figure S7 Network evidence Plot for Cognitive functioning**

**
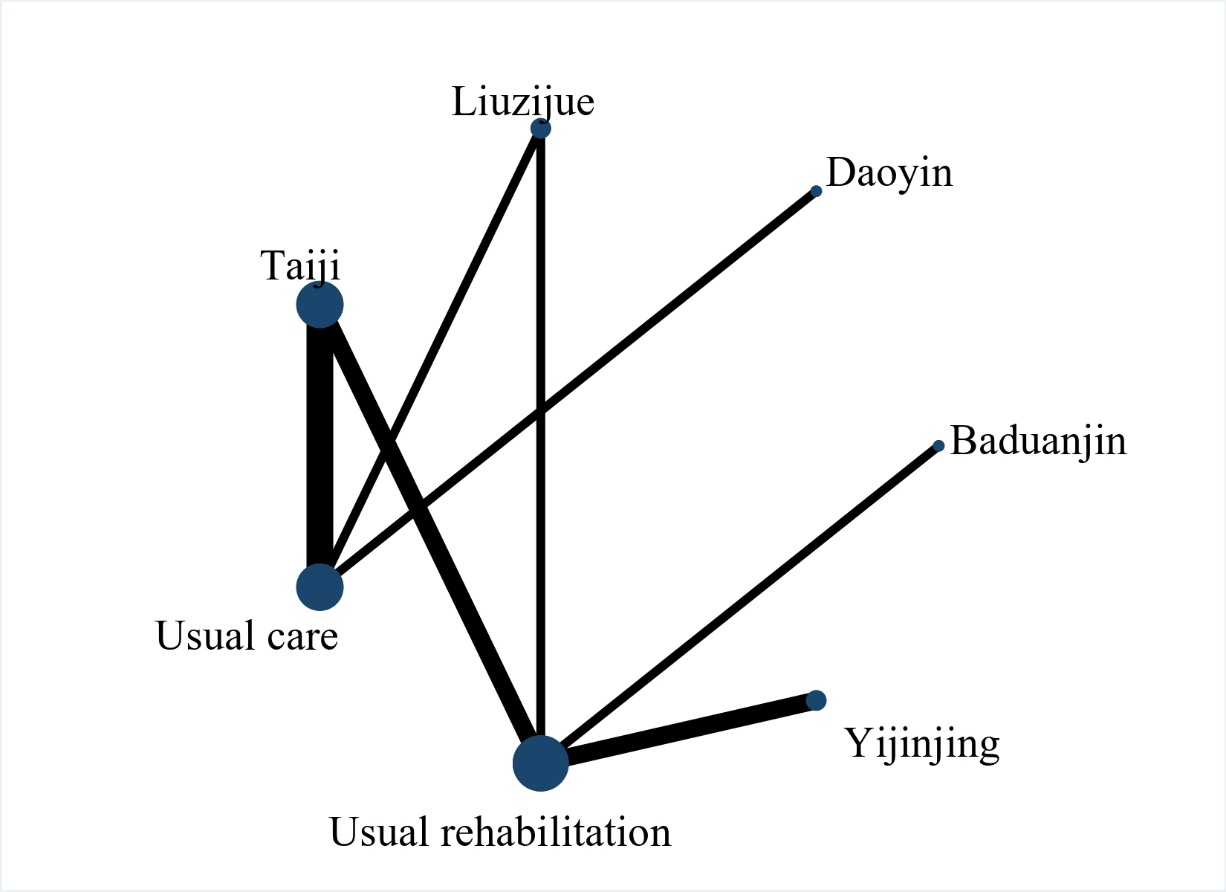
**

**Figure S8 Network evidence Plot for Depression**

**
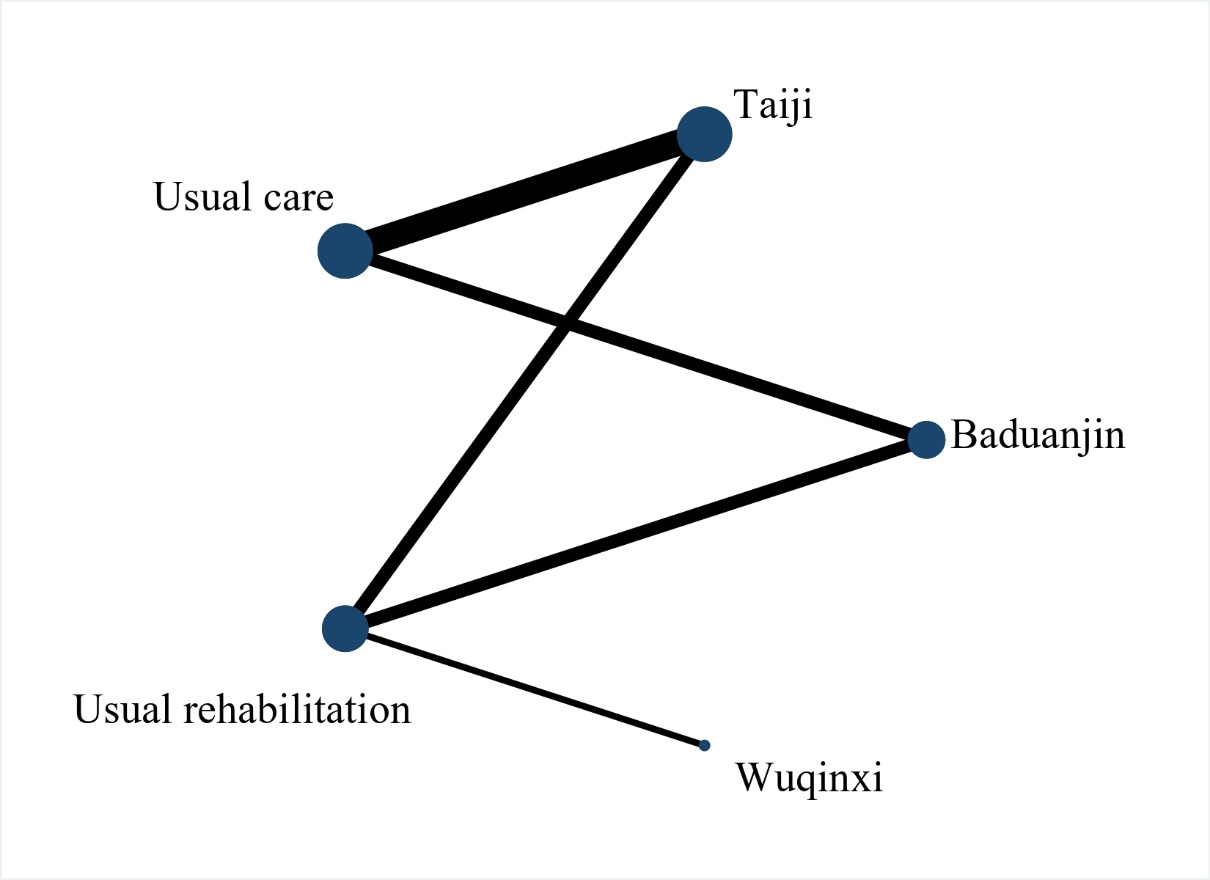
**

**Figure S9 Network evidence Plot for Quality of life**

**
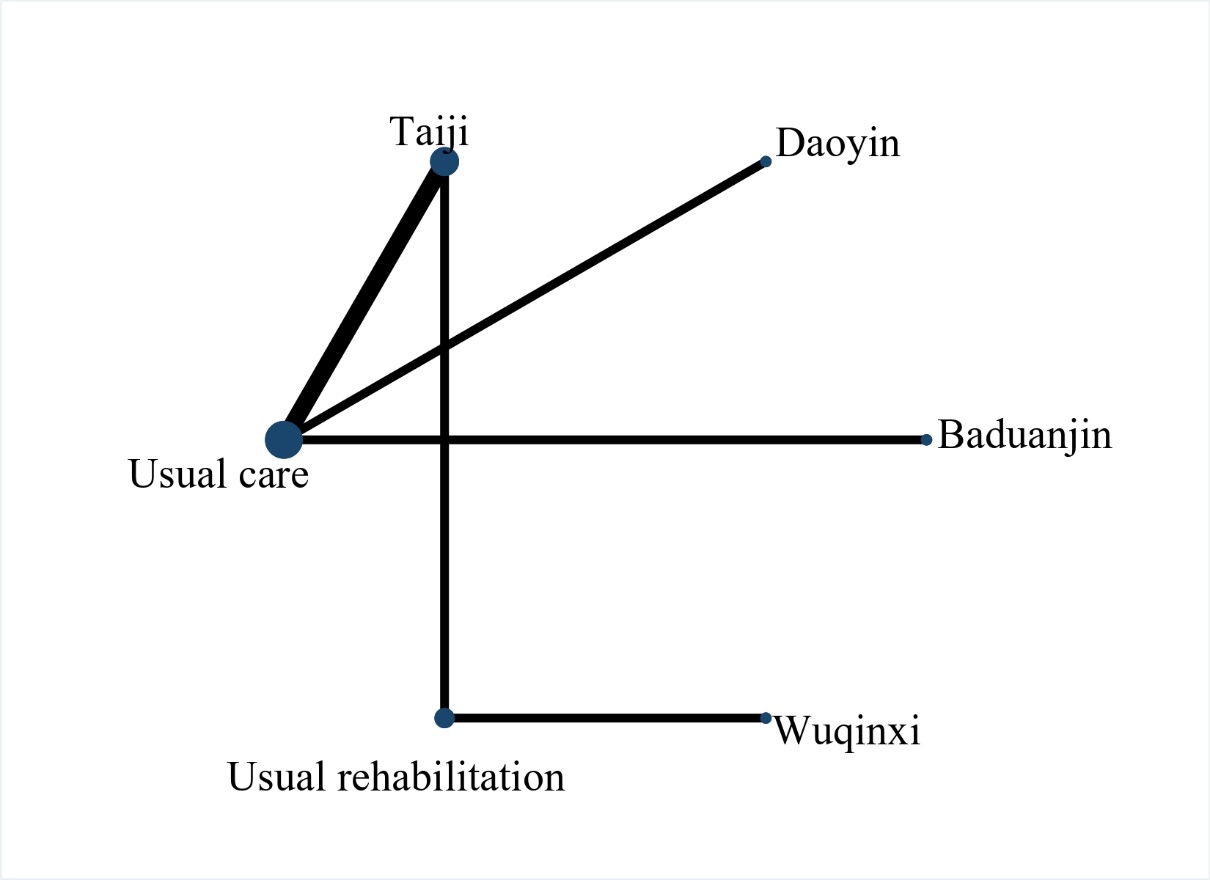
**

**Figure S10 Network evidence Plot for Quality of sleep**

1. Corrected Comparison Funnel Plot

We plotted corrected comparison funnels only for indicators with more than 10 included studies. Corrected comparison funnel plots were used to detect publication bias; if the scatter was roughly symmetrical, there was no significant publication bias


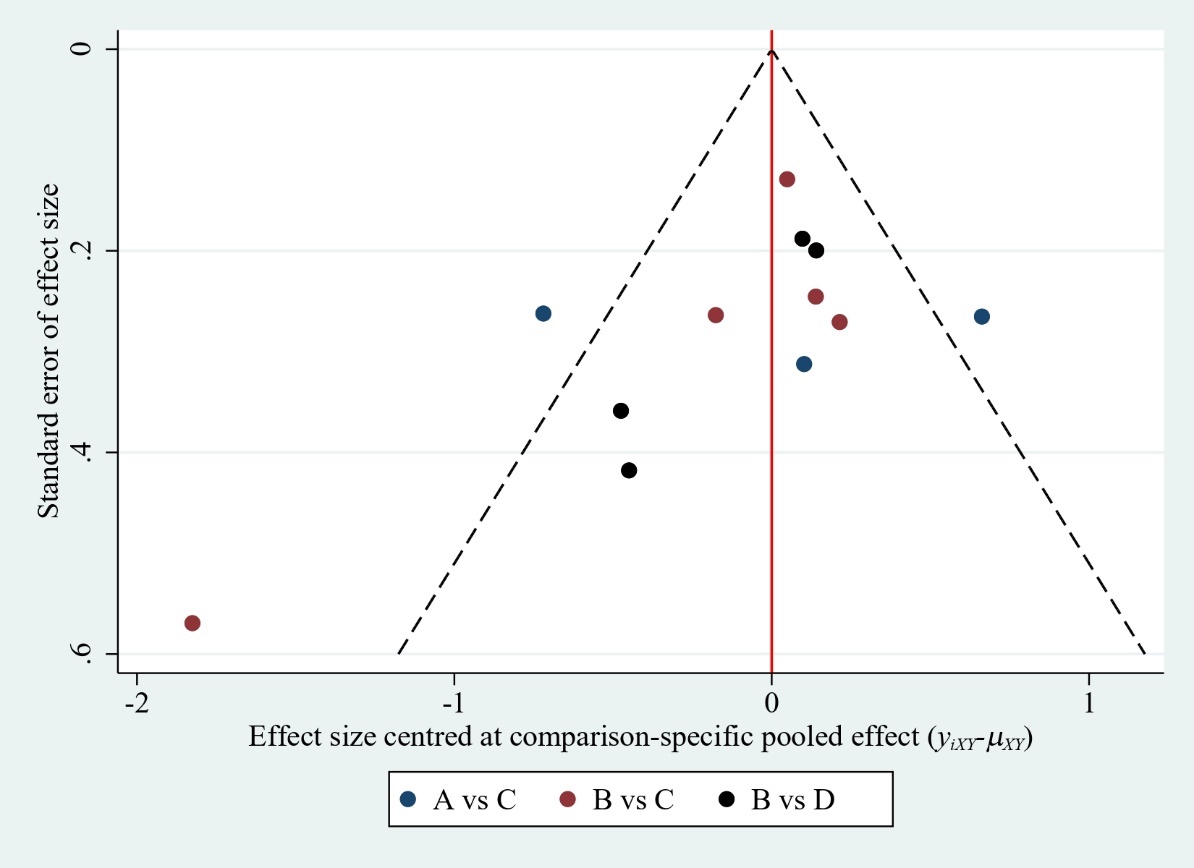


Figure S11 Corrected Comparison Funnel Plot for Walking Ability


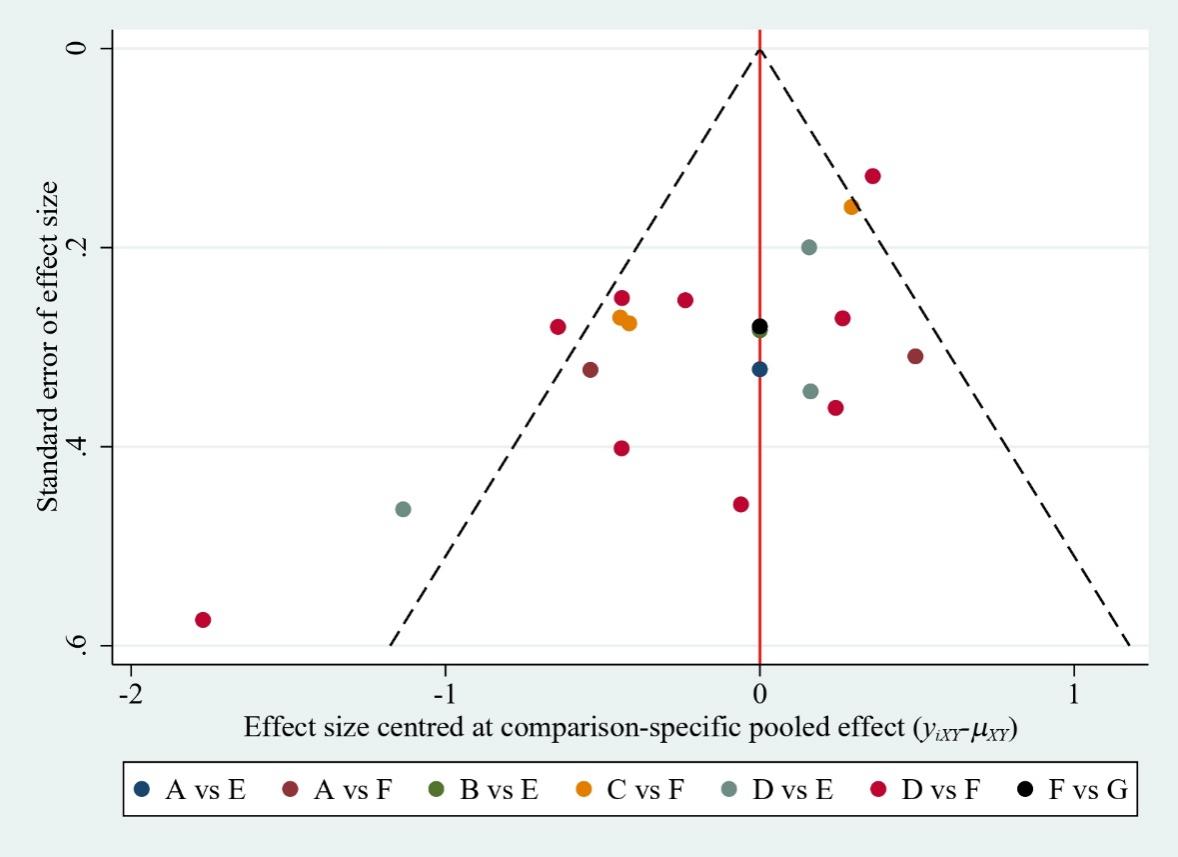


Figure S12 Corrected Comparison Funnel Plot for Balance Ability


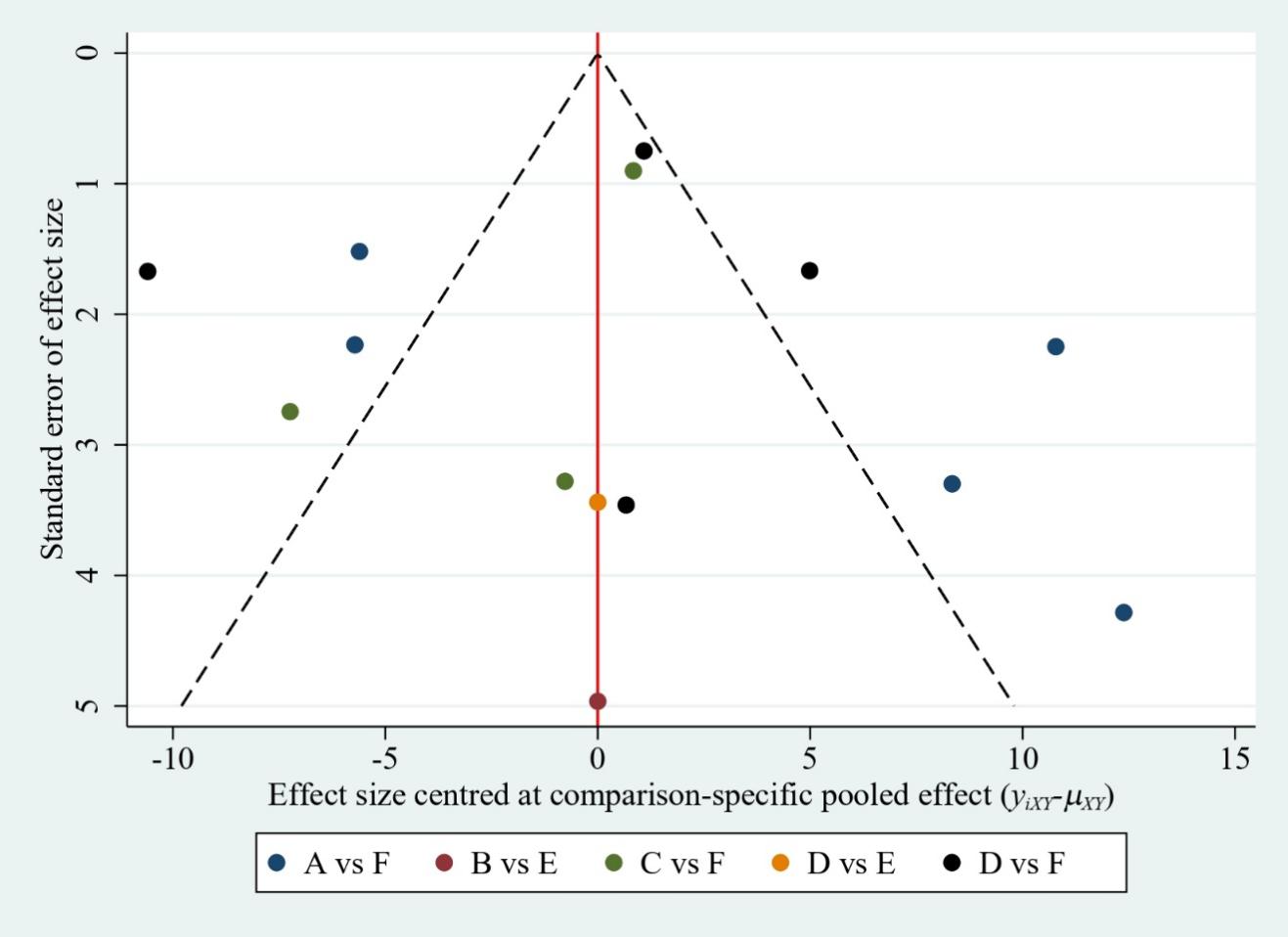


Figure S13 Corrected Comparison Funnel Plot for Self-care ability


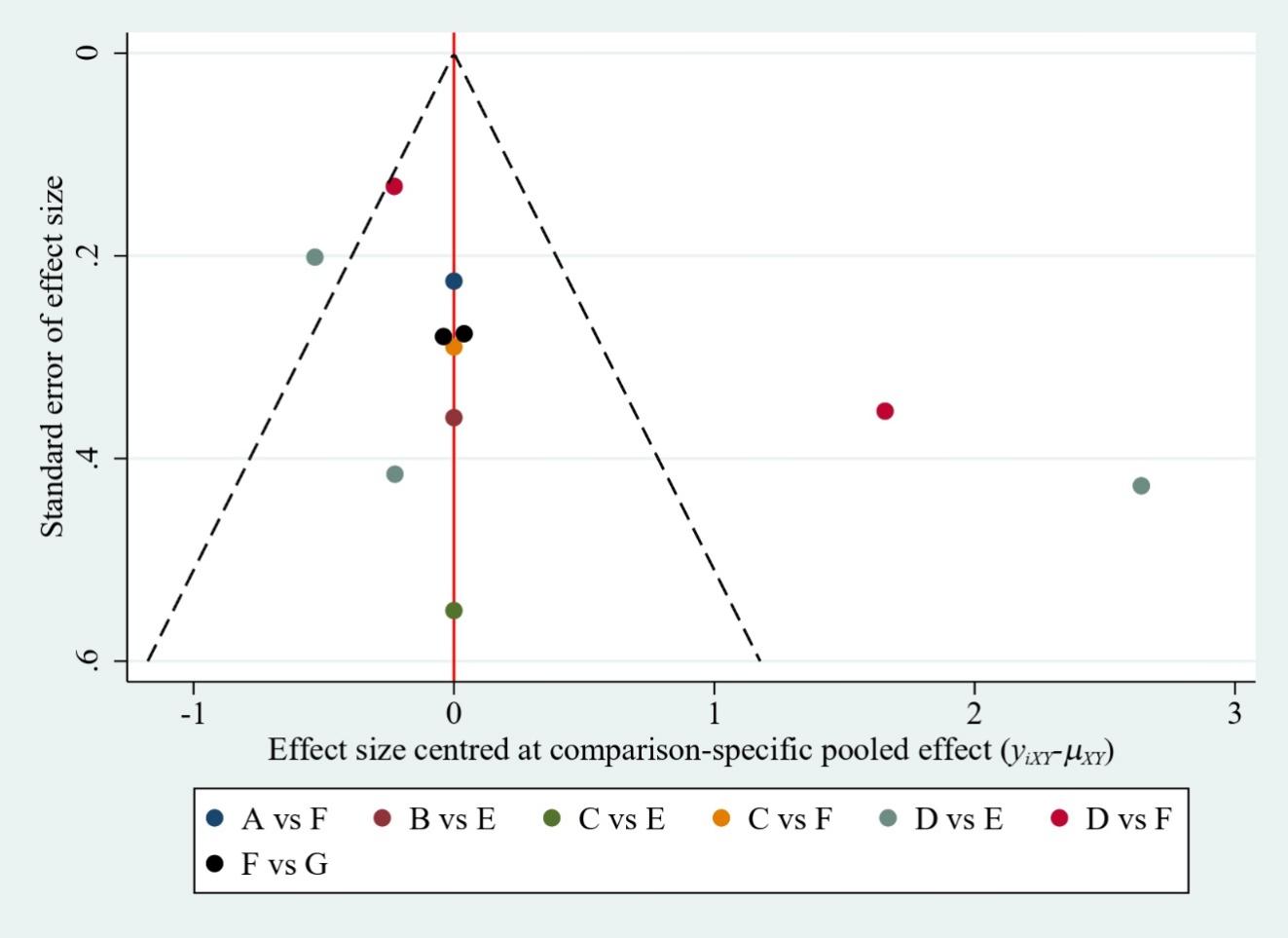


Figure S14 Corrected Comparison Funnel Plot for Depression


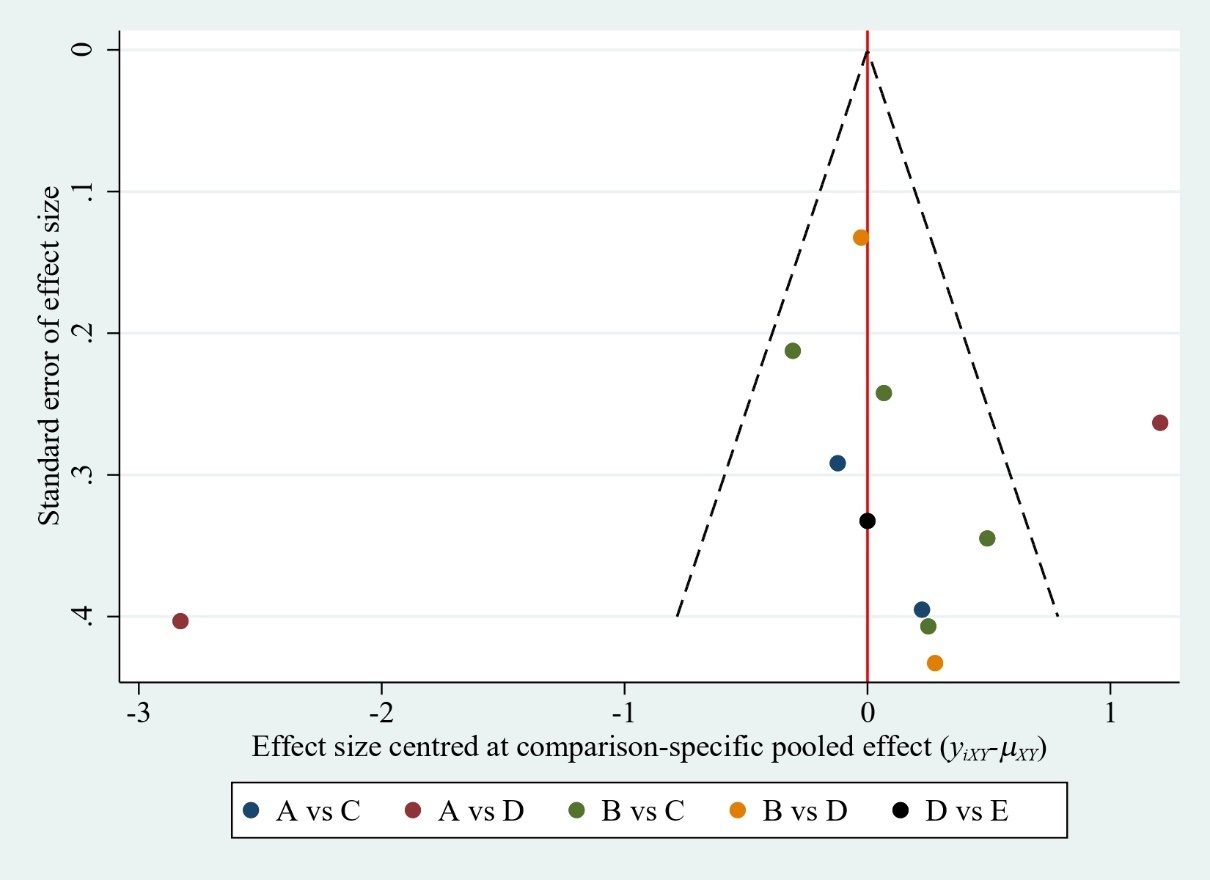


Figure S15 Corrected Comparison Funnel Plot for Quality of life

1. Sensitivity analysis

| **Table S12 Results of Sensitivity analysis** | | | | | | | | | | | | | | | | | | | | | | | | | | | | | | | | | | | | | | | | | |
| --- | --- | --- | --- | --- | --- | --- | --- | --- | --- | --- | --- | --- | --- | --- | --- | --- | --- | --- | --- | --- | --- | --- | --- | --- | --- | --- | --- | --- | --- | --- | --- | --- | --- | --- | --- | --- | --- | --- | --- | --- | --- |
| **Upper limb motor ability [MD (95%CI)]** | | | | | | | | | | | | | | | | | | | | | | | | | | | | | | | | | | | | | | | | | |
| Baduanjin | | | | | | | |  | | | | | | | | |  | | | | | | | | | |  | | | | | | | |  | | | | | | |
| 2.79 (-5.11,10.69) | | | | | | | | Taiji | | | | | | | | |  | | | | | | | | | |  | | | | | | | |  | | | | | | |
| 1.92 (-0.75,4.59) | | | | | | | | -0.87 (-9.21,7.47) | | | | | | | | | Daoyin | | | | | | | | | |  | | | | | | | |  | | | | | | |
| **4.56 (2.78,6.35)*** | | | | | | | | 1.77 (-6.33,9.87) | | | | | | | | | **2.65 (0.75,4.54)*** | | | | | | | | | | Usual care | | | | | | | |  | | | | | | |
| **4.79 (0.09,9.49)*** | | | | | | | | 2.00 (-4.36,8.36) | | | | | | | | | 2.87 (-2.53,8.28) | | | | | | | | | | -0.23 (-5.25,4.80) | | | | | | | | Usual rehabilitation | | | | | | |
| **Lower limb motor ability [MD (95%CI)]** | | | | | | | | | | | | | | | | | | | | | | | | | | | | | | | | | | | | | | | | | |
| Taiji | |  | | | | | | | | | | |  | | | | | | | | | |  | | | | | | | | | | | | | | | | |  | |
| 1.38 (-1.20,3.96) | | Baduanjin | | | | | | | | | | |  | | | | | | | | | |  | | | | | | | | | | | | | | | | |  | |
| 3.00 (-3.10,9.11) | | 1.62 (-3.92,7.16) | | | | | | | | | | | Daoyin | | | | | | | | | |  | | | | | | | | | | | | | | | | |  | |
| **3.94 (1.90,5.98)*** | | **2.56 (0.33,4.78)*** | | | | | | | | | | | 0.94 (-5.03,6.90) | | | | | | | | | | Usual rehabilitation | | | | | | | | | | | | | | | | |  | |
| **6.00 (1.35,10.66)*** | | **4.62 (0.75,8.49)*** | | | | | | | | | | | 3.00 (-0.96,6.96) | | | | | | | | | | 2.06 (-2.40,6.53) | | | | | | | | | | | | | | | | | Usual care | |
| **Overall motor ability [MD (95%CI)]** | | | | | | | | | | | | | | | | | | | | | | | | | | | | | | | | | | | | | | | | | |
| Baduanjin | | | | |  | | | | | |  | | | | | | | | |  | | | | | | | |  | | | | | | | |  | | | | | |
| 8.50 (-10.22,27.22) | | | | | Daoyin | | | | | |  | | | | | | | | |  | | | | | | | |  | | | | | | | |  | | | | | |
| 12.68 (-14.71,40.07) | | | | | 4.18 (-29.00,37.36) | | | | | | Liuzijue | | | | | | | | |  | | | | | | | |  | | | | | | | |  | | | | | |
| **21.01 (1.63,40.39)*** | | | | | 12.51 (-6.95,31.97) | | | | | | 8.33 (-25.23,41.88) | | | | | | | | | Taiji | | | | | | | |  | | | | | | | |  | | | | | |
| **20.69 (1.62,39.75)*** | | | | | 12.19 (-14.53,38.91) | | | | | | 8.01 (-11.67,27.69) | | | | | | | | | -0.32 (-27.51,26.87) | | | | | | | | Usual care | | | | | | | |  | | | | | |
| **22.31 (9.12,35.49)*** | | | | | **13.81 (0.51,27.11)*** | | | | | | 9.63 (-20.77,40.03) | | | | | | | | | 1.30 (-12.91,15.51) | | | | | | | | 1.62 (-21.56,24.80) | | | | | | | | Usual rehabilitation | | | | | |
| **Walking ability [SMD (95%CI)]** | | | | | | | | | | | | | | | | | | | | | | | | | | | | | | | | | | | | | | | | | |
| Baduanjin | | | | | | | |  | | | | | | | | | | |  | | | | | | | | | | | |  | | | | | | | | | | |
| 0.48 (-0.34,1.31) | | | | | | | | Taiji | | | | | | | | | | |  | | | | | | | | | | | |  | | | | | | | | | | |
| **1.04 (0.40,1.68)*** | | | | | | | | **0.55 (0.04,1.07)*** | | | | | | | | | | | Usual care | | | | | | | | | | | |  | | | | | | | | | | |
| **1.01 (0.01,2.07)*** | | | | | | | | 0.53 (-0.14,1.20) | | | | | | | | | | | -0.02 (-0.86,0.81) | | | | | | | | | | | | Usual rehabilitation | | | | | | | | | | |
| **Balance ability [SMD (95%CI)]** | | | | | | | | | | | | | | | | | | | | | | | | | | | | | | | | | | | | | | | | | |
| Wuqinxi |  | | | | | | | | |  | | | | | | | |  | | | | | | | |  | | | | | | |  | | | | | |  | | |
| 0.20 (-1.05,1.45) | Baduanjin | | | | | | | | |  | | | | | | | |  | | | | | | | |  | | | | | | |  | | | | | |  | | |
| 0.33 (-0.88,1.54) | 0.13 (-0.76,1.02) | | | | | | | | | Liuzijue | | | | | | | |  | | | | | | | |  | | | | | | |  | | | | | |  | | |
| 0.45 (-0.67,1.57) | 0.25 (-0.47,0.96) | | | | | | | | | 0.12 (-0.57,0.81) | | | | | | | | Taiji | | | | | | | |  | | | | | | |  | | | | | |  | | |
| 1.02 (-0.62,2.65) | 0.81 (-0.50,2.13) | | | | | | | | | 0.68 (-0.69,2.06) | | | | | | | | 0.57 (-0.65,1.78) | | | | | | | | Daoyin | | | | | | |  | | | | | |  | | |
| 1.11 (-0.13,2.35) | **0.91 (0.14,1.68)*** | | | | | | | | | 0.78 (-0.10,1.65) | | | | | | | | **0.66 (0.07,1.25)*** | | | | | | | | 0.09 (-0.97,1.16) | | | | | | | Usual care | | | | | |  | | |
| **1.10 (0.04,2.16)*** | **0.90 (0.23,1.57)*** | | | | | | | | | **0.77 (0.18,1.36)*** | | | | | | | | **0.65 (0.29,1.01)*** | | | | | | | | 0.08 (-1.16,1.33) | | | | | | | -0.01 (-0.66,0.64) | | | | | | Usual rehabilitation | | |
| **Self-care ability [SMD (95%CI)]** | | | | | | | | | | | | | | | | | | | | | | | | | | | | | | | | | | | | | | | | | |
| Baduanjin | | | |  | | | | | | | |  | | | | | | | | | |  | | | | | | | |  | | | | | | | |  | | | |
| -0.19 (-25.07,24.68) | | | | Daoyin | | | | | | | |  | | | | | | | | | |  | | | | | | | |  | | | | | | | |  | | | |
| 1.70 (-8.11,11.51) | | | | 1.89 (-20.97,24.75) | | | | | | | | Taiji | | | | | | | | | |  | | | | | | | |  | | | | | | | |  | | | |
| 2.08 (-8.69,12.86) | | | | 2.28 (-23.13,27.69) | | | | | | | | 0.39 (-10.72,11.50) | | | | | | | | | | Liuzijue | | | | | | | |  | | | | | | | |  | | | |
| 4.81 (-13.44,23.05) | | | | 5.00 (-11.91,21.91) | | | | | | | | 3.11 (-12.27,18.49) | | | | | | | | | | 2.72 (-16.25,21.69) | | | | | | | | Usual care | | | | | | | |  | | | |
| **8.08 (1.42,14.75)*** | | | | 8.28 (-15.69,32.24) | | | | | | | | 6.39 (-0.81,13.59) | | | | | | | | | | 6.00 (-2.46,14.45) | | | | | | | | 3.28 (-13.71,20.26) | | | | | | | | Usual rehabilitation | | | |
| **Cognitive functioning [MD (95%CI)]** | | | | | | | | | | | | | | | | | | | | | | | | | | | | | | | | | | | | | | | | | |
| Baduanjin | | | | | | |  | | | | | | | |  | | | | | | | | | |  | | | | | | | | |  | | | | | | | |
| 0.62 (-0.10,1.33) | | | | | | | Liuzijue | | | | | | | |  | | | | | | | | | |  | | | | | | | | |  | | | | | | | |
| 0.68 (-0.24,1.60) | | | | | | | 0.06 (-0.71,0.84) | | | | | | | | Taiji | | | | | | | | | |  | | | | | | | | |  | | | | | | | |
| **1.15 (0.54,1.77)*** | | | | | | | **0.53 (0.17,0.90)*** | | | | | | | | 0.47 (-0.21,1.16) | | | | | | | | | | Usual care | | | | | | | | |  | | | | | | | |
| **1.82 (1.38,2.26)*** | | | | | | | **1.21 (0.37,2.04)*** | | | | | | | | **1.14 (0.13,2.16)*** | | | | | | | | | | 0.67 (-0.08,1.43) | | | | | | | | | Usual rehabilitation | | | | | | | |
| **Depression [SMD (95%CI)]** | | | | | | | | | | | | | | | | | | | | | | | | | | | | | | | | | | | | | | | | | |
| Liuzijue |  | | | | | | | |  | | | | | | |  | | | | | | | | | |  | | | | | |  | | | | | | | | |  |
| -0.87 (-5.08,3.34) | Baduanjin | | | | | | | |  | | | | | | |  | | | | | | | | | |  | | | | | |  | | | | | | | | |  |
| -1.21 (-4.74,2.32) | -0.34 (-4.34,3.67) | | | | | | | | Yijinjing | | | | | | |  | | | | | | | | | |  | | | | | |  | | | | | | | | |  |
| -1.46 (-4.27,1.34) | -0.59 (-4.48,3.29) | | | | | | | | -0.25 (-3.39,2.88) | | | | | | | Taiji | | | | | | | | | |  | | | | | |  | | | | | | | | |  |
| -1.39 (-5.67,2.89) | -0.52 (-5.82,4.77) | | | | | | | | -0.18 (-4.96,4.59) | | | | | | | 0.07 (-3.70,3.84) | | | | | | | | | | Daoyin | | | | | |  | | | | | | | | |  |
| -2.30 (-4.96,0.36) | -1.43 (-4.69,1.84) | | | | | | | | -1.09 (-3.41,1.23) | | | | | | | -0.84 (-2.95,1.28) | | | | | | | | | | -0.91 (-5.08,3.27) | | | | | | Usual rehabilitation | | | | | | | | |  |
| **-3.51 (-6.22,-0.80)*** | -2.64 (-6.77,1.50) | | | | | | | | -2.30 (-5.74,1.14) | | | | | | | **-2.04 (-3.84,-0.25)*** | | | | | | | | | | -2.11 (-5.42,1.20) | | | | | | -1.21 (-3.75,1.33) | | | | | | | | | Usual care |
| **Quality of life [SMD (95%CI)]** | | | | | | | | | | | | | | | | | | | | | | | | | | | | | | | | | | | | | | | | | |
| Baduanjin | | | | | |  | | | | | | | |  | | | | | | | | | |  | | | | | | | | |  | | | | | | | | |
| 0.22 (-2.34,2.79) | | | | | | Wuqinxi | | | | | | | |  | | | | | | | | | |  | | | | | | | | |  | | | | | | | | |
| 1.04 (-0.40,2.48) | | | | | | 0.82 (-1.74,3.38) | | | | | | | | Taiji | | | | | | | | | |  | | | | | | | | |  | | | | | | | | |
| **1.52 (0.20,2.84)*** | | | | | | 1.30 (-1.33,3.93) | | | | | | | | 0.48 (-0.54,1.49) | | | | | | | | | | Usual care | | | | | | | | |  | | | | | | | | |
| **1.88 (0.57,3.20)*** | | | | | | 1.66 (-0.54,3.86) | | | | | | | | 0.84 (-0.47,2.15) | | | | | | | | | | 0.36 (-1.07,1.80) | | | | | | | | | Usual rehabilitation | | | | | | | | |
| **Quality of sleep [SMD (95%CI)]** | | | | | | | | | | | | | | | | | | | | | | | | | | | | | | | | | | | | | | | | | |
| Baduanjin | | |  | | | | | | |  | | | | | | | | | | |  | | | | | | | |  | | | | | | | |  | | | | |
| -3.00 (-6.74,0.74) | | | Daoyin | | | | | | |  | | | | | | | | | | |  | | | | | | | |  | | | | | | | |  | | | | |
| -3.16 (-7.64,1.31) | | | -0.16 (-4.28,3.96) | | | | | | | Wuqinxi | | | | | | | | | | |  | | | | | | | |  | | | | | | | |  | | | | |
| **-3.32 (-6.56,-0.08)*** | | | -0.32 (-3.05,2.41) | | | | | | | -0.16 (-3.25,2.93) | | | | | | | | | | | Taiji | | | | | | | |  | | | | | | | |  | | | | |
| **-3.90 (-6.81,-0.98)*** | | | -0.90 (-3.24,1.44) | | | | | | | -0.74 (-4.13,2.66) | | | | | | | | | | | -0.58 (-1.99,0.83) | | | | | | | | Usual care | | | | | | | |  | | | | |
| **-5.16 (-9.46,-0.86)*** | | | -2.16 (-6.09,1.77) | | | | | | | **-2.00 (-3.23,-0.77)*** | | | | | | | | | | | -1.84 (-4.67,0.99) | | | | | | | | -1.26 (-4.43,1.90) | | | | | | | | Usual rehabilitation | | | | |

1. Global inconsistency test

The Global inconsistency test assumes that for a given treatment comparison, the results from direct and indirect comparisons should be consistent. In other words, for the same treatment comparison, the results from different research methods (direct and indirect) should not have systematic differences. The Global inconsistency test is conducted by constructing a consistency model and an inconsistency model. The consistency model assumes that all direct and indirect comparison results are consistent, while the inconsistency model allows for systematic differences. A statistical test, typically using a chi-square test, compares the goodness-of-fit between the consistency and inconsistency models. If p <0.05, it indicates the presence of global inconsistency.

| Table S13 Global inconsistency test | |
| --- | --- |
| **Upper limb motor ability** | |
| Chi^2^ | p |
| 0.38 | >0.05 |
| **Lower limb motor ability** | |
| Chi^2^ | p |
| 34.25 | <0.001 |
| **Overall motor ability** | |
| Chi^2^ | p |
| 4.13 | >0.05 |
| **Walking ability** | |
| Chi^2^ | p |
| 2.60 | >0.05 |
| **Balance ability** | |
| Chi^2^ | p |
| 1.20 | >0.05 |
| **Self-care ability** | |
| Chi^2^ | p |
| 3.03 | >0.05 |
| **Cognitive functioning** | |
| Chi^2^ | p |
| 1.84 | >0.05 |
| **Depression** | |
| Chi^2^ | p |
| 3.33 | >0.05 |
| **Quality of life** | |
| Chi^2^ | p |
| 0.30 | >0.05 |
| **Quality of sleep** | |
| Chi^2^ | p |
| 0.57 | >0.05 |

1. Local inconsistency test

Using the node-splitting method for inconsistency testing is applicable only when there are closed loops within the network. Local inconsistency testing involves splitting the nodes of a closed loop into direct comparisons, indirect comparisons, and mixed comparisons, then assessing the differences among these three. If p <0.05, it indicates the presence of local inconsistency.

| Table S14 Local inconsistency test | | | | | | | |
| --- | --- | --- | --- | --- | --- | --- | --- |
| **Upper limb motor ability** | | | | | | | |
| Side | Direct | | Indirect | | Difference | | P |
|  | Coef. | Std. Err. | Coef. | Std. Err. | Coef. | Std. Err. |  |
| A D * | -4.56 | 0.91 | -2.65 | 158.23 | -1.92 | 158.23 | >0.05 |
| A E * | -4.79 | 2.40 | -2.00 | 316.33 | -2.79 | 316.34 | >0.05 |
| B D * | -2.65 | 0.97 | -6.48 | 316.18 | 3.84 | 316.19 | >0.05 |
| C E * | -2.00 | 3.24 | -7.58 | 632.49 | 5.58 | 632.51 | >0.05 |
| **Lower limb motor ability** | | | | | | | |
| Side | Direct | | Indirect | | Difference | | P |
|  | Coef. | Std. Err. | Coef. | Std. Err. | Coef. | Std. Err. |  |
| A C | 2.04 | 1.77 | 0.28 | 2.32 | 1.76 | 2.92 | >0.05 |
| A D | -4.62 | 1.97 | -3 | 316.23 | -1.62 | 316.23 | >0.05 |
| A E | -2.46 | 1.35 | -3.74 | 4.44 | 1.27 | 4.64 | >0.05 |
| B D | -3 | 2.02 | -6.24 | 632.43 | 3.24 | 632.44 | >0.05 |
| C E | -3.17 | 0.9 | -10.13 | 2.08 | 6.96 | 2.27 | 0.02 |

| **Overall motor ability** | | | | | | | |
| --- | --- | --- | --- | --- | --- | --- | --- |
| Side | Direct | | Indirect | | Difference | | P |
|  | Coef. | Std. Err. | Coef. | Std. Err. | Coef. | Std. Err. |  |
| A E * | -20.70 | 9.74 | -8.00 | 316.39 | -12.70 | 316.54 | >0.05 |
| A F * | -22.34 | 6.74 | -7.55 | 158.19 | -14.79 | 158.33 | >0.05 |
| B E * | -8.00 | 10.05 | -33.40 | 632.98 | 25.40 | 633.08 | >0.05 |
| C F * | -1.29 | 7.25 | -43.37 | 447.58 | 42.08 | 447.65 | >0.05 |
| D F * | -13.81 | 6.79 | -30.83 | 447.57 | 17.02 | 447.63 | >0.05 |
| **Walking ability** | | | | | | | |
| Side | Direct | | Indirect | | Difference | | P |
|  | Coef. | Std. Err. | Coef. | Std. Err. | Coef. | Std. Err. |  |
| A C * | -1.04 | 0.30 | -0.54 | 105.27 | -0.50 | 105.27 | >0.05 |
| B C * | -0.54 | 0.24 | -1.16 | 138.34 | 0.62 | 138.34 | >0.05 |
| B D * | -0.43 | 0.27 | 0.58 | 313.99 | -1.01 | 313.99 | >0.05 |
| **Balance ability** | | | | | | | |
| Side | Direct | | Indirect | | Difference | | P |
|  | Coef. | Std. Err. | Coef. | Std. Err. | Coef. | Std. Err. |  |
| A E | -1.35 | 0.56 | -0.49 | 0.54 | -0.85 | 0.78 | >0.05 |
| A F | -0.68 | 0.39 | -1.54 | 0.67 | 0.85 | 0.78 | >0.05 |
| B E * | -0.09 | 0.54 | -1.72 | 633.23 | 1.63 | 633.23 | >0.05 |
| C F * | -0.77 | 0.30 | -1.03 | 366.98 | 0.26 | 366.98 | >0.05 |
| D E | -0.51 | 0.33 | -1.36 | 0.71 | 0.85 | 0.78 | >0.05 |
| D F | -0.70 | 0.19 | 0.15 | 0.76 | -0.85 | 0.78 | >0.05 |
| F G * | 1.10 | 0.54 | 2.90 | 635.44 | -1.80 | 635.44 | >0.05 |
| **Self-care ability** | | | | | | | |
| Side | Direct | | Indirect | | Difference | | P |
|  | Coef. | Std. Err. | Coef. | Std. Err. | Coef. | Std. Err. |  |
| A F * | -8.08 | 3.40 | -6.47 | 105.45 | -1.62 | 105.51 | >0.05 |
| B E * | -5.00 | 8.62 | -4.61 | 632.86 | -0.39 | 632.96 | >0.05 |
| C F * | -6.00 | 4.31 | -10.17 | 365.28 | 4.17 | 365.32 | >0.05 |
| D E * | -3.11 | 7.85 | -2.59 | 283.00 | -0.52 | 283.10 | >0.05 |
| D F * | -6.39 | 3.67 | -8.02 | 182.72 | 1.63 | 182.75 | >0.05 |
| **Cognitive functioning** | | | | | | | |
| Side | Direct | | Indirect | | Difference | | P |
|  | Coef. | Std. Err. | Coef. | Std. Err. | Coef. | Std. Err. |  |
| A D * | -1.15 | 0.31 | -0.51 | 182.27 | -0.64 | 182.27 | >0.05 |
| A E | - | - | - | - | - | - | - |
| B D * | -0.53 | 0.19 | -1.77 | 439.25 | 1.24 | 439.25 | >0.05 |
| C D * | -0.47 | 0.35 | -1.83 | 641.99 | 1.36 | 641.99 | >0.05 |
| **Depression** | | | | | | | |
| Side | Direct | | Indirect | | Difference | | P |
|  | Coef. | Std. Err. | Coef. | Std. Err. | Coef. | Std. Err. |  |
| A F * | 1.43 | 1.67 | 0.97 | 100.00 | 0.46 | 100.01 | >0.05 |
| B E * | 2.11 | 1.69 | 3.16 | 632.29 | -1.05 | 632.29 | >0.05 |
| C E | 5.11 | 1.46 | 0.78 | 1.87 | 4.33 | 2.37 | >0.05 |
| C F | 0.81 | 1.38 | 5.14 | 1.93 | -4.33 | 2.37 | >0.05 |
| D E | 1.53 | 0.81 | 5.86 | 2.23 | -4.33 | 2.37 | >0.05 |
| D F | 1.56 | 0.97 | -2.76 | 2.16 | 4.33 | 2.37 | >0.05 |
| F G * | -1.09 | 1.18 | -3.95 | 447.07 | 2.86 | 447.07 | >0.05 |
| **Quality of life** | | | | | | | |
| Side | Direct | | Indirect | | Difference | | P |
|  | Coef. | Std. Err. | Coef. | Std. Err. | Coef. | Std. Err. |  |
| A C | -1.27 | 0.84 | -2.14 | 1.33 | 0.86 | 1.57 | >0.05 |
| A D | -2.14 | 0.84 | -1.27 | 1.33 | -0.86 | 1.57 | >0.05 |
| B C | -0.60 | 0.59 | 0.27 | 1.46 | -0.86 | 1.57 | >0.05 |
| B D | -0.60 | 0.84 | -1.46 | 1.33 | 0.86 | 1.57 | >0.05 |
| D E * | 1.66 | 1.12 | 5.43 | 633.33 | -3.77 | 633.33 | >0.05 |
| **Quality of sleep** | | | | | | | |
| Side | Direct | | Indirect | | Difference | | P |
|  | Coef. | Std. Err. | Coef. | Std. Err. | Coef. | Std. Err. |  |
| A C | 2.04 | 1.77 | 0.28 | 2.32 | 1.76 | 2.92 | >0.05 |
| A D * | -4.62 | 1.97 | -3.00 | 316.23 | -1.62 | 316.23 | >0.05 |
| A E * | -2.46 | 1.35 | -3.74 | 4.44 | 1.27 | 4.64 | >0.05 |
| B D * | -3.00 | 2.02 | -6.24 | 632.43 | 3.24 | 632.44 | >0.05 |
| C E * | -3.17 | 0.90 | -10.13 | 2.08 | 6.96 | 2.27 | >0.05 |

1. Heterogeneity test

Prediction intervals are based on the actual intervals (confidence intervals) and are extended by accounting for heterogeneity. If the prediction intervals and actual intervals are statistically consistent, this indicates that the impact of heterogeneity on the results is minimal, meaning that differences between studies do not significantly alter the conclusions.

| Table S15 heterogeneity test | | | | | | |
| --- | --- | --- | --- | --- | --- | --- |
| **Upper limb motor ability** | | | | | | |
| Comparison | Effect size | LCI | UCI | LPrI | UPrI | Heterogeneity |
| Taiji vs Baduanjin | -1.92 | -4.59 | 0.75 | -6.9 | 3.07 | Not serious |
| Daoyin vs Baduanjin | -2.79 | -10.69 | 5.11 | -15.85 | 10.27 | Not serious |
| Usual rehabilitation vs Baduanjin | -4.56 | -6.35 | -2.78 | -8.36 | -0.77 | Not serious |
| Usual care vs Baduanjin | -4.79 | -9.49 | -0.09 | -12.8 | 3.22 | Serious |
| Daoyin vs Taiji | -0.87 | -9.21 | 7.47 | -14.64 | 12.89 | Not serious |
| Usual rehabilitation vs Taiji | -2.65 | -4.54 | -0.75 | -6.58 | 1.29 | Serious |
| Usual care vs Taiji | -2.87 | -8.27 | 2.53 | -11.98 | 6.24 | Not serious |
| Usual rehabilitation vs Daoyin | -1.77 | -9.87 | 6.33 | -15.15 | 11.61 | Not serious |
| Usual care vs Daoyin | -2 | -8.36 | 4.35 | -12.61 | 8.61 | Not serious |
| Usual care vs Usual rehabilitation | -0.23 | -5.25 | 4.8 | -8.74 | 8.29 | Not serious |
| **Lower limb motor ability** | | | | | | |
| Comparison | Effect size | LCI | UCI | LPrI | UPrI | Heterogeneity |
| Daoyin vs Baduanjin | -1.62 | -7.16 | 3.92 | -42.94 | 39.7 | Not serious |
| Taiji vs Baduanjin | 1.38 | -1.2 | 3.96 | -25.06 | 27.83 | Not serious |
| Usual care vs Baduanjin | -4.62 | -8.49 | -0.75 | -37.01 | 27.77 | Serious |
| Usual rehabilitation vs Baduanjin | -2.56 | -4.78 | -0.33 | -27.61 | 22.49 | Serious |
| Taiji vs Daoyin | 3 | -3.1 | 9.11 | -41.57 | 47.58 | Not serious |
| Usual care vs Daoyin | -3 | -6.96 | 0.96 | -35.83 | 29.82 | Not serious |
| Usual rehabilitation vs Daoyin | -0.94 | -6.9 | 5.03 | -44.7 | 42.83 | Not serious |
| Usual care vs Taiji | -6 | -10.66 | -1.35 | -42.46 | 30.45 | Serious |
| Usual rehabilitation vs Taiji | -3.94 | -5.98 | -1.9 | -28.32 | 20.44 | Serious |
| Usual rehabilitation vs Usual care | 2.06 | -2.4 | 6.53 | -33.39 | 37.52 | Not serious |
| Daoyin vs Baduanjin | -1.62 | -7.16 | 3.92 | -42.94 | 39.7 | Not serious |
| **Overall motor ability** | | | | | | |
| Comparison | Effect size | LCI | UCI | LPrI | UPrI | Heterogeneity |
| Daoyin vs Baduanjin | -12.68 | -40.07 | 14.71 | -85.29 | 59.93 | Not serious |
| Liuzijue vs Baduanjin | -21.01 | -40.39 | -1.63 | -79.88 | 37.87 | Serious |
| Taiji vs Baduanjin | -8.50 | -27.22 | 10.22 | -66.34 | 49.34 | Not serious |
| Usual care vs Baduanjin | -20.69 | -39.75 | -1.62 | -79.07 | 37.69 | Serious |
| Usual rehabilitation vs Baduanjin | -22.31 | -35.49 | -9.12 | -72.25 | 27.63 | Serious |
| Liuzijue vs Daoyin | -8.33 | -41.88 | 25.23 | -92.49 | 75.83 | Not serious |
| Taiji vs Daoyin | 4.18 | -29.00 | 37.36 | -79.26 | 87.62 | Not serious |
| Usual care vs Daoyin | -8.01 | -27.69 | 11.67 | -67.36 | 51.35 | Not serious |
| Usual rehabilitation vs Daoyin | -9.63 | -40.03 | 20.77 | -87.80 | 68.54 | Not serious |
| Taiji vs Liuzijue | 12.51 | -6.95 | 31.97 | -46.50 | 71.51 | Not serious |
| Usual care vs Liuzijue | 0.32 | -26.87 | 27.51 | -71.91 | 72.56 | Not serious |
| Usual rehabilitation vs Liuzijue | -1.30 | -15.51 | 12.91 | -52.58 | 49.98 | Not serious |
| Usual care vs Taiji | -12.19 | -38.91 | 14.53 | -83.58 | 59.21 | Not serious |
| Usual rehabilitation vs Taiji | -13.81 | -27.11 | -0.51 | -63.89 | 36.28 | Serious |
| Usual rehabilitation vs Usual care | -1.62 | -24.80 | 21.56 | -66.78 | 63.54 | Not serious |
| **Walking ability** | | | | | | |
| Comparison | Effect size | LCI | UCI | LPrI | UPrI | Heterogeneity |
| Taiji vs Baduanjin | -0.50 | -1.26 | 0.26 | -1.86 | 0.85 | Not serious |
| Usual care vs Baduanjin | -1.04 | -1.63 | -0.45 | -2.27 | 0.20 | Serious |
| Usual rehabilitation vs Baduanjin | -0.93 | -1.84 | -0.02 | -2.41 | 0.55 | Serious |
| Usual care vs Taiji | -0.54 | -1.01 | -0.06 | -1.70 | 0.63 | Serious |
| Usual rehabilitation vs Taiji | -0.43 | -0.95 | 0.09 | -1.62 | 0.76 | Not serious |
| Usual rehabilitation vs Usual care | 0.11 | -0.59 | 0.80 | -1.20 | 1.41 | Not serious |
| **Balance ability** | | | | | | |
| Comparison | Effect size | LCI | UCI | LPrI | UPrI | Heterogeneity |
| Daoyin vs Baduanjin | -0.81 | -2.13 | 0.50 | -2.59 | 0.96 | Not serious |
| Liuzijue vs Baduanjin | -0.13 | -1.02 | 0.76 | -1.54 | 1.28 | Not serious |
| Taiji vs Baduanjin | -0.25 | -0.96 | 0.47 | -1.53 | 1.03 | Not serious |
| Usual care vs Baduanjin | -0.91 | -1.68 | -0.14 | -2.23 | 0.41 | Serious |
| Usual rehabilitation vs Baduanjin | -0.90 | -1.57 | -0.23 | -2.15 | 0.35 | Serious |
| Wuqinxi vs Baduanjin | 0.20 | -1.05 | 1.45 | -1.52 | 1.92 | Not serious |
| Liuzijue vs Daoyin | 0.68 | -0.69 | 2.06 | -1.15 | 2.52 | Not serious |
| Taiji vs Daoyin | 0.57 | -0.65 | 1.78 | -1.12 | 2.25 | Not serious |
| Usual care vs Daoyin | -0.09 | -1.16 | 0.97 | -1.65 | 1.46 | Not serious |
| Usual rehabilitation vs Daoyin | -0.08 | -1.33 | 1.16 | -1.80 | 1.63 | Not serious |
| Wuqinxi vs Daoyin | 1.02 | -0.62 | 2.65 | -1.06 | 3.09 | Not serious |
| Taiji vs Liuzijue | -0.12 | -0.81 | 0.57 | -1.38 | 1.15 | Not serious |
| Usual care vs Liuzijue | -0.78 | -1.65 | 0.10 | -2.18 | 0.62 | Not serious |
| Usual rehabilitation vs Liuzijue | -0.77 | -1.36 | -0.18 | -1.97 | 0.43 | Serious |
| Wuqinxi vs Liuzijue | 0.33 | -0.88 | 1.54 | -1.35 | 2.01 | Not serious |
| Usual care vs Taiji | -0.66 | -1.25 | -0.07 | -1.86 | 0.54 | Serious |
| Usual rehabilitation vs Taiji | -0.65 | -1.01 | -0.29 | -1.74 | 0.43 | Serious |
| Wuqinxi vs Taiji | 0.45 | -0.67 | 1.57 | -1.15 | 2.05 | Not serious |
| Usual rehabilitation vs Usual care | 0.01 | -0.64 | 0.66 | -1.23 | 1.25 | Not serious |
| Wuqinxi vs Usual care | 1.11 | -0.13 | 2.35 | -0.60 | 2.82 | Not serious |
| Wuqinxi vs Usual rehabilitation | 1.10 | 0.04 | 2.16 | -0.45 | 2.65 | Not serious |
| **Self-care ability** | | | | | | |
| Comparison | Effect size | LCI | UCI | LPrI | UPrI | Heterogeneity |
| Daoyin vs Baduanjin | 0.19 | -24.68 | 25.07 | -33.29 | 33.68 | Not serious |
| Liuzijue vs Baduanjin | -2.08 | -12.86 | 8.69 | -22.71 | 18.54 | Not serious |
| Taiji vs Baduanjin | -1.70 | -11.51 | 8.11 | -21.64 | 18.25 | Not serious |
| Usual care vs Baduanjin | -4.81 | -23.05 | 13.44 | -31.74 | 22.13 | Not serious |
| Usual rehabilitation vs Baduanjin | -8.08 | -14.75 | -1.42 | -26.14 | 9.98 | Serious |
| Liuzijue vs Daoyin | -2.28 | -27.69 | 23.13 | -36.32 | 31.76 | Not serious |
| Taiji vs Daoyin | -1.89 | -24.75 | 20.97 | -33.32 | 29.54 | Not serious |
| Usual care vs Daoyin | -5.00 | -21.91 | 11.91 | -30.70 | 20.70 | Not serious |
| Usual rehabilitation vs Daoyin | -8.28 | -32.24 | 15.69 | -40.83 | 24.28 | Not serious |
| Taiji vs Liuzijue | 0.39 | -10.72 | 11.50 | -20.48 | 21.26 | Not serious |
| Usual care vs Liuzijue | -2.72 | -21.69 | 16.25 | -30.34 | 24.90 | Not serious |
| Usual rehabilitation vs Liuzijue | -6.00 | -14.45 | 2.46 | -25.07 | 13.07 | Not serious |
| Usual care vs Taiji | -3.11 | -18.49 | 12.27 | -27.45 | 21.23 | Not serious |
| Usual rehabilitation vs Taiji | -6.39 | -13.59 | 0.81 | -24.73 | 11.95 | Not serious |
| Usual rehabilitation vs Usual care | -3.28 | -20.26 | 13.71 | -29.05 | 22.49 | Not serious |
| **Cognitive functioning** | | | | | | |
| Comparison | Effect size | LCI | UCI | LPrI | UPrI | Heterogeneity |
| Liuzijue vs Baduanjin | -0.62 | -1.33 | 0.10 | - | - | Not serious |
| Taiji vs Baduanjin | -0.68 | -1.60 | 0.24 | - | - | Not serious |
| Usual care vs Baduanjin | -1.15 | -1.77 | -0.54 | - | - | Not serious |
| Usual rehabilitation vs Baduanjin | -1.82 | -2.26 | -1.38 | - | - | Not serious |
| Taiji vs Liuzijue | -0.06 | -0.84 | 0.71 | - | - | Not serious |
| Usual care vs Liuzijue | -0.53 | -0.90 | -0.17 | - | - | Not serious |
| Usual rehabilitation vs Liuzijue | -1.21 | -2.04 | -0.37 | - | - | Not serious |
| Usual care vs Taiji | -0.47 | -1.16 | 0.21 | - | - | Not serious |
| Usual rehabilitation vs Taiji | -1.14 | -2.16 | -0.13 | - | - | Not serious |
| Usual rehabilitation vs Usual care | -0.67 | -1.43 | 0.08 | - | - | Not serious |
| Depression | | | | | | |
| Comparison | Effect size | LCI | UCI | LPrI | UPrI | Heterogeneity |
| Daoyin vs Baduanjin | 0.52 | -4.77 | 5.82 | -9.56 | 10.60 | Not serious |
| Liuzijue vs Baduanjin | -0.87 | -5.08 | 3.34 | -9.50 | 7.76 | Not serious |
| Taiji vs Baduanjin | 0.59 | -3.29 | 4.48 | -7.62 | 8.81 | Not serious |
| Usual care vs Baduanjin | 2.64 | -1.50 | 6.77 | -5.89 | 11.16 | Not serious |
| Usual rehabilitation vs Baduanjin | 1.43 | -1.84 | 4.69 | -6.04 | 8.89 | Not serious |
| Yijinjing vs Baduanjin | 0.34 | -3.67 | 4.34 | -8.02 | 8.70 | Not serious |
| Liuzijue vs Daoyin | -1.39 | -5.67 | 2.89 | -10.10 | 7.32 | Not serious |
| Taiji vs Daoyin | 0.07 | -3.70 | 3.84 | -8.00 | 8.14 | Not serious |
| Usual care vs Daoyin | 2.11 | -1.20 | 5.42 | -5.40 | 9.63 | Not serious |
| Usual rehabilitation vs Daoyin | 0.91 | -3.27 | 5.08 | -7.67 | 9.48 | Not serious |
| Yijinjing vs Daoyin | -0.18 | -4.96 | 4.59 | -9.55 | 9.18 | Not serious |
| Taiji vs Liuzijue | 1.46 | -1.34 | 4.27 | -5.49 | 8.42 | Not serious |
| Usual care vs Liuzijue | 3.51 | 0.80 | 6.22 | -3.35 | 10.36 | Serious |
| Usual rehabilitation vs Liuzijue | 2.30 | -0.36 | 4.96 | -4.51 | 9.10 | Not serious |
| Yijinjing vs Liuzijue | 1.21 | -2.32 | 4.74 | -6.57 | 8.99 | Not serious |
| Usual care vs Taiji | 2.04 | 0.25 | 3.84 | -3.97 | 8.05 | Serious |
| Usual rehabilitation vs Taiji | 0.84 | -1.28 | 2.95 | -5.44 | 7.11 | Not serious |
| Yijinjing vs Taiji | -0.25 | -3.39 | 2.88 | -7.57 | 7.06 | Not serious |
| Usual rehabilitation vs Usual care | -1.21 | -3.75 | 1.33 | -7.88 | 5.47 | Not serious |
| Yijinjing vs Usual care | -2.30 | -5.74 | 1.14 | -9.96 | 5.37 | Not serious |
| Yijinjing vs Usual rehabilitation | -1.09 | -3.41 | 1.23 | -7.56 | 5.38 | Not serious |
| **Quality of life** | | | | | | |
| Comparison | Effect size | LCI | UCI | LPrI | UPrI | Heterogeneity |
| Taiji vs Baduanjin | -1.04 | -2.48 | 0.40 | -4.39 | 2.30 | Not serious |
| Usual care vs Baduanjin | -1.52 | -2.84 | -0.20 | -4.77 | 1.74 | Serious |
| Usual rehabilitation vs Baduanjin | -1.88 | -3.20 | -0.57 | -5.14 | 1.37 | Serious |
| Wuqinxi vs Baduanjin | -0.22 | -2.79 | 2.34 | -4.57 | 4.13 | Not serious |
| Usual care vs Taiji | -0.48 | -1.49 | 0.54 | -3.54 | 2.59 | Not serious |
| Usual rehabilitation vs Taiji | -0.84 | -2.15 | 0.47 | -4.09 | 2.41 | Not serious |
| Wuqinxi vs Taiji | 0.82 | -1.74 | 3.38 | -3.52 | 5.17 | Not serious |
| Usual rehabilitation vs Usual care | -0.36 | -1.80 | 1.07 | -3.71 | 2.98 | Not serious |
| Wuqinxi vs Usual care | 1.30 | -1.33 | 3.93 | -3.12 | 5.71 | Not serious |
| Wuqinxi vs Usual rehabilitation | 1.66 | -0.54 | 3.86 | -2.33 | 5.66 | Not serious |
| **Quality of sleep** | | | | | | |
| Comparison | Effect size | LCI | UCI | LPrI | UPrI | Heterogeneity |
| Daoyin vs Baduanjin | 3.00 | -0.74 | 6.74 | - | - | Not serious |
| Taiji vs Baduanjin | 3.32 | 0.08 | 6.56 | - | - | Not serious |
| Usual care vs Baduanjin | 3.90 | 0.98 | 6.81 | - | - | Not serious |
| Usual rehabilitation vs Baduanjin | 5.16 | 0.86 | 9.46 | - | - | Not serious |
| Wuqinxi vs Baduanjin | 3.16 | -1.31 | 7.64 | - | - | Not serious |
| Taiji vs Daoyin | 0.32 | -2.41 | 3.05 | - | - | Not serious |
| Usual care vs Daoyin | 0.90 | -1.44 | 3.24 | - | - | Not serious |
| Usual rehabilitation vs Daoyin | 2.16 | -1.77 | 6.09 | - | - | Not serious |
| Wuqinxi vs Daoyin | 0.16 | -3.96 | 4.28 | - | - | Not serious |
| Usual care vs Taiji | 0.58 | -0.83 | 1.99 | - | - | Not serious |
| Usual rehabilitation vs Taiji | 1.84 | -0.99 | 4.67 | - | - | Not serious |
| Wuqinxi vs Taiji | -0.16 | -3.25 | 2.93 | - | - | Not serious |
| Usual rehabilitation vs Usual care | 1.26 | -1.90 | 4.43 | - | - | Not serious |
| Wuqinxi vs Usual care | -0.74 | -4.13 | 2.66 | - | - | Not serious |
| Wuqinxi vs Usual rehabilitation | -2.00 | -3.23 | -0.77 | - | - | Not serious |

1. Transitivity test


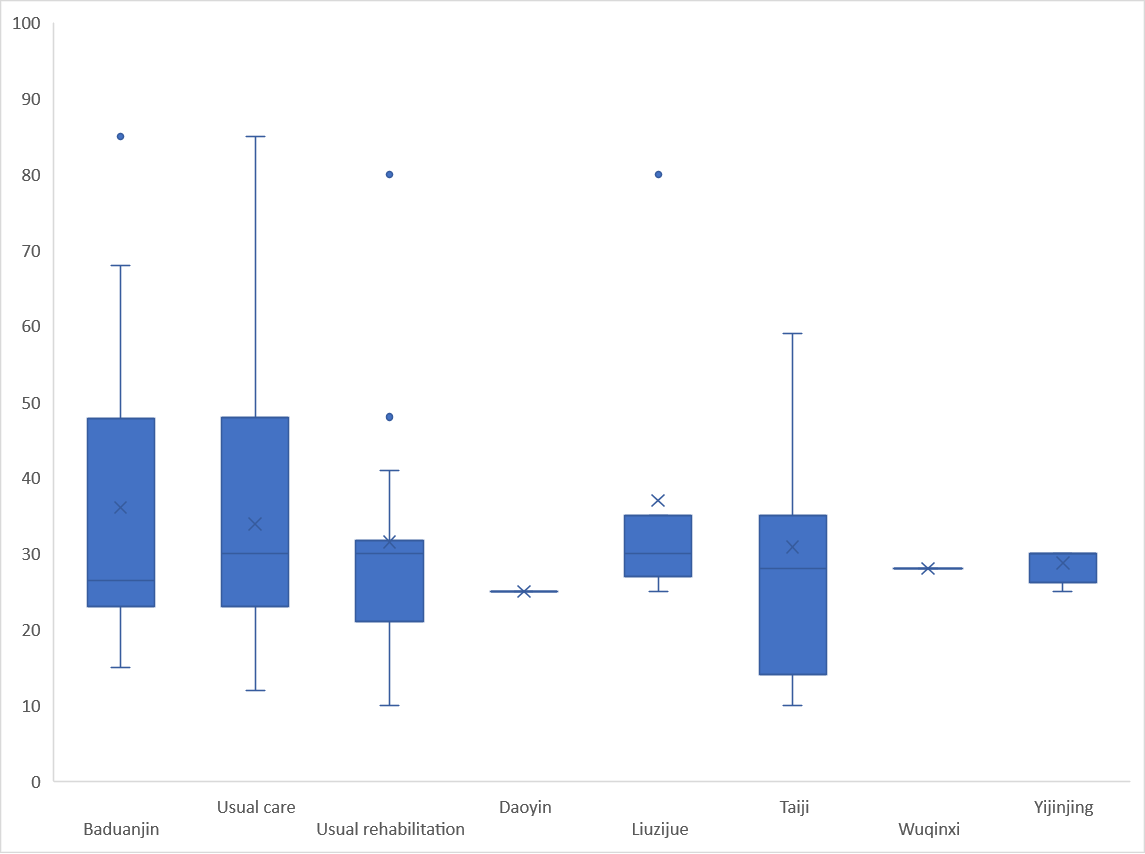


Figure S16 Transitivity test (Box Plot) for Sample size(n)


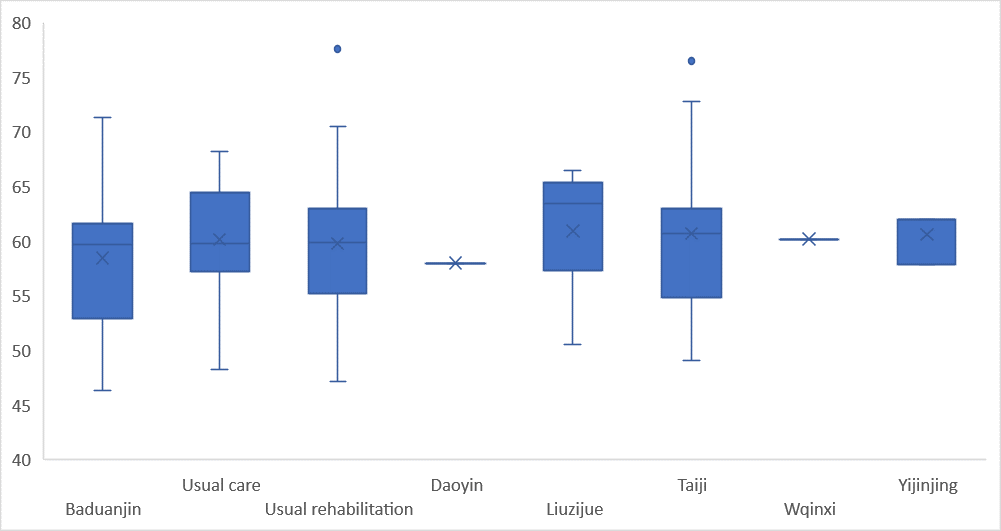


Figure S17 Transitivity test (Box Plot) for age(year)


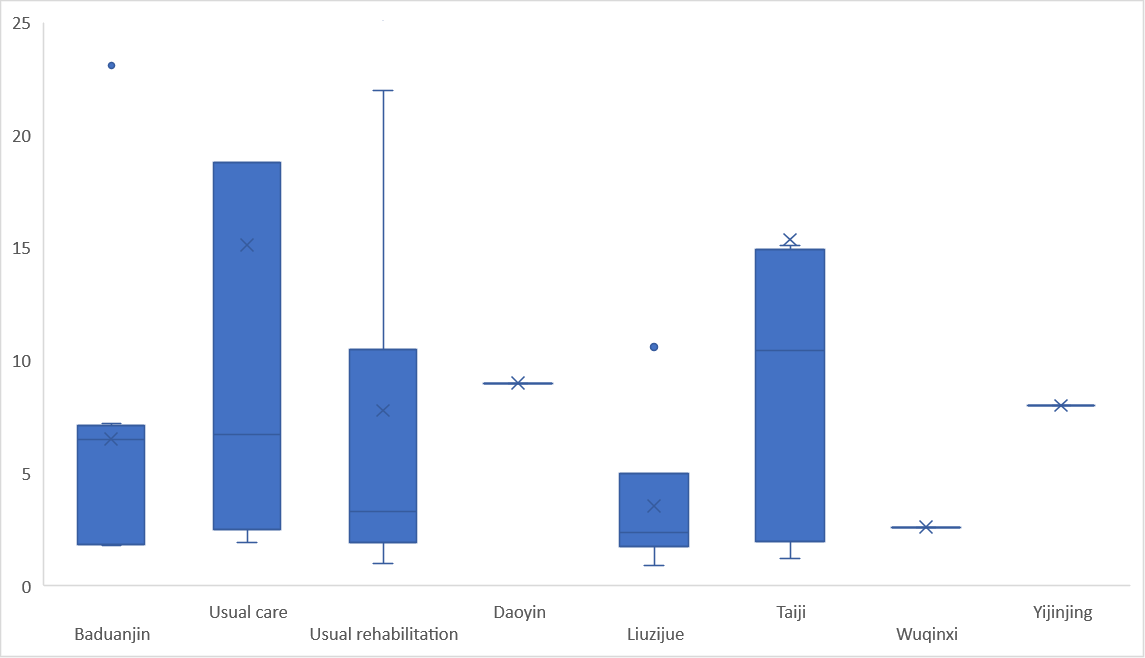


Figure S18 Transitivity test (Box Plot) for Disease duration(w)


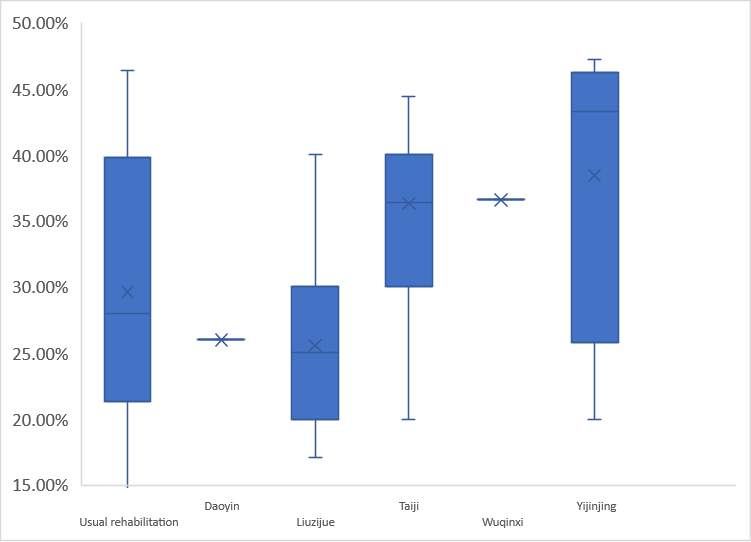


Figure S19 Transitivity test (Box Plot) for Gender( female%)


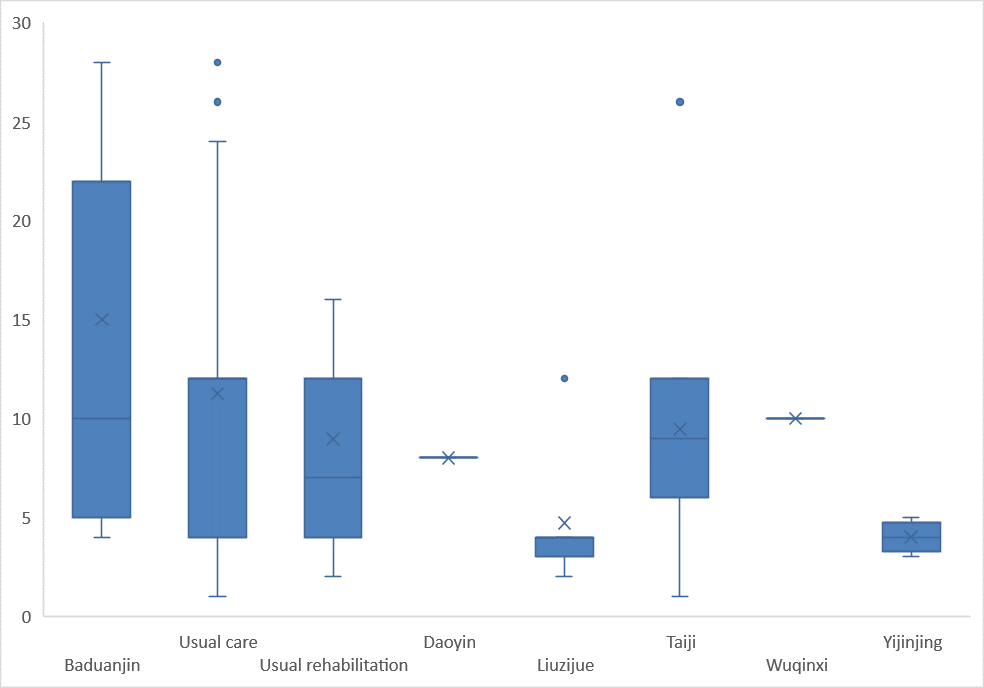


**Figure S20 Transitivity test (Box Plot) for Duration of intervention(w)**


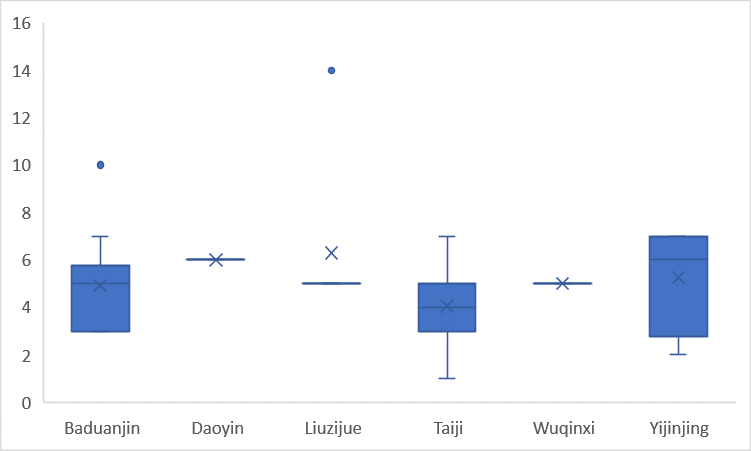


**Figure S21 Transitivity test (Box Plot) for Intervention intensity(times/w)**

**
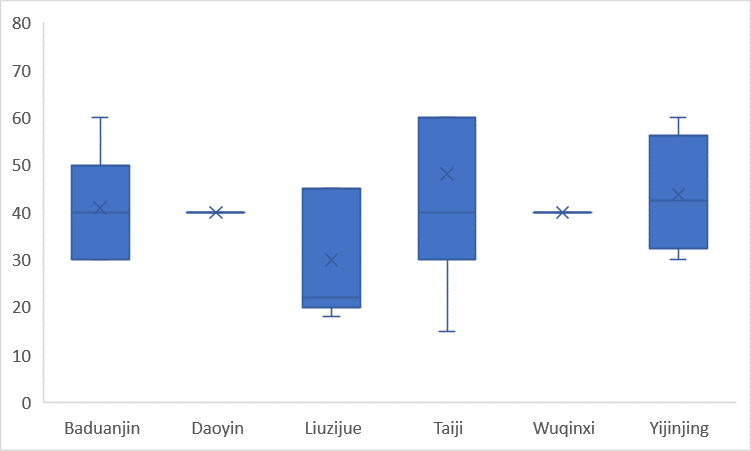
**

**Figure S22 Transitivity test (Box Plot) for Intervention period(min/times)**

1. Transitivity testReference list of included studies
2. Au-Yeung, S. S., Hui-Chan, C. W. & Tang, J. C. Short-form Tai Chi improves standing balance of people with chronic stroke. *Neurorehabil Neural Repair,* **23**, 515–522 (2009).
3. Chen, C. H., Hung, K. S., Chung, Y. C. & Yeh, M. L. Mind-body interactive qigong improves physical and mental aspects of quality of life in inpatients with stroke: a randomized control study. *Eur J Cardiovasc Nurs*. **18**, 658–666 (2019).
4. Ding, Y., Liu, Y., Xu, H., Huang, W., Yang, M. J., Cai, F., et al. Intervention with baduanjin can improve motor function and cognitive function in patients with apoplectic hemiplegia. *Int J Clin Exp Med,* **13**, 7544–7551 (2020).
5. Haolin, T., Yuanbin, Y., Hu, Z., Wenjing, Z., Jing, Z., Jingfeng, T., et al. Efficacy of Daoyin combined with lower limb robot as a comprehensive rehabilitation intervention for stroke patients: a randomized controlled trial. *J Tradit Chin Med,* **44**, 530–536 (2024).
6. Huang, S., Yu, X., Lu, Y., Qiao, J., Wang, H., Jiang, L. M., et al. Body weight support-Tai Chi footwork for balance of stroke survivors with fear of falling: a pilot randomized controlled trial. *Complement Ther Clin Pract*. **37**, 140–147 (2019).
7. Kim, H., Kim, Y. L. & Lee, S. M. Effects of therapeutic Tai Chi on balance gait and quality of life in chronic stroke patients. *Int J Rehabil Res,* **38**, 156–161 (2015).
8. Ku, P. H., Chen, S. F., Yang, Y. R., Lai, T. C. & Wang, R. Y. The effects of Ai Chi for balance in individuals with chronic stroke: a randomized controlled trial. **Sci Rep,** **10**, 1201 (2020).
9. Liu, Y., Chen, C., Du, H., Xue, M. & Zhu, N. Impact of Baduanjin exercise combined with rational emotive behavior therapy on sleep and mood in patients with poststroke depression: a randomized controlled trial. *Medicine,* **103**, e38180 (2024).
10. Lv, W., Wang, X., Liu, J. & Yu, P. Eight-Section Brocade Exercises improve the sleep quality, memory consolidation, and cardiopulmonary function of older adults with atrial fibrillation-associated stroke. Front. Psychol. 10, 2348 (2019).
11. Song, R., Park, M., Jang, T., Oh, J. & Sohn, M. K. Effects of a Tai Chi-based stroke rehabilitation program on symptom clusters physical and cognitive functions and quality of life: a randomized feasibility study. *Int J Environ Res Public Health,* **18**, 10 (2021).
12. Sun, P., Zhang, S., Jiang, L., Ma, Z., Yao, C., Zhu, Q., et al. Yijinjing Qigong intervention shows strong evidence on clinical effectiveness and electroencephalography signal features for early poststroke depression: a randomized controlled trial. *Front. Aging Neurosci.* **14**, 956316 (2022).
13. Taylor-Piliae, R. E. & Coull, B. M. Community-based Yang-style Tai Chi is safe and feasible in chronic stroke: a pilot study. *Clin. Rehabil,* **26**, 121–131 (2012).
14. Taylor-Piliae, R. E., Hoke, T. M., Hepworth, J. T., Latt, L. D., Najafi, B. & Coull, B. M. Effect of Tai Chi on physical function, fall rates and quality of life among older stroke survivors. *Arch. Phys Med Rehabil,* **95**, 816–824 (2014).
15. Wang, W., Sawada, M., Noriyama, Y., Arita, K., Ota, T., Sadamatsu, M., et al. Tai Chi exercise versus rehabilitation for the elderly with cerebral vascular disorder: a single-blinded randomized controlled trial. *Psychogeriatrics,* **10**, 160–166 (2010).
16. Xia, J., Pei, S., Chen, Z., Wang, L., Hu, J. & Wang, J. Effects of conventional speech therapy with Liuzijue Qigong a traditional Chinese method of breath training in 70 patients with post-stroke spastic dysarthria. *Med Sci Monit,* **29**, e939623 (2023).
17. Xie, G., Rao, T., Lin, L., Lin, Z., Xiao, T., Yang, M., et al. Effects of Tai Chi Yunshou exercise on community-based stroke patients: a cluster randomized controlled trial. *Eur Rev Aging Phys* *Act,* **15**, 17 (2018).
18. Yang, H., Li, B., Feng, L., Zhang, Z. & Liu, X. Effects of health Qigong exercise on upper extremity muscle activity balance function and quality of life in stroke patients. *Front. Neurosci,* **17**, 1208554 (2023).
19. Ye, M., Zheng, Y., Xiong, Z., Ye, B. & Zheng, G. Baduanjin exercise ameliorates motor function in patients with post-stroke cognitive impairment: a randomized controlled trial. *Complement Ther Clin Pract,* **46**, 101506 (2022).
20. Yu, X. M., Jin, X. M., Lu, Y., Gao, Y., Xu, H. C., Xue, X., et al. Effects of Body Weight Support-Tai Chi footwork training on balance control and walking function in stroke survivors with hemiplegia: a pilot randomized controlled trial. *Evid-Based Complement Alternat Med,* **2020**, 9218078 (2020).
21. Yuen, M., Ouyang, H. X., Miller, T. & Pang, M. Y. C. Baduanjin Qigong improves balance leg strength and mobility in individuals with chronic stroke: a randomized controlled study. *Neurorehabil Neural Repair,* **35**, 444–456 (2021).
22. Zhang, Y., Wang, C., Yang, J., Qiao, L., Xu, Y., Yu, L., et al. Comparing the effects of short-term Liuzijue exercise and core stability training on balance function in patients recovering from stroke: a pilot randomized controlled trial. *Front Neurol*, **13**, 748754 (2022).
23. Zheng, G., Zheng, Y., Xiong, Z. & Ye, B. Effect of Baduanjin exercise on cognitive function in patients with post-stroke cognitive impairment: a randomized controlled trial. *Clin Rehabil,* **34**, 1028–1039 (2020).
24. Zheng, Y., Zhang, Y., Li, H., Qiao, L., Fu, W., Yu, L., et al. Comparative effect of Liuzijue Qigong and conventional respiratory training on trunk control ability and respiratory muscle function in patients at an early recovery stage from stroke: a randomized controlled trial. *Arch Phys Med Rehabil,* **102**, 423–430 (2021).
25. Cai, W. Effects of seated Baduanjin on the quality of life of community stroke patients with sequelae. *Chinese Nursing Research,* **24**, 2667–2668 (2010).
26. Chen, J. W., Chen, Q., Chen, C., Li, S. Y., Liu, L. L., Wu, C. S., et al. Effects of modified Baduanjin physical activity on cardiopulmonary function motor function and daily living activities in stroke patients. *Chinese Journal of Rehabilitation Theory and Practice*, **30**, 74–80 (2024).
27. Du, J., Yu, H., Xu, Q., Zhang, F., Zhao, S. T., Ou, Z. Y., et al. Effects of seated and lying Liuzijue on depression and self-efficacy in post-stroke patients with mild to moderate depression. *Journal of Nursing(China)*, **28**, 70–73 (2021).
28. Fu, C. X. & Zhang, Q. Y. Effects of Tai Chi on balance and walking ability in stroke patients with hemiplegia. *Chinese Journal of Rehabilitation Medicine,* **31**, 536–539 (2016).
29. He, J., Wang, W., Li, K. P., Su, J. Q., Wang, X. L., Feng, W., et al. Effects of six-form Tai Chi training on postural balance in stroke patients. *Chinese Journal of Rehabilitation Medicine,* **37**, 482–487 (2022).
30. Jiang, S. Z., Chen, J. X., Lu, W. N. & Jin, X. C. Application of Tai Chi Yunshou in upper limb rehabilitation of stroke patients with hemiplegia. *Chinese Journal of Nursing Education,* **15**, 219–222 (2018).
31. Li, Y. L., Hu, X. J. & Cui, L. N. Clinical observation on the use of seated Tai Chi exercise in 30 post-stroke depression patients. *Chinese Nursing Research*, **26**, 2254–2256 (2012).
32. Liu, W., Zhao, Y., Yang, D., Fu, Q. R., Zhou, J. & Yan, Z. Effects of Baduanjin exercise prescription on dynamic balance in stroke recovery patients. *Lishizhen Medicine and Materia Medica Research*, **33**, 1936–1939 (2022).
33. Sun, P. P., Fang, L., Qi, R., Chen, X., Yan, J. T. & Lei, F. Clinical efficacy and brain network mechanism of modified Yijinjing in post-stroke patients with mild depression. *Chinese Journal of Rehabilitation Medicine,* **37**, 1506–1510 (2022).
34. Tang, Q., Wang, X., Li, B. Y., Sha, S. & Zhu, L. W. Effects of modified Tai Chi on gait balance and fall efficacy in stroke recovery patients with hemiplegia. *Chinese General Practice,* **25**, 1857–1862 (2022).
35. Wang, C., Zhang, P. Z., Yang, F. M. & Yu, J. W. Effects of Liuzijue training on balance and respiratory function in stroke recovery patients. *Rehabilitation Medicine,* **32**, 306–313 (2022).
36. Wang, W. H., Zhang, G. P., Xie, H. J., Qu, P. Y. & Wang, X. H. Effects of seated Tai Chi on upper limb motor function in Brunnstrom stage II stroke patients. *Journal of Chengdu Sport University,* **49**, 82–87 (2023).
37. Wang, X. B., Hou, M. J., Tao, J., Lin, L. L. & Rao, T. Effects of Tai Chi Yunshou on gait in stroke patients with hemiplegia. *Chinese Journal of Rehabilitation Medicine,* **31**, 1328–1333 (2016).
38. Xia, J. Y., Chen, Z., Chen, Y. J., Zhang, Q., Chen, Y. Y. & Pei, S. Effects of Liuzijue training on post-stroke spastic dysarthria. *Journal of Audiology and Speech Pathology,* **29**, 271–275 (2021).
39. Yang, H. X. & Liu, X. L. Effects of Tai Chi and Baduanjin on lower limb motor function and electromyography in stroke patients with hemiplegia. *Chinese Journal of Rehabilitation Theory and Practice,* **25**, 101–106 (2019).
40. Zhang, F. L., Shu, H. Y., Ren, L. L., Yan, C., Liu, J. W. & Miao, G. X. Effects and brain mechanism of modified Wu Qin Xi on gait and balance disorders in elderly stroke patients. *The Journal of Practical Medicine,* **39**, 1174–1178 (2023).
41. Zhang, L., Hu, J., Xue, X. & Jin, X. M. Effects of modified Yijinjing on sleep quality in stroke recovery patients with sleep disorders. *Rehabilitation Medicine,* **30**, 74–78 (2020).
42. Zhang, L. L. & Huang, C. X. Effects of Baduanjin rehabilitation training on limb function, daily life, and quality of life in elderly stroke hemiplegia patients. *Chinese Journal of Gerontology*, **41**, 4620–4622 (2021).
43. Zhao, B., Tang, Q., Wang, Y., Zhu, L. W., Yang, H. X. & Ye, T. Effects of Tai Chi on motor function and depression in post-stroke patients with depression. *Chinese Journal of Rehabilitation Theory and Practice,* **23**, 334–337 (2017).
44. Zheng, Z. H., Huang, Y. F., Chen, F. F., Chen, G. L., Wang, J. & Lu, K. Y. Effects of He Zi Jue on left heart function and quality of life in patients with ischemic stroke. *Chinese Nursing Research,* **37**, 4314–4320 (2023).
